# The liverwort *Pellia endiviifolia* shares microtranscriptomic traits that are common to green algae and land plants

**DOI:** 10.1111/nph.13220

**Published:** 2014-12-19

**Authors:** Sylwia Alaba, Pawel Piszczalka, Halina Pietrykowska, Andrzej M Pacak, Izabela Sierocka, Przemyslaw W Nuc, Kashmir Singh, Patrycja Plewka, Aleksandra Sulkowska, Artur Jarmolowski, Wojciech M Karlowski, Zofia Szweykowska-Kulinska

**Affiliations:** 1 Bioinformatics Laboratory, Institute of Molecular Biology and Biotechnology, Faculty of Biology, Adam Mickiewicz University61-614, Poznań, Poland; 2 Department of Gene Expression, Institute of Molecular Biology and Biotechnology, Faculty of Biology, Adam Mickiewicz University61-614, Poznań, Poland

**Keywords:** basal lineage, land plants, liverwort, microRNA (miRNA) genes, microtranscriptome, miRNA precursors, miRNA targets, *Pellia endiviifolia*

## Abstract

- Liverworts are the most basal group of extant land plants. Nonetheless, the molecular biology of liverworts is poorly understood. Gene expression has been studied in only one species, *Marchantia polymorpha*. In particular, no microRNA (miRNA) sequences from liverworts have been reported.
- Here, Illumina-based next-generation sequencing was employed to identify small RNAs, and analyze the transcriptome and the degradome of *Pellia endiviifolia*.
- Three hundred and eleven conserved miRNA plant families were identified, and 42 new liverwort-specific miRNAs were discovered. The RNA degradome analysis revealed that target mRNAs of only three miRNAs (miR160, miR166, and miR408) have been conserved between liverworts and other land plants. New targets were identified for the remaining conserved miRNAs. Moreover, the analysis of the degradome permitted the identification of targets for 13 novel liverwort-specific miRNAs. Interestingly, three of the liverwort microRNAs show high similarity to previously reported miRNAs from *Chlamydomonas reinhardtii*.
- This is the first observation of miRNAs that exist both in a representative alga and in the liverwort *P. endiviifolia* but are not present in land plants. The results of the analysis of the *P. endivifolia* microtranscriptome support the conclusions of previous studies that placed liverworts at the root of the land plant evolutionary tree of life.

## Introduction

Land colonization by plants probably began in the Early Middle Ordovician period, and liverworts are the most likely basal group of land colonizers (Supporting Information Fig. S1) (Qiu *et al*., 2007; Rubinstein *et al*., 2010; Ruhfel *et al*., 2014). Embryophytes are generally accepted to have originated from charophycean green algae, similar to extant charophytes (Charales, Coleochaetales or Zygnematales). Thus, charophytes are probably the closest living green algal relatives to liverworts (Karol *et al*., 2001; Qiu *et al*., 2007; Timme *et al*., 2012; Bowman, 2013). The basal status of liverworts as land plants suggests that they share common molecular traits linking algae and vascular plants.

Liverworts (phylum Marchantiophyta) are divided into three classes: Haplomitriopsida, Jungermanniopsida, and Marchantiopsida. The Jungermanniopsida represent > 80% of living liverwort taxa, which include the dioecious *Pellia endiviifolia* (subclass Pelliidae, order Pelliales). The representatives of the genus *Pellia* are recognized as the most basal lineage of the simple thalloid liverworts with respect to many plesiomorphic features, such as cuneate apical cell, a thallus without a midrib, a spherical capsule and massive seta (Pacak *et al*., 1998; He-Nygrén *et al*., 2006; Crandall-Stotler *et al*., 2009). Only a few examples of the regulation of gene expression have been reported in liverworts (Qiu *et al*., 1998; Nishiyama *et al*., 2000; Yamato *et al*., 2007; Sierocka *et al*., 2011; Sharma *et al*., 2013, 2014). However, none of these studies were devoted to small RNAs (sRNAs), noncoding 19–25-nucleotide (nt)-long RNA molecules that are of particular interest as key regulators of eukaryotic gene expression (Voinnet, 2009; Khraiwesh *et al*., 2010). MicroRNAs (miRNAs) are a class of sRNAs that are transcribed by RNA polymerase II. Primary miRNA transcript (pri-miRNA) derived from the *MIcroRNA* (*MIR*) gene typically forms an imperfect fold-back structure that is processed into a stem-loop precursor (pre-miRNA) and further excised as an miRNA/miRNA* duplex (Kim, 2005). The mature miRNA molecule is then incorporated into an effector complex named the RNA-induced silencing complex (RISC) to guide target mRNA cleavage or translation inhibition (Lee *et al*., 2004; Tang *et al*., 2003; Brodersen *et al*., 2008). The final output of miRNA activity is the down-regulation of expression for a given protein.

The first sRNA studies which were performed in *Arabidopsis thaliana* and *Oryza sativa* discovered miRNA families, the majority of which were conserved between these plant species (Llave *et al*., 2002; Park *et al*., 2002; Reinhart *et al*., 2002; Jones-Rhoades & Bartel, 2004; Sunkar & Zhu, 2004). However, the experimental approach was based on low-depth sequencing or genome-based computational predictions of fold-backs and complementary target sequences, which preferentially captured the most abundant miRNAs belonging to conserved families. The development of high-throughput sequencing technologies has permitted an increase in the sequencing depth for various angiosperm species. This technology also opened a window of opportunity to broaden miRNA research beyond the scope of spermatophytes and permitted the exploration of the miRNA repertoire in nonseed plants. Comparative studies on the miRNA repertoire of the representatives of three evolutionarily distant plant lineages (the moss *Physcomitrella patens*, the lycopod *Selaginella moellendorffii* and the angiosperm *A. thaliana*) have revealed that only a small fraction of the identified miRNAs are conserved and highly expressed across basal plants and angiosperms. The majority of miRNAs in both mosses and lycopods tend to be lineage-specific, exhibiting features that are characteristic of young, recently evolved miRNA species in *A. thaliana*: they are weakly expressed, tend to originate from a single genomic locus and have few predicted mRNA targets (Axtell *et al*., 2007; Fattash *et al*., 2007; Banks *et al*., 2011). The analyses of combined high-throughput sequencing data sets for different representatives of embryophytes have identified eight miRNA families that are common to all of the land plants studied thus far. Ten families are present in all angiosperm lineages, while the other families display more restricted taxonomic distributions. Moreover, a high proportion of species-specific miRNA genes have been observed not only in *P. patens* and *S. moellendorffii* but also in rice, *Medicago truncatula* and *Glycine max* (Cuperus *et al*., 2011). The predominance of young miRNA families with restricted taxonomic distribution, which is characteristic of unique groups of plants, strongly supports the hypothesis that the *MIR* genes are born and lost at a high frequency. Nonconserved miRNAs represent an evolutionarily flexible set of regulatory molecules that can appear and disappear on relatively short evolutionary time-scales with no or minor functional consequences, and with only a few being stabilized during their brief existence by recruitment into beneficial regulatory interactions (Rajagopalan *et al*., 2006; Fahlgren *et al*., 2007, 2010; Cuperus *et al*., 2011).

Interestingly, no miRNA families that have been identified in land plants have been discovered to date in the unicellular green alga *Chlamydomonas reinhardtii* or the colonial green alga *Volvox carteri*. The lack of universally conserved miRNA genes between land plants and green algae suggests that miRNA genes have evolved independently in these two eukaryotic lineages (Molnar *et al*., 2007; Zhao *et al*., 2007; Li *et al*., 2013). However, attention should be paid to the effect of disparities in taxonomic sampling on our understanding of miRNA evolution. Entire divisions either have no coverage (Marchantiophyta, Anthocerophyta, Psilophyta, Sphenophyta, Cycadophyta, Ginkgophyta, and Gnetophyta) or have had only a single taxon surveyed (Bryophyta, Lycophyta and Pteridophyta) (Taylor *et al*., 2014).

Here, we present the first analysis of the liverwort miRNA repertoire (microtranscriptome) from *P. endiviifolia*, which enables the identification of conservative plant miRNAs and new, liverwort-specific miRNAs. Combined analyses of the transcriptome, sRNAs and degradome data provide experimental evidence for a target mRNA turnover by identified conserved and novel miRNAs. Moreover, we demonstrate for the first time the existence of common miRNAs between algae and land plants, supporting the hypothesis of liverworts being the basal lineage of land plant evolution.

## Materials and Methods

### Plant material

Female and male thalli of *Pellia endiviifolia* (Dicks.) Dumort. producing sex organs were collected from Kopanina, Poznan, Poland (herbarium number 40 228 in POZW) from 2006 to 2012 in September–December. This approach permitted experiments to be conducted on a large range of *P. endiviifolia* material. The plants that were collected during the three 2006, 2007 and 2008 seasons were used to start an *in vitro* collection on mineral medium (Fiedorow & Szweykowska-Kulinska, 1998) and subsequently grown on half-strength Gamborg medium (Sigma-Aldrich). Plants from axenic culture were grown under continuous light from white fluorescent lamps at 21–23°C. Plant material that is collected in the field can potentially contain endophytic fungi and algae. To check for the presence of biological contamination in *P. endiviifolia* thalli that were collected from natural habitats and grown *in vitro* (Fiedorow *et al*., 2001), aniline blue-stained thalli were examined for the presence of fungal hyphae using a Zeiss Axioskop 2 plus microscope that was equipped with a Power Shot G5 Canon Digital Camera (Liberato *et al*., 2005). Fig. S2 shows the presence of endophytic fungi in the *P. endiviifolia* thalli cells that were collected from the natural habitat. No hyphae were observed in the case of the *P. endiviifolia in vitro* culture. Additionally, we tested for the presence of fungal and algal DNA using PCR with primers that were specific for the amplification of the *ribosomal protein S11* (*rps11*)–*ribosomal protein L2* (*rpl2*) plastid gene cluster across Chlorophyta species and primers for the amplification of a portion of the small subunit rRNA that is specific to arbuscular endomycorrhizal fungi (Table S1) (Simon *et al*., 1992; Provan *et al*., 2004). The PCR analysis showed that *P. endiviifolia* thalli that were collected from the natural habitat contained DNA products that are specific for fungi and algae, while the thalli that were grown *in vitro* did not harbor any detectable traces of fungal or algal DNA (Fig. S3). Therefore, we considered the *P. endiviifolia* thalli that were grown *in vitro* as fungus- and alga-free. Subsequently, the identification of conservative miRNAs was approved only if a given miRNA sequence was identified in the next-generation sequencing (NGS) data derived from liverwort thalli that were introduced and grown *in vitro* and for new miRNAs when they were also detectable in northern hybridization when RNA was derived from *P. endiviifolia* axenic culture.

### RNA and DNA isolation

Total RNA for sRNA detection was isolated using a method that permits the enrichment of sRNAs (Kruszka *et al*., 2013) with several modifications (see Methods S1).

### Northern blot analysis of mature miRNAs

The procedure for the northern blot analyses has been previously described (Szarzynska *et al*., 2009). Up to 60 μg of RNA enriched in sRNAs per line was loaded on denaturing polyacrylamide gels. The oligonucleotide sequences that were used as probes in the northern analyses are shown in Table S1.

### pri-miRNA RACE experiments and genome walking

Detailed information about rapid amplification of cDNA ends (RACE) and genome walking experiments is included in Methods S1.

The nucleotide sequences obtained in this study have been deposited in GenBank under the following accession numbers: KM001698–KM001707.

### Deep-sequencing and bioinformatic analyses

Total RNA was isolated from the male antheridia-producing gametophytes and female archegonia-producing gametophytes that were collected from the field and from both female (twice) and male thalli that were grown *in vitro*, which had no reproductive organs. Isolated RNA was used for the construction of five independent sRNA libraries. The sRNA libraries were generated using a modified protocol as previously described (Pant *et al*., 2009). Adaptor sequences were identified and trimmed from each read using a customized Perl script. More detailed procedures for library preparation and bioinformatic analyses are included in Methods S1. The sRNA and transcriptome sequencing data are available in the National Center for Biotechnology Information (NCBI) Sequence Read Archive database under accession numbers SRP043263 and SRP048691.

### Transcriptome and degradome sequencing

To ensure the best representation of the *P. endiviifolia* transcriptome and a sufficient variability of plant material, five different liverwort developmental stages from two growth conditions were used for total RNA isolation: antheridia-producing male gametophytes, archegonia-producing female gametophytes, sporophytes that were collected from the field and female and male thalli that were grown *in vitro* with no reproductive organs. DNA was removed by digestion using RNase-free TURBO™ DNase (Ambion, Austin, TX, USA). The lack of genomic DNA contamination was confirmed by PCR using primers that were designed for the *P. endiviifolia S-Phase Kinase-Associated Protein 1* (*SKP1*) gene (accession number FJ266076) promoter sequence. RNA integrity was confirmed on 1.2% agarose gels before and after DNase digestion. RNA isolates were combined, and a high-quality RNA sample (22 μg) was shipped on dry ice to the Beijing Genome Institute (BGI) (Beijing, China) for cDNA library preparation and sequencing. cDNA transcriptome library preparation and pair-end (PE) 90-nt NGS were performed on an Illumina HiSeq 2000 system (Illumina Inc., San Diego, CA, USA). PE-read sequence quality estimation was performed by the BGI to permit reliable transcriptome assembly. The assembled transcript data quality was assessed by sequence annotation using the NCBI nr database.

The same total RNA isolation procedure was used to prepare degradome libraries. The degradome libraries were prepared as previously described (Addo-Quaye *et al*., 2009a,b; German *et al*., 2009). For more detailed information, please see Methods S1. The final libraries of 26-27-mer 5′-ends of the 5′-end phosphorylated mRNAs were sequenced by Fasteris (Plan-les-Ouates, Switzerland) on Illumina HiSeq 2500, with the number of cycles being 1 × 50 + 7, using TruSeq SBS Kit v3 (Illumina). The adapters were trimmed with the cutadapt program (minimum overlap = 19), and only reads with identified adapters that were longer than 13 nt were selected for subsequent analyses.

Detailed analysis of the transcriptome and degradome data will be published in a separate paper.

### Identification of new miRNAs from the NGS results

Because of the specificity of the experimental model, we developed a new, non-reference-based computational approach to identify novel miRNAs. The procedure involved the creation of grouped sequence reads based on sequences that were aligned perfectly to each other, which we termed ‘clusters’; size and expression profiling of clustered reads; and the identification of functional sRNA features that are characteristic for previously described sequences from other plants (GC content and first nucleotide occupation).

From the mapping results, 204 979, 279 332, 361 479, 495 396 and 284 390 18–26-nt clusters of a minimum of two sRNAs in each group were built for all five sRNA-seq samples. Each cluster was then described as a sequence-length profile, where counts of reads of the same size were summarized. Abundance-ranked clusters with a read size distribution similar to that of known miRNAs and with a clearly dominant signal, preferably at 20–21-nt read size, were selected for further analyses. To address the possible origin of the sRNA sequences and to remove contamination, the clustered reads were compared with the Rfam and NCBI databases representing sequences from Bacteria, Archaea, Glomeromycota fungi and Chlorophyta. For experimental validation, based on the cluster read size profile, abundance, GC content and overall nucleotide composition, we selected the 71 top unannotated clusters that were present in at least one of the *in vitro* libraries.

### miRNA target identification

To select transcripts for target prediction, we performed protein coding sequence identification using the ESTScan program (Iseli *et al*., 1999). The program was trained on *P. patens* coding sequences that were obtained from the NCBI database to build codon usage matrices for *P. endiviifolia* prediction. The number of isochores was set to one, as suggested by the ESTScan developers. The target identification procedure was based on three publicly available programs: miRanda,tapir, and psRNAtarget (John *et al*., 2004; Bonnet *et al*., 2010; Dai & Zhao, 2011). All of the protein coding transcripts from *P. endiviifolia* that were predicted by ESTScan were used as the input. The results produced by the prediction software were subsequently scored based on the mismatches that were present in the predicted sRNA:target duplex alignments, where G:U pairing was counted as 0.5 point, and other mismatches were given one point according to Jones-Rhoades & Bartel (2004) and Schwab *et al*. (2005). Duplexes with a total mismatch score of ≥ 4 and a substitution score in positions 1–12 of sRNA > 2.5 points were discarded. All of the predicted targets that passed the mismatch score filtering were passed for further cleavage site evaluation based on the degradome data.

A degradome analysis was performed on predicted targets using target plots (T-plots) (German *et al*., 2008). For each target nucleotide position, the sum of the tagged 5′-ends that were aligned to the position was calculated. All of the tag counts were normalized by the library size, multiplied by one million to represent tags per million (TPM) and then divided by the total number of transcripts that they were mapped to, as suggested by Addo-Quaye *et al*. (2009a,b). The cleavage signal threshold was calculated for each T-plot, representing the mean TPM counts from the total tags that were aligned to the transcript. Signals greater than the mean TPM counts were selected for further analysis. The putative cleavage signal position that was indicated by the tags was compared with the predicted 9–11 position of the miRNA:target duplex. T-plots with a tag position that matched the predicted cleavage site of the miRNA:target duplex were analyzed manually and divided into three groups based on their TPM value compared with other tag TPM values and its positional proximity.

Protein domain prediction analysis using the hmmer3 tool was performed on targets with degradome-confirmed cleavage sites (Eddy, 2011). The functional annotation of the predicted targets was based on protein families database (Pfam) gene ontology annotations and/or best protein hits that were extracted from BLASTx results in the nr NCBI database (Altschul *et al*., 1997; Sonnhammer *et al*., 1997). Homologous proteins corresponding to the targets with a similarity that was equal to or lower than an *E*-value of 0.001 with at least 70% coverage of the known protein were selected.

## Results

### Liverwort *P. endiviifolia* shares conservative miRNAs with land plants

To identify miRNAs in *P. endiviifolia*, five independent sRNA libraries reflecting four different *P. endiviifolia* thalli were sequenced with the high-throughput Illumina system. After filtering out the adapter sequences and sequences with a low quality or low copy number, only those sequences in the range of 18–26 nt were selected, giving final reads of 1880 538, 3389 639 and 3133 111 for one male and two female sRNA *in vitro* libraries and 5048 417 and 3448 623 for one male and one female sRNA environmental library, respectively. A dominating class of 21-nt-long reads was observed for all libraries, which is consistent with the typical sRNA distribution of angiosperms, such as barley (*Hordeum vulgare*) (Kruszka *et al*., 2013), rice (*Oryza sativa*) (Morin *et al*., 2008) and cucumber (*Cucumis sativus*) (Mao *et al*., 2012). A maximum of two nucleotide mismatches within the overlapping regions were allowed when conservative miRNAs were selected from the NGS results using known plant mature miRNA sequences as a query.

**Figure 1:**
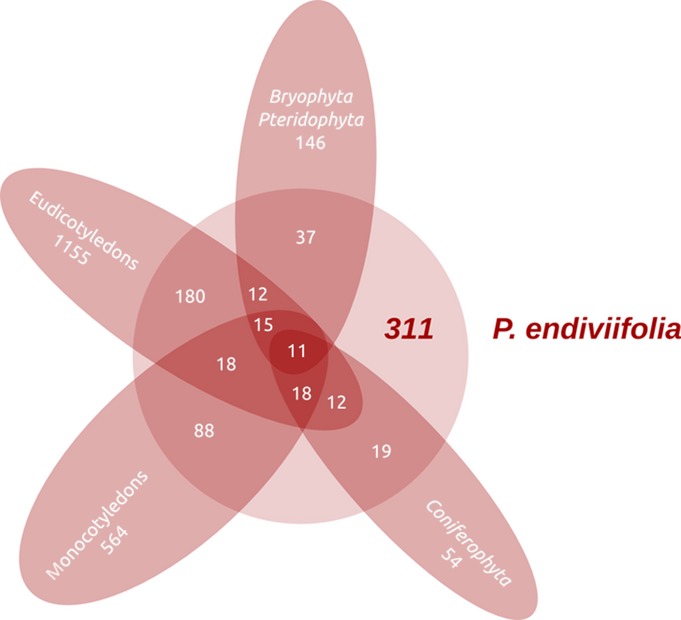
Venn diagram showing the identification of *Pellia endiviifolia* conservative microRNA (miRNA) families within land plants according to miRBase [http://www.mirbase.org/].

**Figure 2:**
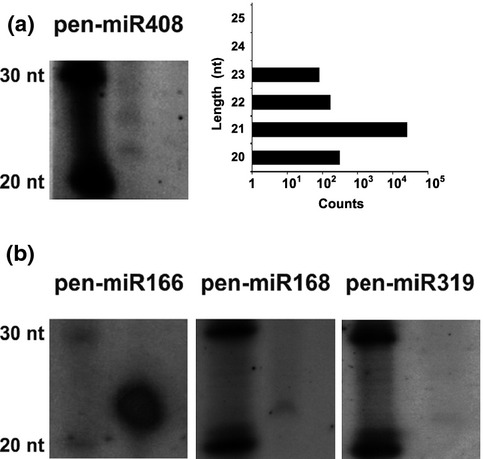
Selected conservative microRNAs (miRNAs) in the liverwort *Pellia endiviifolia* as detected by northern hybridization. Above the blots, the name of each miRNA is provided along with the length of the dominating cDNA fragment in a given cluster and the mean counts (k, kilo) from all of the deep-sequencing results for all family members (up to two mismatches in the overlapping region to the probe sequence were considered; see Supporting Information Table S2). (a) pen-miRNA 408. The graph on the right presents the distribution of reads within the miR408 cluster. The read counts represent a single next-generation sequencing (NGS) experiment. (b) miRNA166, miR168, and miR319. The left side of the northern blots shows the RNA decade marker depicting 30-nucleotide (nt)- and 20-nt-long RNAs.

Altogether, 311 of the miRNA families that were identified in *P. endiviifolia* were also present in land plant representatives that have been studied to date (Table S2; Fig.1). Overall, 25.3% of the known miRNA families that are specific for Bryophyta and Pteridophyta, 35.2% of those that are specific for Coniferophyta, 15.6% of those that are specific for eudicotyledons and 15.6% of those that are specific for monocotyledons were identified in the liverwort *P. endiviifolia* when miRBase release 20 was used as a reference for miRNA sequences (Griffiths-Jones, 2004; Kozomara & Griffiths-Jones, 2014). Eleven conserved miRNA families are common to all land plants (Table1). The miR156 (a–j), miR160 (a–c), miR166 (a–g), and miR390 (a, b) multimember families are represented in *P. endiviifolia* by all of the members that have been identified in *A. thaliana*. By contrast, rice miR156, miR160 and miR166 families are more complex and contain additional members that were not identified in *P. endiviifolia*. Only three members of the miR156 family have been found in *P. patens* (miR156a–c), a representative of bryophytes, while the *P. patens* miR160 family is represented by a higher number of miRNA species (miRNA160a–i) (Kozomara & Griffiths-Jones, 2014). The quality and reliability of the sequencing results were confirmed by northern hybridization, which was performed for selected conservative miRNAs that were selected based on their count number and northern hybridization signal strength. Fig.2(a) shows the hybridization results for *P. endiviifolia* miR408-5p (pen-miR408-5p), and a corresponding graph presenting the read counts for the miR408-5p cluster (see the Materials and Methods section). Fig.2(b) shows the northern blots for the three additional selected conservative miRNAs (pen-miR166, pen-miR168, and pen-miR319) with the closely corresponding number of NGS reads for 21-nt-long species and the intensity of the hybridization signals (for other selected conservative miRNAs, see Fig. S4). The identified repertoire of *P. endiviifolia* miRNA families indicates that this liverwort shares the majority of its miRNAs with other land plants.

**Table 1:** Common land plant microRNAs (miRNAs) that were identified in *Pellia endiviifolia*

miR family	miR member	Sequence	Length (nt)	miR length (nt)	Mn	Mean count
ath-miR156	a	tgacagaagagagtgagcac	20	21	0	2247.09
ath-miR156	g	ggacagaagagagtgagcac	20	20	0	13.01
vvi-miR156	h	ttgacagaagagagagagcat	21	20	1	5.73
ath-miR156	i	tgacagaagagagagagca	19	21	0	1.92
ath-miR156	j	tgacagaagagagagagcac	20	21	0	4.43
ath-miR159	a	tttggattgaagggagct	18	21	0	1.28
ath-miR160	a	tgcctggctccctgtatgcca	21	21	0	3125.22
ath-miR166	a	tcggaccaggcttcattcccc	21	21	0	309522.05
osa-miR171	i-5p	aggtattggcgcgcctcaatt	21	21	1	0.97
ath-miR319	a	cttggactgaagggagctcccttt	24	21	0	8.34
ath-miR390	a	aagctcaggagggatagcg	19	21	0	235.63
bdi-miR395	d	tccaagtgtttcgggtactctagg	24	21	2	1.77
ath-miR396	b	ttccacagctttcttgaactt	21	21	0	13.79
ath-miR408		atgcactgcctcttccctggc	21	21	0	388.61
osa-miR535	-5p	tgacaacgagagagagcacgc	21	21	0	4374.19

ath, *Arabidopsis thaliana*; bdi, *Brachypodium distachyon*; vvi, *Vitis vinifera*; osa, *Oryza sativa*; Mn, number of mismatches in sequence-overlapping regions.

### Novel liverwort-specific miRNAs

**Figure 3:**
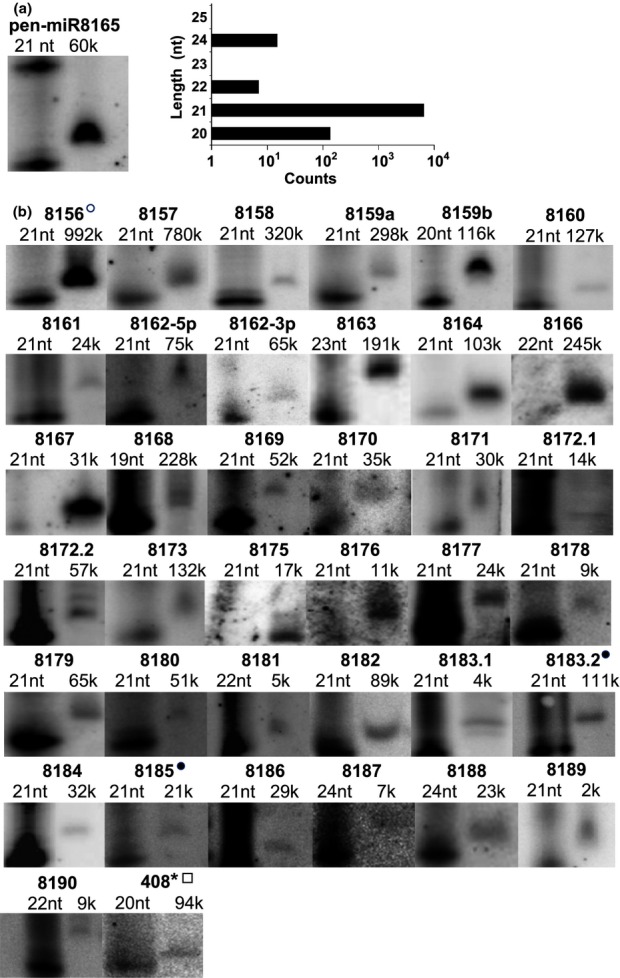
Forty-two novel microRNAs (miRNAs) in the liverwort *Pellia endiviifolia* as detected by northern hybridization. Above the blots, the length of the dominating cDNA fragment in a given cluster and the mean counts (k, kilo) from all of the deep-sequencing results are provided. (a) pen-miRNA8165. The graph on the right shows the distribution of the reads within the pen-miR8165 cluster. The read counts represent a single next-generation sequencing (NGS) experiment. (b) Additional novel pen-miRNAs. The left side of the northern blots shows the RNA decade marker depicting a 20-nucleotide (nt)-long RNA. The numbers above the blots represent the numerical part of the novel miRNA ID. Open circle, pen-miR8156a, pen-miR8156b, pen-miR8156c and pen-miR8156d form one miRNA family and differ from each other by 1 nt; closed circles, sRNA homologs that were found in the *Chlamydomonas reinhardtii* NGS data with one and two mismatches in the overlapping regions. Open square, pen-miR408-5p*. miR408-5p* was originally identified as a novel miRNA and then identified as miR* in pre-miRNA408-5p.

Data from the previously sequenced plant genomes indicate that the majority of identified miRNAs belong to nonconserved or species-specific miRNA families that are present only in a given plant family or species, respectively (Kozomara & Griffiths-Jones, 2014). A careful analysis of *P. endiviifolia* sRNA NGS data resulted in the identification of novel, potentially liverwort-specific miRNAs. Using an original approach, we selected a number of sRNA reads that fulfilled the criteria for potential miRNAs. Table2 shows the sequences of the 42 selected putative miRNAs (belonging to the 34 new miRNA families) that were found in our search, while Fig. S5 presents the distribution of the read length for each miRNA cluster (see the Materials and Methods section). In each cluster, there are dominating RNA species within a length range that is typical for miRNAs. We confirmed the presence of these potential miRNA species in *P. endiviifolia* using northern hybridization (Fig.3). All of the tested miRNA species gave a clear signal in the range of 19–23 nt in length, with the majority of the novel potential miRNA species being 21 nt. As a negative control for the hybridization-based evaluation, we selected an sRNA cluster without the domination of any particular RNA fragment size (see Fig. S5, control). In this case, the northern hybridization did not reveal any clear signal. The similarity search for the novel liverwort miRNA species in the sRNA NGS results and the genome of *P. patens* did not produce positive results. Thus, we conclude that the identified sRNAs represent liverwort-specific miRNAs.

**Table 2:** Novel, putative microRNAs (miRNAs) that were identified in *Pellia endiviifolia*

miRNA name	Sequence	Length (nt)	Mean count
pen-miR8156a	tctcccacacatcgtctagga	21	19879060.23
pen-miR8156b	tctcccacacatcgtctaggc	21	58077.70
pen-miR8156c	tctgccacacatcgtctagga	21	22901.49
pen-miR8156d	tcgcccacacatcgtctagga	21	9325.45
pen-miR8157	tttcccagacatcgtcgatga	21	2780091.24
pen-miR8158	tgaaggacgcattctgctcga	21	320926.69
pen-miR8159a	ttaccttgaagagtctggaag	21	298119.06
pen-miR8159b	ttaccttgaagagtctggaa	20	116532.20
pen-miR8160	tccctaaagactcgggcaata	21	127680.15
pen-miR8161	ttcgagcagagaggtggagcc	21	424963.43
pen-miR8162-5p	tttgtaagaattggaaccgga	21	75985.52
pen-miR8162-3p	tggatccatttcttacagacg	21	65120.30
pen-miR8163	ccccgtggaagaaaacaatctca	23	191569.82
pen-miR8164	caatttgtggcaaacttcccc	21	103523.36
pen-miR8165	ttgctagagaggactttcctt	21	59900.20
pen-miR8166	ttgctttgggagacatcagttc	22	245117.49
pen-miR8167	ccatgcctactatacccaatc	21	31174.82
pen-miR8168	ggggacgtagctcaatcgg	19	228305.93
pen-miR8169	tgcgataagaagggttgagct	21	52738.45
pen-miR8170	tgcttcacagaggagatgcat	21	35621.35
pen-miR8171	caatagaagcatgggactgag	21	30129.15
pen-miR8172.1	tcggacgttatcacgccttgg	21	14799.01
pen-miR8172.2	tctaagtcgggaagccaaggc	21	57485.07
pen-miR8173	ccagtaggagatgtgatcgta	21	132404.08
pen-miR8175	ccatgcctgctatatccaatc	21	17454.77
pen-miR8176	tctgtgtttcaggacctcaaa	21	11953.24
pen-miR8177	tctgatggactgcagggcatg	21	24212.20
pen-miR8178	tggggtcctataagtcatcaa	21	26231.57
pen-miR8179	tgggatacagtaggcataact	21	65510.02
pen-miR8180	tacagacttgcactcgtcgtc	21	51215.78
pen-miR8181	ggcccgtgcggtcggatggacc	22	5743.34
pen-miR8182	tctctcagtctggcacttaca	21	89872.87
pen-miR8183.1	gaaccgggactggaaggaggc	21	4846.39
pen-miR8183.2^a^	gggactggaaggaggctgaga	21	111970.03
pen-miR8184	tggaggaagagacattgtgac	21	32.62
pen-miR8185^a^	taaagatgatgggttttgttg	21	21593.70
pen-miR8186	cttgcagcaagcagatcccag	21	29270.63
pen-miR8187	acaaggtatgggaggtatggaatg	24	7878.44
pen-miR8188	agaaacgctgcaacggaacca	21	23546.63
pen-miR8189	aggaggatagtacagggttgt	21	2151.33
pen-miR8190	gagggttgcagagtggttttgg	22	9799.49
pen-miR408-5p^b^	ctagggtgaggcatggcatg	20	94669.44

a sRNA homologs that were found in *Chlamydomonas reinhardtii* NGS data with one and two mismatches in the overlapping regions;

b Primarily discovered as a novel miRNA; after the pre-miRNA structure prediction, it was identified as miR408-5p.

### *Pellia endiviifolia* contains miRNAs that are similar to the miRNAs that have been identified in the alga *C. reinhardtii*

Previous studies on the origin and evolutionary dynamics of miRNA genes indicated that no miRNA gene is currently shared between green algae and land plants (Cuperus *et al*., 2011; Nozawa *et al*., 2012). However, this previous analysis did not consider representatives of the phylum Marchantiophyta. Thus, we compared the data that were deposited in miRBase, as published by Molnar *et al*. (2007), Zhao *et al*. (2007), and Li *et al*. (2013), and sRNA NGS data for *C. reinhardtii* and *V. carteri* with our microtranscriptome sequencing data. These analyses, intriguingly, revealed the presence of three miRNAs (representing distinct families) from the liverwort *P. endiviifolia* with high sequence similarity to the sRNAs of the green alga *C. reinhardtii*. Up to two mismatches were permitted in this miRNA sequence similarity comparison. One of the miRNAs (pen-miR1444) was already annotated as cre-miR1144b, while two of the identified novel miRNAs in *P. endiviifolia* (pen-miR8183.2 and pen-miR8185) revealed the presence of highly homologous sRNAs within the deep sequencing data that were deposited for *C. reinhardtii* (which we reanalyzed) but not recognized by previous studies as miRNAs. Table3 shows the miRNAs that were found in *P. endiviifolia* species with high homology to green algal sRNAs.

**Table 3:** Three *Pellia endiviifolia* novel microRNAs (miRNAs) with homologous sRNA sequences that were found in *Chlamydomonas reinhardtii* next-generation sequencing (NGS) data

miR family	miR member	*P. endiviifolia* versus *C. reinhardtii* sequence	Length (nt)	Mn	Mean counts
pen-miR8183	.2	gg**ga**ctggaaggaggctgaga	21	2	111970.03
		**ac**ctggaaggaggctgag	18		
pen-miR8185		taa**a**gatgatgggttttgttg	21	1	21593.70
		aa**t**gatgatgggttttgt	18		
pen-miR1144	b	gtag**g**gtgg**a**ggcaggca	18	2	0.97
		tgggtag**t**gtgg**c**ggcaggcag	22		

One of the homologs represents the previously reported *C. reinhardtii* miRNA miR1144b. Short RNA sequences of *C. reinhardtii* homologs were identified in GSM803105, GSM803104 and GSM573523 samples from the NCBI GEO database. Mn, number of mismatches that were found in the overlapping regions. The bold letters indicate the mismatches that were present in overlapping region of the *P. endiviifolia-* and *C. reinhardtii-*aligned sequences.

**Figure 4:**
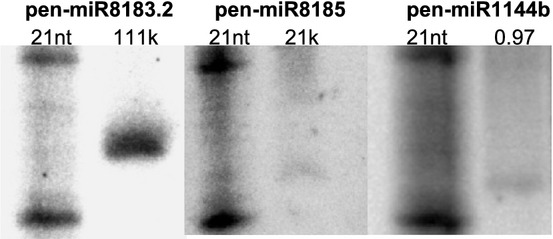
Selected *Pellia endiviifolia* microRNAs (miRNAs) that were homologous to *Chlamydomonas reinhardtii* miRNAs as detected by northern hybridization. The left side of the northern blots shows the RNA decade marker. Above the blots, the name of each miRNA is provided along with its length and mean counts (k, kilo). nt, nucleotide.

We confirmed the presence of the liverwort *P. endiviifolia* miRNAs that were homologous to *C. reinhardtii* miRNAs and sRNAs using northern hybridization (Fig.4). Total RNA for this experiment was isolated from *P. endiviifolia* thalli that were grown *in vitro* and were thus free from fungal and algal contamination (Figs S2, S3). Our results suggest that the two *C. reinhardtii* sRNAs may represent green algal miRNAs and belong to an miRNA category that is common for liverworts and green algae. To our knowledge, the *C. reinhardtii* and *V. carteri* miRNAs represent the only currently available source of green algal miRNAs.

Our results provide the first line of evidence for the existence of miRNA species that are conserved across algae and land plants and suggest that liverworts are an ancestral lineage for all embryophytes.

### Liverwort miRNA genes and precursors have a similar structure to those of other land plants

One of the fundamental features defining plant miRNAs is the precise excision of an miRNA/miRNA* duplex from the stem-loop pre-miRNA (Meyers *et al*., 2008). Stem-loop precursors are predicted using genomic DNA or known expressed sequence tags (ESTs)/transcripts as the input for RNA secondary structure prediction software. Because the genome of *P. endiviifolia* has not been sequenced, we performed transcriptome sequencing for *P. endiviifolia* (see the Materials and Methods section), the results of which were used as a reference database to search for miRNA precursors using the obtained *P. endiviifolia* miRNA data set as a query.

We found 26 pri-miRNA candidates. Some of the miRNAs are encoded by one pri-miRNA (miR536 and miR7732), and some are encoded by up to five different precursors (pri-miR408 encoding three mature miRNAs with different sequences), while the other miRNAs may be encoded within one precursor (pri-miR156 encodes two miRNAs (miRNA156i and miR156j), and pri-miR390 generates miR390 and miR390a). In total, nine pri-miRNAs encode nine conservative miRNAs, and 17 pri-miRNAs encode 15 novel miRNAs (Table S3; Fig. S6). The low number of identified unigenes representing potential miRNA primary transcripts can be explained by previously reported studies for angiosperms that show the fast and efficient processing of pri-miRNAs during miRNA biogenesis (Szarzynska *et al*., 2009; Bielewicz *et al*., 2012; Kruszka *et al*., 2013, 2014). The bioinformatic characterization of pri-miRNA candidates was validated using 5′ and 3′ RACE and genome walking using cDNA that was obtained from total RNA or genomic DNA that was isolated from *in vitro*-grown plants. Altogether, we were able to define the structure of 10 *P. endiviifolia* miRNA (*MIR*) genes and their precursors. These genes represent the first known liverwort *MIR* genes with fully characterized gene organization and transcripts with a predicted pre-miRNA stem-loop structure (Table4; Fig. S6). Among 10 *P. endiviifolia MIR* genes, two encode conservative miRNAs (miR408-5p and miR536), while eight encode newly identified liverwort-specific miRNAs. One of the *MIR* genes (*MIR* pen-8185) encodes the novel *P. endiviifolia* miRNA that was also identified in the *C. reinhardtii* sRNA library (Table4; Fig. S6) (Molnar *et al*., 2007). All of the genes that have been described represent independent transcriptional units containing one miRNA molecule that is embedded within a classical stem-loop structure (Fig. S6). Three *MIR* genes contain one intron, while one *MIR* gene contains two introns. All of these introns exhibit signatures that are typical of a U2-type intron. In all of the intron-containing *MIR* genes, the miR/miR* stem-loop structure was found in the last exon. This result is unusual compared with angiosperms, where *MIR* genes typically represent independent transcriptional units with an miRNA-containing hairpin structure that is located in the first exon (Szarzynska *et al*., 2009; Kruszka *et al*., 2013).

**Table 4:** Schematic representation of the identified *Pellia endiviifolia MIcroRNA* (*MIR*) genes and their precursors

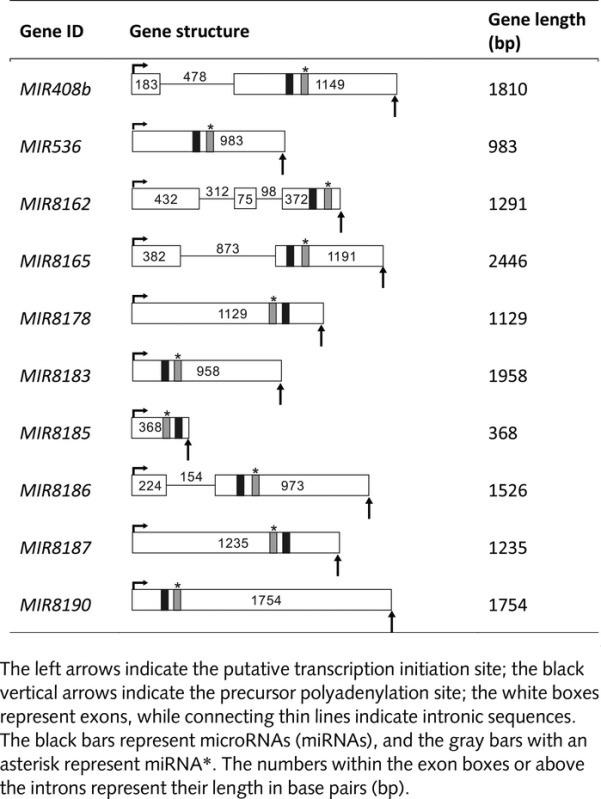

**Figure 5:**
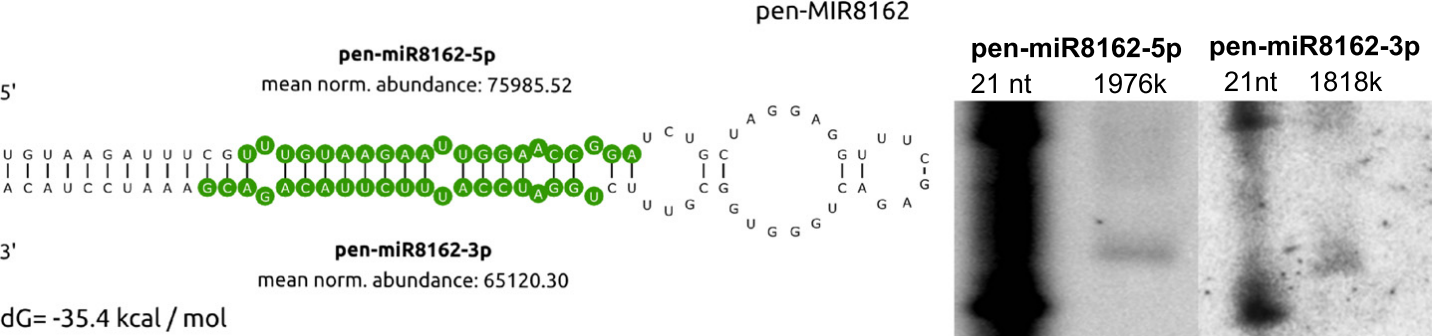
miR8162-5p and miR8162-3p derive from the same transcript, generating a stem-loop structure. The left panel shows the stem-loop structure of the pen-miR8162 precursor. Both of the microRNA (miRNA) species are marked in green because both are present at similar levels, and putative targets were found for both. The right panel shows northern hybridization for both miRNA species. nt, nucleotide; k, kilo.

Among the 42 newly identified *P. endiviifolia*-specific miRNAs, we found a pair of miRNA sequences (pen-miR8162-5p and pen-miR8162-3p) that are derived from the same transcript (Fig.5). Interestingly, both of these sequences were highly and equally represented in almost all of the sRNA data sets from both the NGS results and northern hybridization (Fig.5; Table S4). The alignment pattern of the sRNAs that were identified in the NGS results along with their corresponding transcript excludes the possibility of pen-miR8162-5p and pen-miR8162-3p representing a class of small interfering RNAs (siRNAs; data not shown). The high stability of both miRNA and miRNA* species indicates their possible functionality as miRNA, and in some of the previously published cases, such functions have been confirmed (Guo & Lu, 2010; Shao *et al*., 2013; Kruszka *et al*., 2013). Because both of these miRNAs are highly abundant in the same samples, they could play an important role in the cell by targeting actively transcribed genes. Therefore, putative targets for pen-miR8162-5p and pen-miR8162-3p have been predicted. The pen-miR8162-5p target encodes the putative transcription factor pen_C74-1, which contains the Myb/SANT -like DNA-binding domain (Myeloblastosis DNA-binding domain/acronym for ‘Swi3, Ada2, N-Cor, and TFIIIB’) that is specific for the guanine thymine 1 (GT-1) protein family of trihelix transcription factors. The predicted target of pen-miR8162-3p encodes a putative protein pen_U20592 containing the P-type ATPase ATP-binding domain and the haloacid dehalogenase-like hydrolase domain, suggesting that this target possesses nucleotide binding and hydrogenase activity. In the degradome data, we detected a very weak slicing product for the pen_U20592 transcript (data not shown) but were unable to confirm the cleavage site for the putative transcription factor mRNA.

### Conserved and new targets for *P. endiviifolia* miRNAs

**Figure 6:**
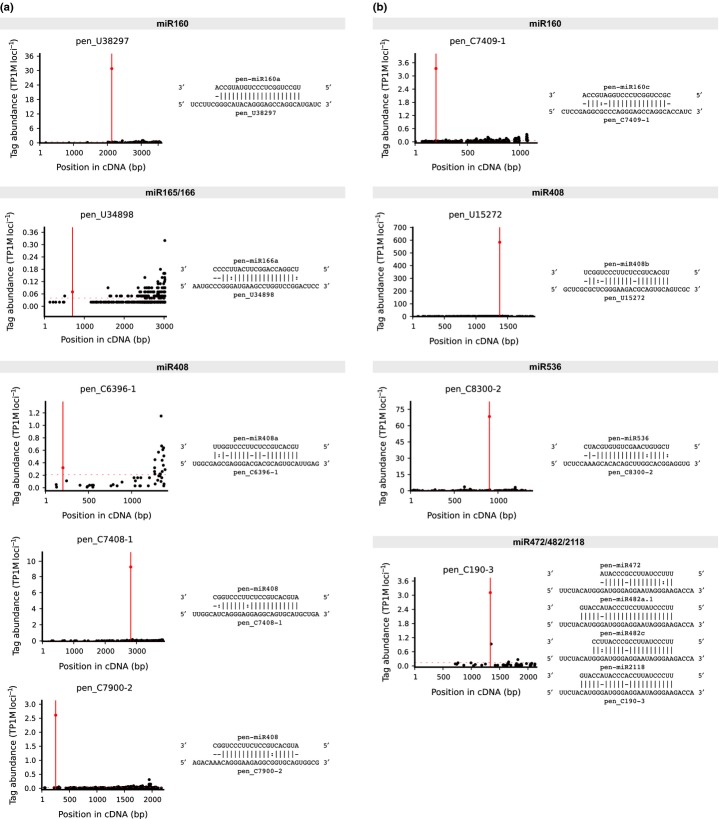
Target plots (T-plots) for *Pellia endiviifolia* microRNA (miRNA) targets. T-plots (German *et al*., 2008) are shown for known *P. endiviifolia* miRNA targets that are (a) conserved across the land plants and (b) new, validated using degradome data and corresponding to the targets that are presented in Table5. Each T-plot is accompanied by a duplex of miRNA and its target mRNA. For miR408 targeting homologous mRNAs and recognizing/cleaving the same sequence, only one example of each group of targets is shown (four T-plots). TP1M is the normalized abundance based on the formula TP1M = (raw abundance/library size) × 10^6^. Additionally, the normalized TP1M value was divided by the number of targets to which it perfectly matched. The red line indicates the position of the predicted cleavage site as confirmed by an aligned degradome tag.

The conservation of mature miRNA sequences between *P. endiviifolia* and other land plants raised the question of whether their corresponding target mRNAs had been preserved. For this analysis, we selected experimentally verified targets from plants whose microtranscriptomes were studied. As a result of the degradome data analysis, we found evolutionarily conserved mRNA targets for three miRNAs: miR160, miR166 and miR408 (Table5; Fig.6). Auxin Response Factor 16 (ARF 16) mRNA is targeted by miR160; phavoluta mRNA is targeted by miR166; and three different targets, plantacyanin, heavy metal ATPase 8, and ppa004802 m (found in *Prunus persica*), are all targeted by miR408 (Rhoades *et al*., 2002; Axtell *et al*., 2007; Abdel-Ghany & Pilon, 2008; Chorostecki *et al*., 2012; Zhu *et al*., 2012). All of the mRNAs were efficiently cleaved precisely in the predicted positions with respect to their cognate miRNAs. Moreover, for miR160 and miR408, we found new additional targets – one for each miRNA (see Table5; Fig.6). However, we were unable to show that the other conserved miRNAs target the homologous mRNAs as in the case of other studied land plants. In the transcriptome data, we found orthologous transcripts that are targeted by the members of 75 conserved miRNA families in embryophytes (data not shown). However, we did not find their cleavage products within the *P. endiviifolia* degradome data. For the conservative miRNA miR536, which is found in *P. patens* and vascular plants, as well as miR472, miR482, and miR2118, which were identified solely in vascular plants, one new target was found for each individual miRNA (Fig.6; Table5). Altogether, 65 new targets have been found for 70 conservative plant microRNAs. Table S5 presents all of the degradome results that were obtained for the evolutionarily conserved miRNAs.

**Table 5:** Known and novel targets of conservative microRNA (miRNA) families that were identified in *Pellia endiviifolia* and confirmed by the degradome data for the *in vitro* samples

miRNA family	miRNA name	Target	Homologous target	Description^a^	Cleavage site	Cleavage region
miR160	pen-miR160a,b,c,d,e,f,g	pen_U38297^b^	Auxin response factor (ARF) Pp1s339_47V6 (*Physcomitrella patens*)	Auxin response factor B3 (DNA binding)	2129	CDS
	pen-miR160c	pen_C7409-1	–	Dirigent-like protein	200	CDS
miR165/166	pen-miR165a	pen_U34898^b^	Phavoluta (PHV) AT1G30490 (*Arabidopsis thaliana*)	Homeobox (DNA binding); StAR-related lipid-transfer	706	CDS
	pen-miR166a,b,c,d,e,g,i,j,m					
miR408	pen-miR408a,b,d	pen_C6396-1^b^	Plantacyanin (ARPN) AT2G02850 (*Arabidopsis thaliana*)	Plastocyanin-like (Cu ion binding, electron carrier)	203	CDS
		pen_C6396-2^b^			203	CDS
	pen-miR408,a,d,e	pen_C7408-1^b^	Heavy metal ATPase 8 (HMA8) AT5G21930 (*Arabidopsis thaliana*)	P-type ATPase ATP binding domain^c^ (nucleotide binding); heavy-metal-associated (metal ion binding)	2814	CDS
		pen_C7408-2^b^			2786	CDS
	pen-miR408,a,b,d,e	pen_C7900-2^b^	ppa004802m (*Prunus persica*)	56-kDa selenium binding protein (SBP56)	252	5′ UTR
	pen-miR408-5p,d	pen_U15272	–	Common central domain of tyrosinase; Polyphenol oxidase middle domain	1375	3′ UTR
miR472/482/2118	pen-miR472	pen_C190-3	–	Nucleotide binding-ARC domain (acronym for ‘Apaf1, R proteins, and CED4’)	1336	CDS
	pen-miR482a.1, c					
	pen-miR2118					
miR536	pen-miR536,c	pen_C8300-2	–	–	902	3′ UTR

a Based on Pfam domains and gene ontology (GO) terms;

b Conserved miRNA targets;

c Cu-transporting P-type ATPase (HMA8) delivers Cu to plastocyanin in the thylakoid lumen. CDS, coding DNA sequence; StAR, steroidogenic acute regulatory protein.

**Figure 7:**
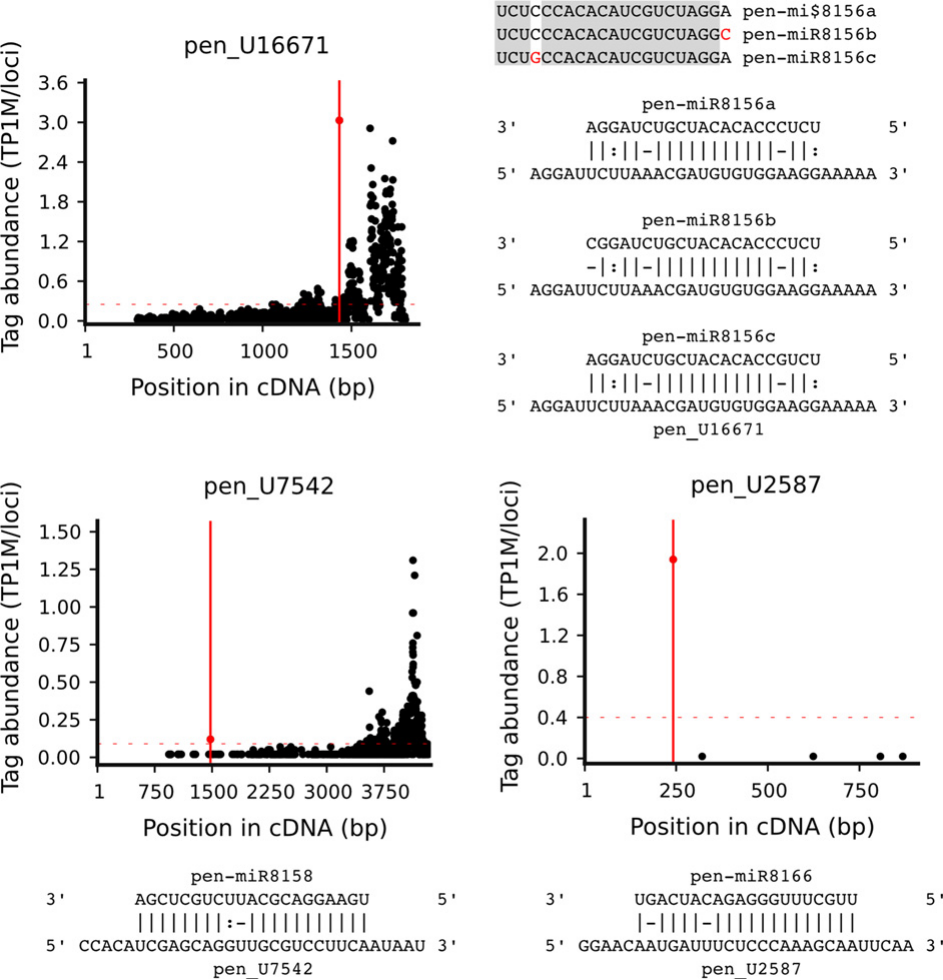
Target plots (T-plots) of validated target mRNAs for novel *Pellia endiviifolia* miRNAs using degradome data corresponding to the targets that are presented in Table6. The first T-plot shows the target coding sequences for three new microRNAs (miRNAs) representing a new miRNA family, pen-miR8156. The next two plots show the predicted slicing site for miR8158 and miR8166, representing the putative transcription factor mRNAs. TP1M abundance is normalized based on the formula TP1M = (raw abundance/library size) × 10^6^. Additionally, the normalized TP1M value was divided by the number of targets to which it perfectly matched. Each T-plot is accompanied by a duplex of miRNA and its target mRNA. The nucleotides that are shown in red represent nucleotide mismatches within the miR8156 family. bp, base pairs.

We also used the degradome data to search for mRNA targets for novel, liverwort-specific miRNAs. For 13 of 42 novel *P. endiviifolia* miRNAs, we found 14 target mRNAs. Two targets represent unknown sequences encoding putative proteins. For 12 of these targets, we were able to predict polypeptide sequences and annotate known protein domains (Table6). At least two of the targets contain domains that are typical for transcription factors (APetala2 and Auxin/Indole-3-Acetic Acid (AUX/IAA)). In the case of pen_U16671 cDNA, we found three miRNAs that recognized the same target site and represent a new family of miRNAs. In our data set, we also found a single miRNA (pen-miR8164) that targets three different mRNAs. These examples mirror very well the properties of the miRNA-target networks that have been reported in land plants (for a review, see Rubio-Somoza & Weigel, 2011). According to the gene ontology – a molecular function classification based on Pfam domain annotations – the newly identified targets for the conservative and novel miRNAs encode mostly proteins, nucleic acids, and nucleotide-binding proteins or proteins having various catalytic activities (data not shown). Fig.7 shows T-plots of validated target mRNAs for novel *P. endiviifolia* miRNAs using the degradome data that correspond to the targets that are shown in Table6. Additionally, Table S6 contains all of the degradome data that have been obtained for the newly identified miRNAs.

**Table 6:** Coding DNA sequence (CDS) targets of 13 novel microRNAs (miRNAs) from *Pellia endiviifolia* as confirmed by the degradome data from the *in vitro* samples

miRNA family	miRNA species	Target	Domain description^a^	Cleavage site	Cleavage region
pen-miR8156	a,b,c	pen_U16671	–	1432	CDS
	A	pen_U37136	Armadillo/beta-catenin-like repeat and U-box (ubiquitin-protein ligase)	1325	CDS
	B	pen_C1236-1	Nucleotide binding-ARC (ADP binding)	1229	CDS
pen-miR8158		pen_U7542	Auxin response factor, AUX/IAA, and B3 DNA binding	1479	CDS
pen-miR8159	a,b	pen_U15389	Polyketide cyclase/dehydrase and lipid transport	1346	3′ UTR
pen-miR8164		pen_C3350-1	–	3079	CDS
		pen_C4288-3	Cytochrome b5-like heme/steroid binding, oxidoreductase FAD/NAD/molybdopterin binding, and Mo-co oxidoreductase dimerization	2282	CDS
		pen_U7166	CBS/SIS and adenyl nucleotide/carbohydrate binding	1290	CDS
pen-miR8165		pen_U41210	BRCA1 C terminus (BRCT)	1978	CDS
pen-miR8166		pen_U2587^b^	APetala2 (DNA binding)	242	CDS
pen-miR8176		pen_C11045-1	TPR repeat	448	CDS
pen-miR8170		pen_U38378	HAUS augmin-like complex subunit 4	2062	3′ UTR
pen-miR8171		pen_U30616	PDZ^c^ (protein binding)	996	CDS
pen-miR8173		pen_U31475	NUDIX (hydrolase activity)	1074	CDS

a Based on Pfam domains and gene ontology (GO) terms;

b Transcript with weak similarity to known miR172 target AT4G36920 from *Arabidopsis thaliana*;

c Acronym for PSD-95 (a 95 kDa protein involved in signaling in the post-synaptic density), Dlg (the Drosophila discs large protein), and ZO1 (the zonula occludens 1 protein involved in maintaining epithelial cell polarity); gray color indicates miRNA-target examples for which T-plots are presented in Fig.7. BRCA1, BReast CAncer 1; CBS/SIS, Cystathionine Beta Synthase/Sugar Isomerase; HAUS, Human AUgmin-like complex Subunit; NUDIX, NUcleoside DIphosphate linked to X; TPR, Tetratrico Peptide Repeat superfamily.

## Discussion

Because liverworts are thought to represent a basal group of land plants and because *Pellia* species are recognized as the most basal lineage of the simple thalloid liverworts, we studied the *P. endiviifolia* microtranscriptome and compared it with miRNAs from green algae and land plants. Additionally, despite the dramatic increase in the number of identified miRNAs in vascular plants, there is little evidence of their occurrence in bryophytes and algae (Zhang *et al*., 2010; Kozomara & Griffiths-Jones, 2014). To our knowledge, no miRNAs have been identified for the liverwort phylum, and our study provides the first detailed analysis of the liverwort microtranscriptome. Altogether, we identified 486 miRNAs representing 345 families (311 conservative miRNA families and 34 novel miRNA families).

The number of identified conserved miRNA families in *P. endiviifolia* is high and encompasses families from all other land plants: miRNAs that are present exclusively in the moss *P. patens* (e.g. miR538, miR894, miR904, and miR1030), the spike-moss *S. moellendorffii* (e.g. miR1097), monocots (e.g. miR435, miR528, and miR812), and other individual species representing vascular plants (see Table S2). This number is higher than the number of miRNA families that are recognized in the best studied model plant, *A. thaliana* (193) (Kozomara & Griffiths-Jones, 2014). The Venn diagram showing the distribution of miRNA families among the taxonomic groups (see Fig.1) highlights 11 miRNA families that are present in plant species representing all other Viridiplantae taxa excluding Chlorophyta. Cuperus *et al*. (2011) reported eight miRNA families that were present in the common ancestor of all embryophytes (miR159/miR319 were classified as one family). Our studies confirm the presence of these eight (nine considering miR159 and miR319 separately) old miRNA families in the liverwort *P. endiviifolia*. Additionally, we were able to add two ancient miRNA families that were present in *P. endiviifolia* and other land plants. We added miR396, which is present in plant species representing all vascular plants but has not been identified in *P. patens*, and miR535, which was identified in *P. patens* and other embryophytes (Table1).

Surprisingly, we discovered the presence of three miRNA families that are common exclusively to liverworts and Chlorophyta (Table3). This result is unexpected because, until now, algal and land plant microtranscriptomes were considered to represent distinct domains. However, different experimental data suggest that the ancestor of all land plants was closely related to charophycean green algae similar to extant Charophytes. Assuming that liverworts are the most basal lineage of land plants, it is reasonable and evolutionarily attractive to find common miRNAs in the Chlorophyta species and liverwort representative *P. endiviifolia*. Because the liverworts from the genus *Pellia* that were collected in the field contain endophytic Glomeromycota fungi and algae (Ligrone *et al*., 2007), we set up an *in vitro* culture of *P. endiviifolia* male and female thalli (Fiedorow *et al*., 2001) that was alga- and fungus-free. Therefore, we further considered only those liverwort miRNAs exhibiting a high similarity to algal miRNAs or to algal sRNAs that were present in the sRNA deep-sequencing results when they were obtained from the RNA that was isolated from *P. endiviifolia* thalli that were grown *in vitro* and that produced a hybridization signal when analyzed by the northern technique. In addition, examining the *P. patens* and *S. moellendorffii* miRBase data for the presence of homologous chlorophycean sRNAs gave no positive outcome. Our results strongly indicate that *P. endiviifolia*, a liverwort representative, shares common molecular traits that might be exclusive for liverworts and algae. However, more extensive molecular studies on the microtranscriptome of other liverwort species need to be performed to support our hypothesis.

Our analyses also permitted the identification of 42 novel miRNAs belonging to 34 miRNA families that are at least *P. endiviifolia*- or liverwort-specific and are not present in *P. patens* or other land plants. Additionally, for 15 of the 42 novel miRNAs, we detected 17 primary transcripts. All of the new pri-miRNAs are able to fold into stem-loop structures containing miRNA and miRNA* in the stem region (see Fig. S6). Moreover, for all of the novel pri-miRNAs, we detected their cognate miRNA* in our sRNA sequencing results. For eight of the novel miRNAs, we identified their corresponding genes (see Table4). Pen-miR8185, for which we were able to identify both its gene and pri-miRNA, exhibits high similarity to one of the *C. reinhardtii* sRNAs. The fact that we identified pen-miR8185, which is homologous to one of the *C. reinhardtii* sRNA, as well as its precursor in the *P. endiviifolia* transcriptome data and its gene in the *P. endiviifolia* genome provides strong evidence of the presence of miRNAs that are homologous to algal miRNAs in liverworts.

We were only able to identify two *P. endiviifolia MIR* genes (*MIR408-5p* and *MIR536*) and nine primary transcripts encoding nine conservative land plant miRNAs for which miRNA* sequences were also found in our sRNA NGS data (see Table4; Fig. S6). Our results show that the length of the pri-miRNA fold-back fits into the standard range of plant pre-miRNA length and varies between 55 and 834 nt (Bologna *et al*., 2013). The minimum free energy (MFE) of the miR/miR* predicted structures varies between −9.4 and −365 kcal mol^−1^. The plant pre-miRNA fold-backs that were found in the miRBase display an average minimum free energy of −67.88 kcal (Ritchie *et al*., 2007; Kozomara & Griffiths-Jones, 2014). Thus, *P. endiviifolia* miRNA stem-loop structures show MFE values typical for other plant pre-miRNAs. The extended stem of the pre-miR stem-loop structure serves as a structural signal that is recognized by the plant pri-miRNA-processing machinery during base-to-loop processing (Mateos *et al*., 2010; Song *et al*., 2010; Cuperus *et al*., 2011; Bologna *et al*., 2013). Based on the predicted secondary structure of the *P. endiviifolia* pre-miR390/390a, we postulate a base-to-loop processing direction (see Fig. S6a). In angiosperms, this mechanism has also been demonstrated for pre-miR390a and pre-miR390b (Bologna *et al*., 2013). The same structural determinants of the pre-miRNA structure were found in the case of the novel pen-miR8166 precursor. We postulate that the precursor is also processed in the base-to-loop direction during miR8166 biogenesis. These results underline the similarity of liverwort miRNA precursors to those that have been identified in *P. patens* and vascular plants.

To date, all of the *MIR* genes that have been identified in *P. endiviifolia* represent independent transcriptional units. Four of 10 *MIR* genes contain one or two introns. This result agrees with available data for angiosperms, in which the majority of miRNAs are encoded by independent transcriptional units rather than being hosted in the introns of protein-coding or noncoding genes (Szarzynska *et al*., 2009; Kruszka *et al*., 2013, 2014). In all of the identified liverwort intron-containing *MIR* genes, miRNA-bearing fold-back structures were found in the second or third exon, which is an unusual feature compared with other embryophyte *MIR* gene structures. In *A. thaliana*, only two of the 23 known intron-containing *MIR* genes contain miRNA stem-loop structures that are located in the second or third exon, while in barley, of 13 known intron-containing *MIR* genes, only two have been found with an miRNA fold-back structure in exon 7 or exon 10 (Kurichara & Watanabe, 2004; Szarzynska *et al*., 2009; Jia & Rock, 2012; Sobkowiak *et al*., 2012; Kruszka *et al*., 2013, 2014). However, to draw further conclusions, additional *MIR* gene structures are required, and we cannot eliminate the possibility that other *P. endiviifolia MIR* intron-containing genes contain an miRNA fold-back structure in the first exon. In *P. patens*, approximately one-fourth of its known miRNAs are localized in close proximity to other *MIR* loci, suggesting that they are cotranscribed as polycistronic pri-miRNAs (Axtell *et al*., 2007). We did not find miRNA stem-loop structures in close proximity in the *P. endiviifolia* genomic sequences, which suggests a different organization of at least the liverwort *MIR* genes that were studied in this work.

Degradome analyses that searched for *P. endiviifolia* miRNA-guided cleaved targets identified only three mRNAs that are conserved across embryophytes (Table5). For many other conserved miRNAs, only new targets have been found (Tables5, S5). In our analysis, only experimentally verified targets were considered. Lenz *et al*. (2011) showed that plant cross-species-conserved miRNA-target pairs are mainly associated with developmental processes and transcriptional regulation. According to their results *P. endiviifolia* has miR160 and miR166 but not miR408 targets. For many other conservative miRNAs that target common transcription factor mRNAs in vascular plants, we found orthologous sequences in the *P. endiviifolia* transcriptome but were unable to identify miRNA-guided cleavage products within the degradome data. This result can be explained in two ways: there is no target conservation for conservative liverwort miRNAs, or conservative targets were expressed at very low levels in the studied liverwort tissue, thereby preventing the detection of cleaved products in the degradome data.

For 13 of the 42 novel miRNAs, the degradome data clearly confirmed the target predictions. Two of these miRNAs may represent transcription factors, while the others may represent different proteins of unknown function. Lenz *et al*. (2011) found that species-specific pairs (in *A. thaliana*) are involved in signal transduction and response to stress stimuli. Because the novel *P. endiviifolia* miRNAs were not detected in the moss *P. patens*, these miRNAs probably represent species-specific miRNAs; thus, their targets may be involved in the plant response to stresses.

Our data show that the liverwort microtranscriptome landscape is very similar to that of other land plants. Newly discovered miRNAs probably represent species-specific examples, but at least some of these miRNAs may represent liverwort-specific molecules. Along with the unexpected identification of miRNAs with high similarity to algal miRNA and sRNA sequences, these results confirm the location of *P. endiviifolia* at the root of the land plant evolutionary tree of life.

## Supporting Information

**Fig. S1**Schematic evolutionary tree of Viridiplantae with the evolutionary position of *Pellia endiviifolia* marked. **Fig. S2** Aniline blue staining for the presence of endophytic fungi in *Pellia endiviifolia* thallus cells. **Fig. S3** Detection of endophytic fungi and algae in *Pellia endiviifolia* thalli that were grown in the natural habitat and *in vitro* using specific PCR primers. **Fig. S4** Selected conservative miRNAs in the liverwort *Pellia endiviifolia* as detected by northern hybridization. **Fig. S5** Distribution of the read length in each novel miRNA cluster. **Fig. S6** Secondary structure predictions for the identified *Pellia endiviifolia* pre-miRNAs encoding conservative and novel miRNAs. **Table S1** Oligonucleotides that were used in the experiments **Table S3** Twenty-five pri-miRNAs that were identified in the *Pellia endiviifolia* transcriptome **Table S4** High expression of pen-miR8162-5p:pen-miR8162-3p in all of the sRNA NGS data sets **Table S5** Target mRNAs of known miRNAs that were identified in *Pellia endiviifolia* and confirmed by degradome data **Table S6** Targets for novel miRNAs that were identified in *Pellia endiviifolia* and confirmed by degradome data **Methods S1** Further methods used in the study.

Table S2 Best matches for the miRBase mature miRNAs that were found in *Pellia endiviifolia* sRNA-seq data
