# Supplementary material for: The liverwort Pellia endiviifolia shares microtranscriptomic traits that are common to green algae and land plants
Source: New Phytol. 2014 Dec 19;206(1):352–67. doi: 10.1111/nph.13220 (PMC4368373; doi:10.1111/nph.13220)
Supplement: Supplementary file 1 — Fig. S1Schematic evolutionary tree of Viridiplantae with the evolutionary position of Pellia endiviifolia marked. Fig. S2 Aniline blue staining for the presence of endophytic fungi in Pellia endiviifolia thallus cells. Fig. S3 Detection of endophytic fungi and algae in Pellia endiviifolia thalli that were grown in the natural habitat and in vitro using specific PCR primers. Fig. S4 Selected conservative miRNAs in the liverwort Pellia endiviifolia as detected by northern hybridization. Fig. S5 Distribution of the read length in each novel miRNA cluster. Fig. S6 Secondary structure predictions for the identified Pellia endiviifolia pre-miRNAs encoding conservative and novel miRNAs. Table S1 Oligonucleotides that were used in the experiments Table S3 Twenty-five pri-miRNAs that were identified in the Pellia endiviifolia transcriptome Table S4 High expression of pen-miR8162-5p:pen-miR8162-3p in all of the sRNA NGS data sets Table S5 Target mRNAs of known miRNAs that were identified in Pellia endiviifolia and confirmed by degradome data Table S6 Targets for novel miRNAs that were identified in Pellia endiviifolia and confirmed by degradome data Methods S1 Further methods used in the study. [file nph0206-0352-sd1.pdf]

New Phytologist **Supporting Information**

Article title:

Liverwort *Pellia endiviifolia* shares microtranscriptomic traits that are common to green algae and land plants

Authors:

Alaba S[2$], Piszczalka P[1$], Pietrykowska H[1], Sulkowska A[1,] Pacak AM[1], Sierocka I[1], Nuc PW[1], Singh K[1], Plewka P[1], Jarmolowski A[1], Karlowski WM[2]*, Szweykowska-Kulinska Z[1,2]*

[1]Department of Gene Expression, Institute of Molecular Biology and Biotechnology, Faculty of Biology, Adam Mickiewicz University, 61-614 Poznań, Poland

[2]Bioinformatics Laboratory, Institute of Molecular Biology and Biotechnology, Faculty of Biology, Adam Mickiewicz University, 61-614 Poznań, Poland

* Corresponding authors. [$]Both authors contributed equally to this work.

Supporting Information:

Methods S1

Fig. S1 Schematic evolutionary tree of Viridiplantae with marked evolutionary position of *P.endiviifolia*.

Fig. S2 Aniline blue staining for the presence of endophytic fungi in *P. endiviifolia* thalli cells.

Fig. S3 Detection of endophytic fungi and algae in *P. endiviifolia* thalli that were grown in the natural habitat and *in vitro* using specific PCR primers.

Fig. S4 Selected conservative miRNAs in the liverwort *P. endiviifolia* as detected by northern hybridization.

Fig. S5 Distribution of the read length in each novel miRNA cluster.

Fig. S6 Secondary structure predictions for the identified *P. endiviifolia* pre-miRNAs encoding conservative and novel miRNAs.

Table S1 Oligonucleotides that were used in the experiments.

Table S2 Best matches for the miRBase mature miRNAs that were found in *P. endiviifolia* sRNA-seq data (as separate Excel file).

Table S3 25 pri-miRNAs that were identified in the *P. endiviifolia* transcriptome.

Table S4 High expression of pen-miR8162-5p:pen-miR8162-3p in all of the sRNA NGS datasets.

Table S5 Target mRNAs of known miRNAs that were identified in *P. endiviifolia* and confirmed by degradome data.

Table S6 Targets for novel miRNAs that were identified in *P. endiviifolia* and confirmed by degradome data.

Methods S1

RNA and DNA isolation

Total RNA for sRNA detection was isolated using a method that permits the enrichment of sRNAs (Kruszka *et al.,* 2013) with the following modifications: the precipitation was performed with 1.2 vol. of ethanol and 0.4 vol. of salt solution (0.8 M sodium citrate, 1.2 M NaCl). For cDNA, total RNA was isolated similarly, and RNA was precipitated only after chloroform extraction using 0.5 vol. of isopropanol and 0.5 vol. of salt solution (0.8 M sodium citrate, 1.2 M NaCl). The quantity and quality of RNA were measured using a NanoDrop ND-1000 spectrophotometer (NanoDrop Technologies, Wilmington, DE, USA), while the RNA quality was estimated using agarose gel electrophoresis.

Genomic DNA (gDNA) was isolated from the liverwort thalli that were grown *in vitro* using the DNeasy Plant Maxi Kit (Qiagen, Hilden, Germany). The concentration and quality of genomic DNA were estimated using a NanoDrop ND-1000 spectrophotometer and confirmed by electrophoresis on a 0.6% agarose/EtBr gel.

pri-miRNA RACE experiments and genome walking

PCR and RT-PCR reactions, 5' and 3' RACE experiments for the identification of pri-miRNA structures and 5' and 3' genome walking analyses for the identification of *MIR* gene structures were performed as previously described (Sierocka *et al.,* 2011), with the exception of using Advantage® 2 Polymerase (Clontech Laboratories, Inc., Mountain View, CA, USA). The primer sequences that were used in the RACE and genome walking experiments and the primers that were used for the *MIR* gene amplification are shown in Table S1. The PCR products were separated on 1.2% agarose gels in 1× TBE buffer. The *MIR* gene structures were obtained by alignment of transcript and genomic sequences derived from RACE and genome walking experiment for each presented gene.

Deep-sequencing and bioinformatic analyses

For the deep-sequencing analysis, RNA isolation was performed from different types of *P. endiviifolia* thalli as described in the Materials & Methods. Total RNA (10 µg) was mixed 1:1 (vol/vol) with loading buffer II (Ambion® Austin, TX, USA), denatured for 2 min at 90°C and size-fractionated by 15% denaturing polyacrylamide gel electrophoresis; then, the small RNA fragments of 15–30 nt were isolated from the gel and purified. To localize the small RNAs, a 10-

bp DNA ladder was used. An aliquot of 1 μl of GlycoBlue Coprecipitant (15 mg/μl, Ambion® Austin, TX, USA) per sample was applied. The small RNA molecules were then ligated to a 5' adaptor and a 3' adaptor (Table S1) and converted to cDNA by RT-PCR following the protocol of Pant *et al.* (2009). The purified cDNA libraries were sequenced on the Illumina HiScanSQ platform using SR flow cell v1.5 (Illumina, Inc., San Diego, CA, USA). Each library was loaded onto a single lane in a flow cell. The adaptor sequences were identified and trimmed from each read using a customized Perl script. Reads in which the adaptor could not be identified were discarded. In this manner, low-quality reads were automatically rejected because no adapter sequence could be reliably identified. The sequencing reactions resulted in more than 14 million unique, quality-filtered and adaptor-trimmed reads. As expected for the sRNA sequencing procedure, the size distribution of the short sequences revealed the presence of a dominating class of 21-nt-long reads. Next, the BLASTn program was used to align the reads from each library to known plant mature miRNA sequences that permitted up to two mismatches to currently known plant miRNAs (Altschul *et al.,* 1997). The raw counts of reads for each library were recalculated into "reads per million" (RPM) using the total number of sequences matching miRNA. The 'mean count' for each sRNA sequence was calculated as the total number of normalized RPM counts from all of the libraries divided by the library number.

Degradome sequencing

Approximately 200 μg of total RNA was used for mRNA purification and degradome library construction. However, only female and male thalli growing *in vitro* and having no reproductive organs were used for total RNA isolation. Polyadenylated RNA was twice purified by hybridization with biotinylated oligo-dT 20-mers that were attached to streptavidin-coated magnetic beads (Dynal M-280) according to the manufacturer's instructions. The next steps of the degradome library preparation were performed as previously described (Addo-Quaye *et al.,* 2009; German *et al.,* 2009). Briefly, mRNA was ligated to an RNA oligonucleotide adaptor containing a 3' *Eco*P15I recognition site. The ligation products were used to generate first-strand cDNA by reverse transcription (RT). Then, a short PCR was used to amplify the cDNA to obtain sufficient quantities of DNA products. After digestion with *Eco*P15I, the 5'-ends of amplified ds-cDNA of 65-66 bp were ligated to a double-stranded DNA adaptor. The PAGE-purified ligation products were amplified using bar-coded primers to obtain final libraries that were compatible with the Illumina TruSeq system.

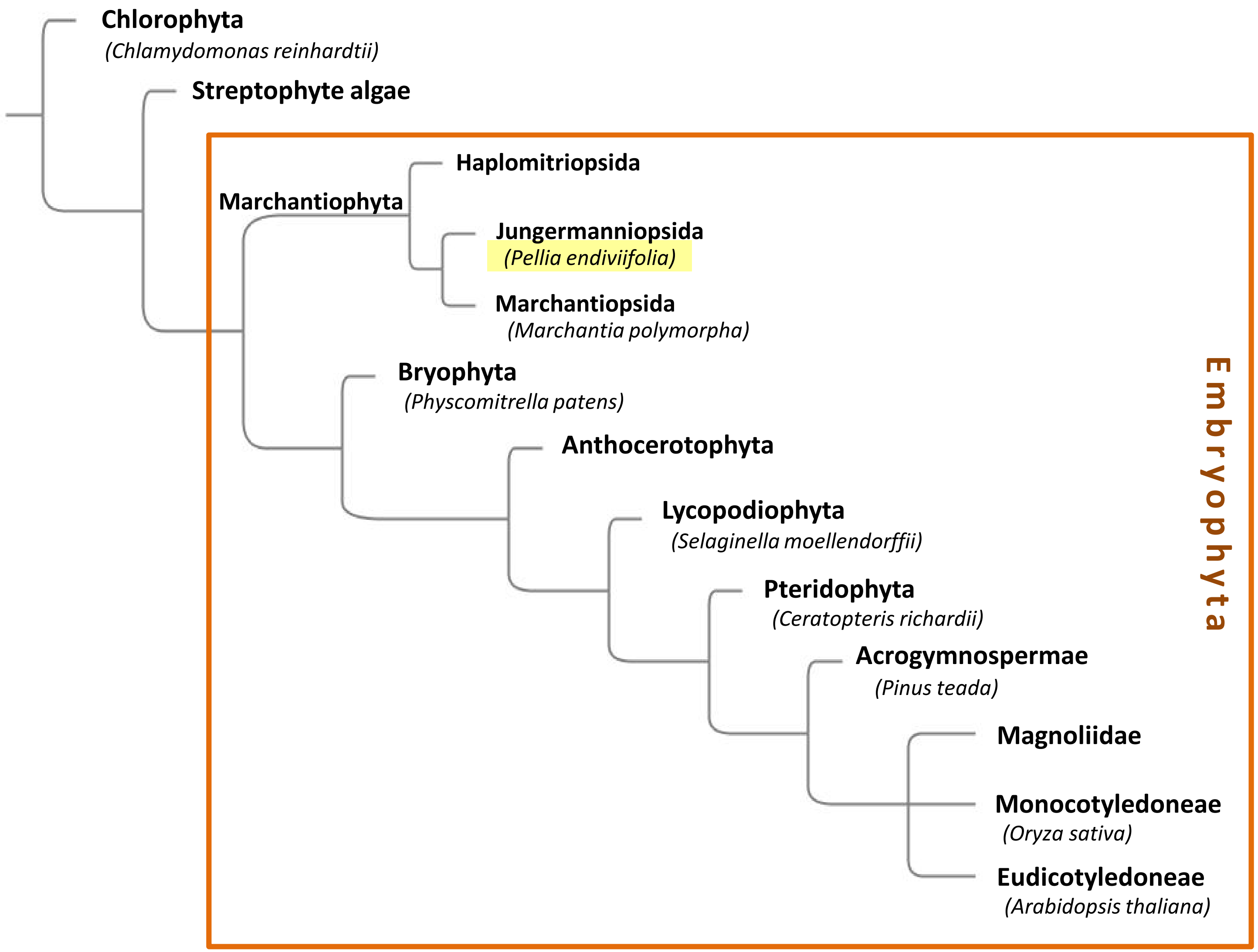

Fig. S1 Schematic evolutionary tree of Viridiplantae with marked evolutionary position of *P.endiviifolia*. A simplified summary of the phylogenetic relationships among the major lineages of the Viridiplantae based on the DNA sequence comparison of 360 plastid genomes, after Ruhfel *et al.* 2014. Model plant species in the main branches are shown.

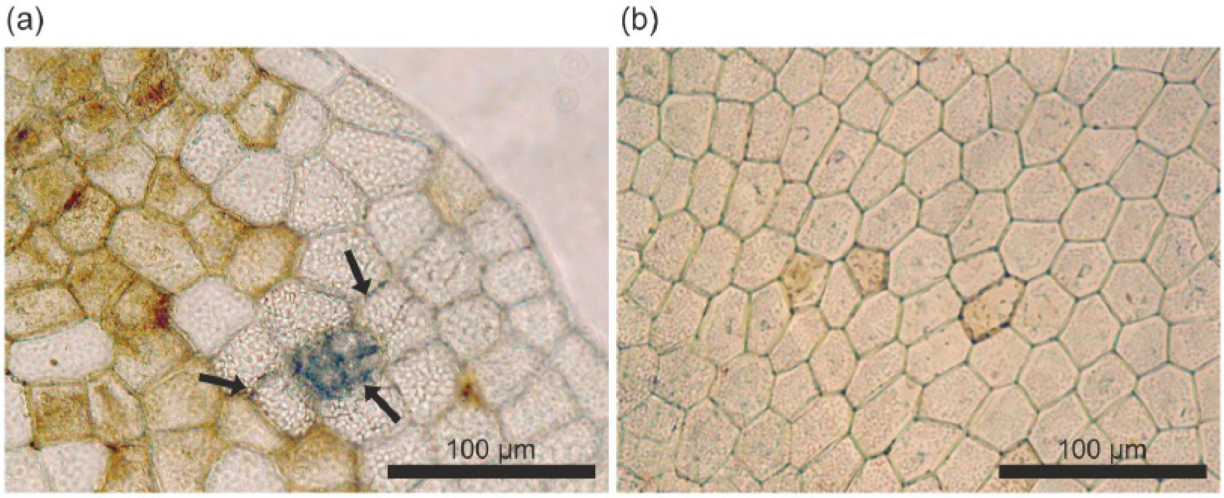

Fig. S2 Aniline blue staining for the presence of endophytic fungi in *P. endiviifolia* thalli cells. (a) Liverworts that were collected in the natural habitat and (b) liverworts that were grown *in vitro.* The arrows indicate fungal hyphae (Zeiss Axioskop 2 plus that was equipped with a Power Shot G5 Canon Digital Camera, 130×).

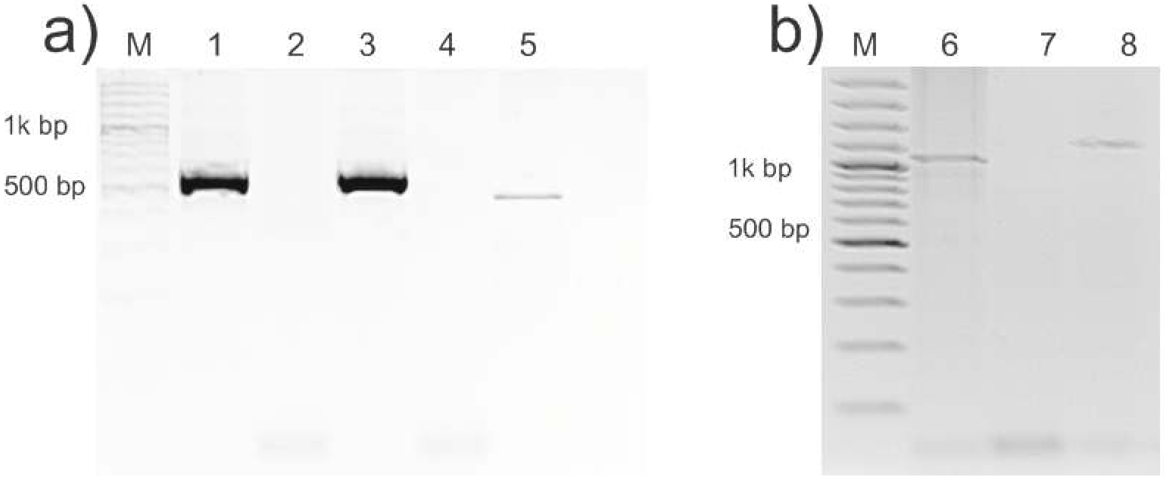

Fig. S3 Detection of endophytic fungi and algae in *P. endiviifolia* thalli that were grown in the natural habitat and *in vitro* using specific PCR primers. (a) Agarose gel electrophoresis of PCR products using DNA that was isolated from *P. endiviifolia* that was grown *in vitro* (lanes 1-4) and *in vivo* (lane 5). Lanes 1 and 3 represent the PCR products that were obtained using primers for 18S rDNA, which is universal for plants and fungi, while lanes 2, 4, and 5 show the PCR products that were obtained using another pair of primers that are specific for fungi 18S rDNA. (b) Agarose gel electrophoresis of the PCR products using DNA that was isolated from *P. endiviifolia* that was grown *in vitro* (lane 7) and *in vivo* (line 8), and using DNA that was isolated from *C. reinhardtii* (lane 6). All PCR reactions were performed using the same pair of primers that are specific for *Chlorophyta*. M – 100 bp DNA ladder (Fermentas by Thermo Fisher Scientific Inc., Waltham, MA USA).

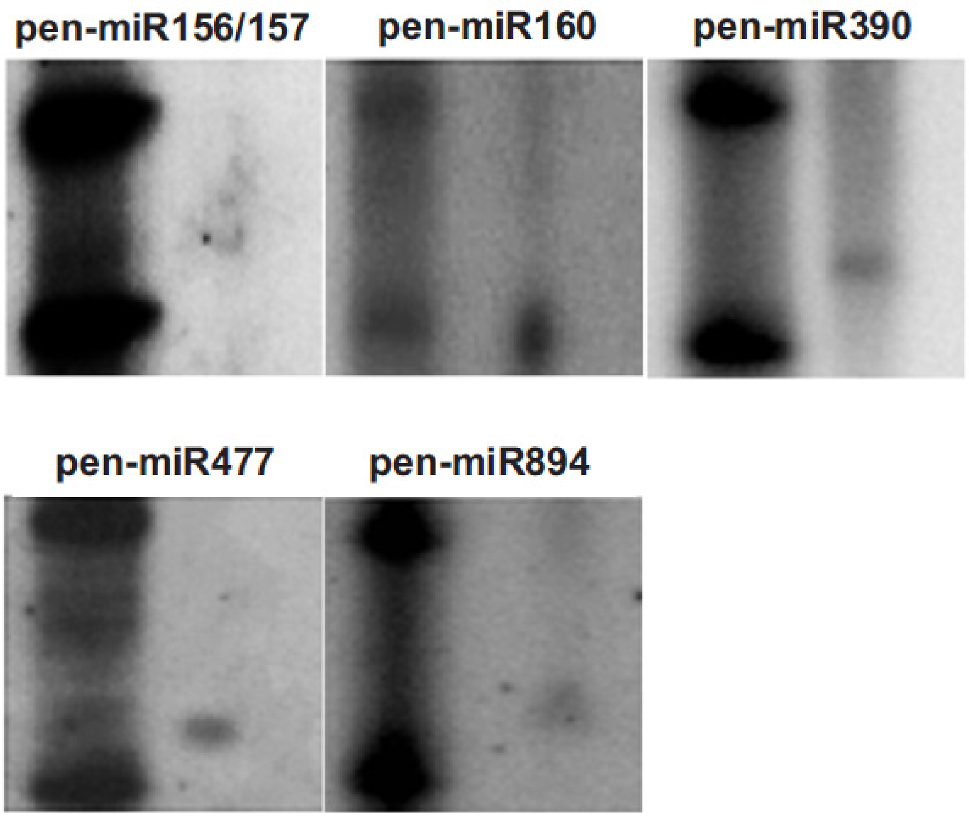

Fig. S4 Selected conservative miRNAs in the liverwort *P. endiviifolia* as detected by northern hybridization. Left side of the northern blots – RNA Decade molecular size marker including bands that are 30 nt and 20 nt long. Above the blots, the name of each miRNA is provided.

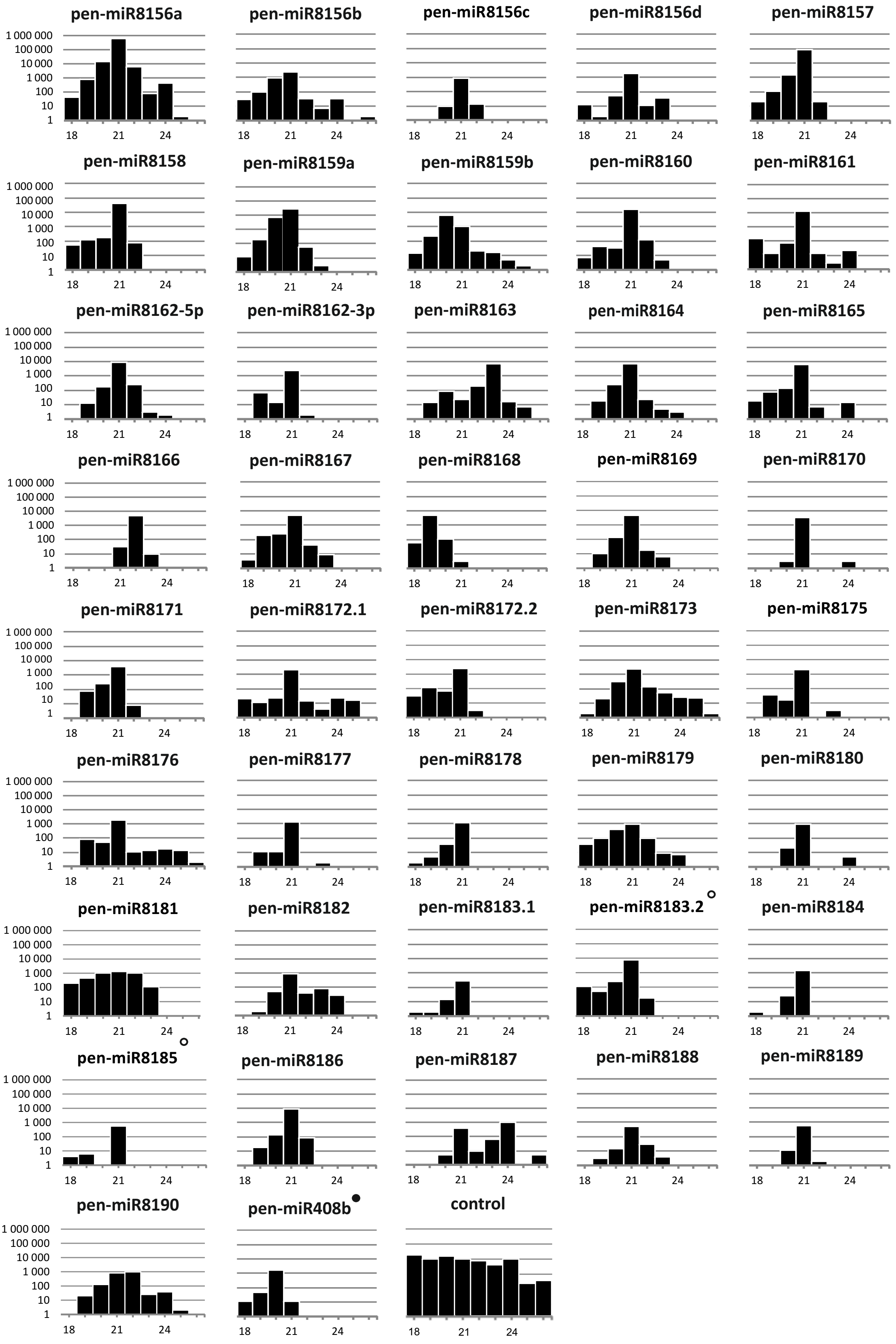

pen-miR8156a
pen-miR8156b
pen-miR8156c
pen-miR8156d
pen-miR8157
pen-miR8158
pen-miR8159a
pen-miR8159b
pen-miR8160
pen-miR8161
pen-miR8162-5p
pen-miR8162-3p
pen-miR8163
pen-miR8164
pen-miR8165
pen-miR8166
pen-miR8167
pen-miR8168
pen-miR8169
pen-miR8170
pen-miR8171
pen-miR8172.1
pen-miR8172.2
pen-miR8173
pen-miR8175
pen-miR8176
pen-miR8177
pen-miR8178
pen-miR8179
pen-miR8180
pen-miR8181
pen-miR8182
pen-miR8183.1
pen-miR8183.2
pen-miR8184
pen-miR8185
pen-miR8186
pen-miR8187
pen-miR8188
pen-miR8189
pen-miR8190
pen-miR408b
control
1 000 000
100 000
10 000
1 000
100
10
1
18
21
24

Fig. S5 Distribution of the read length in each novel miRNA cluster.

OY axis - mean counts; OX axis - the length of a given RNA fragment in each cluster (nt). In each cluster, there are dominating RNA species in a length range that is typical for miRNAs. The very last diagram shows the distribution length for a small RNA cluster in which no particular RNA fragment was dominant (control). ○ -sRNA homologs that were found in *C. reinhardtii* NGS data with 1 or 2 mismatches in the overlapping regions. ● depicts pen-miR408b*. miR408b* was originally identified as a novel miRNA and was then identified as an miR* in the pre-miRNA408. The read counts represent a single NGS experiment.

Fig. S6

Secondary structure predictions for the identified *P. endiviifolia* pre-miRNAs encoding conservative and novel miRNAs.

- miRNA (highly/equally abundant sRNA sequence perfectly aligned to the precursor, identified in NGS data and confirmed by northern hybridization)
- alternative sRNA (identified in NGS data and - in case of novel miRNA - confirmed by northern hybridization)
- miRNA* (significantly less abundant sRNA sequence perfectly matching to the precursor and identified in NGS data)
- indicates precursor with experimentally confirmed primary transcript gene

(a) conservative miRNAs

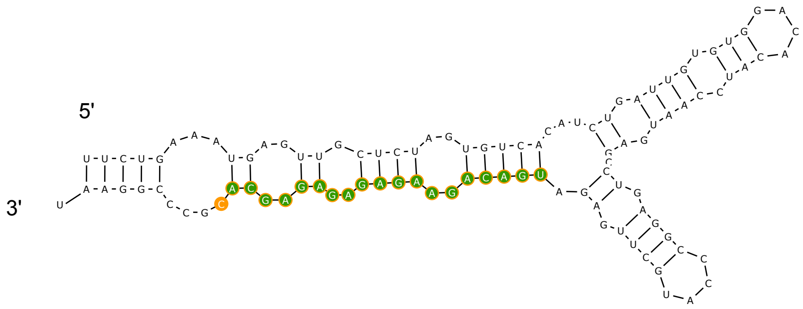

pen-miR156i/156j

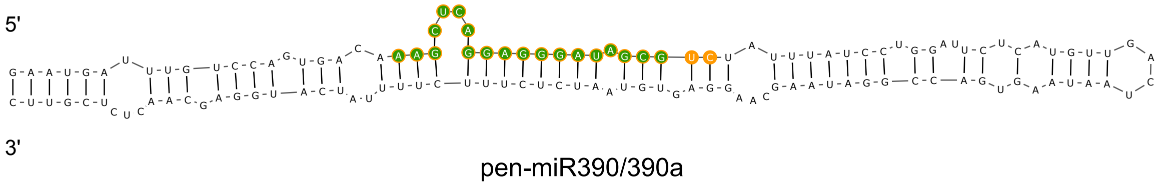

pen-miR390/390a

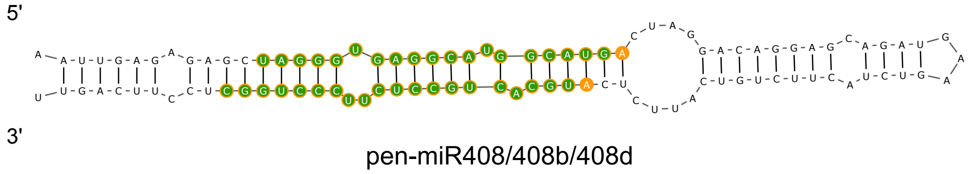

pen-miR408/408b/408d

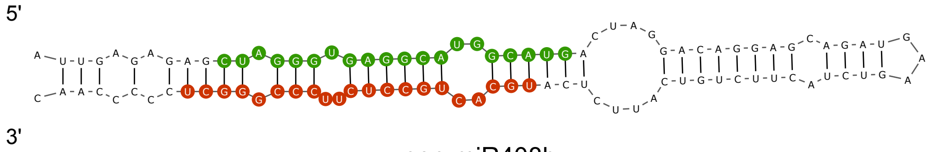

pen-miR408b

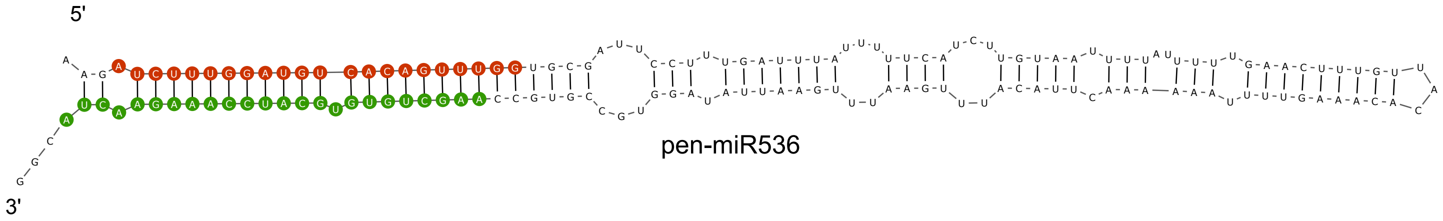

pen-miR536

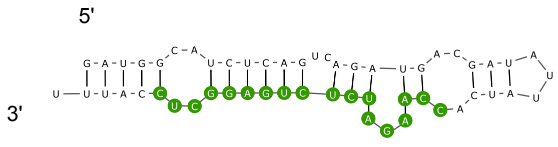

pen-miR7732

(b) novel miRNAs

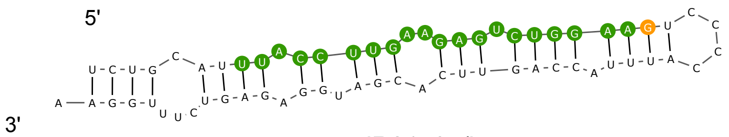

pen-miR8159a/b

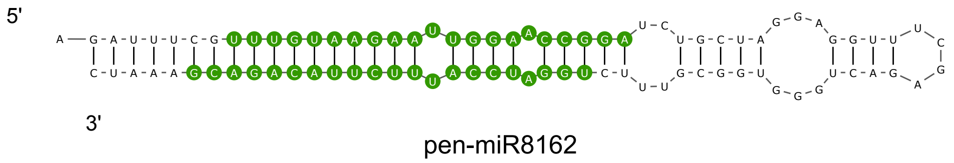

pen-miR8162

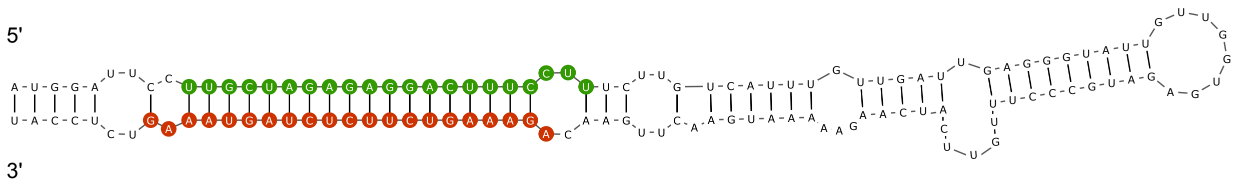

pen-miR8165

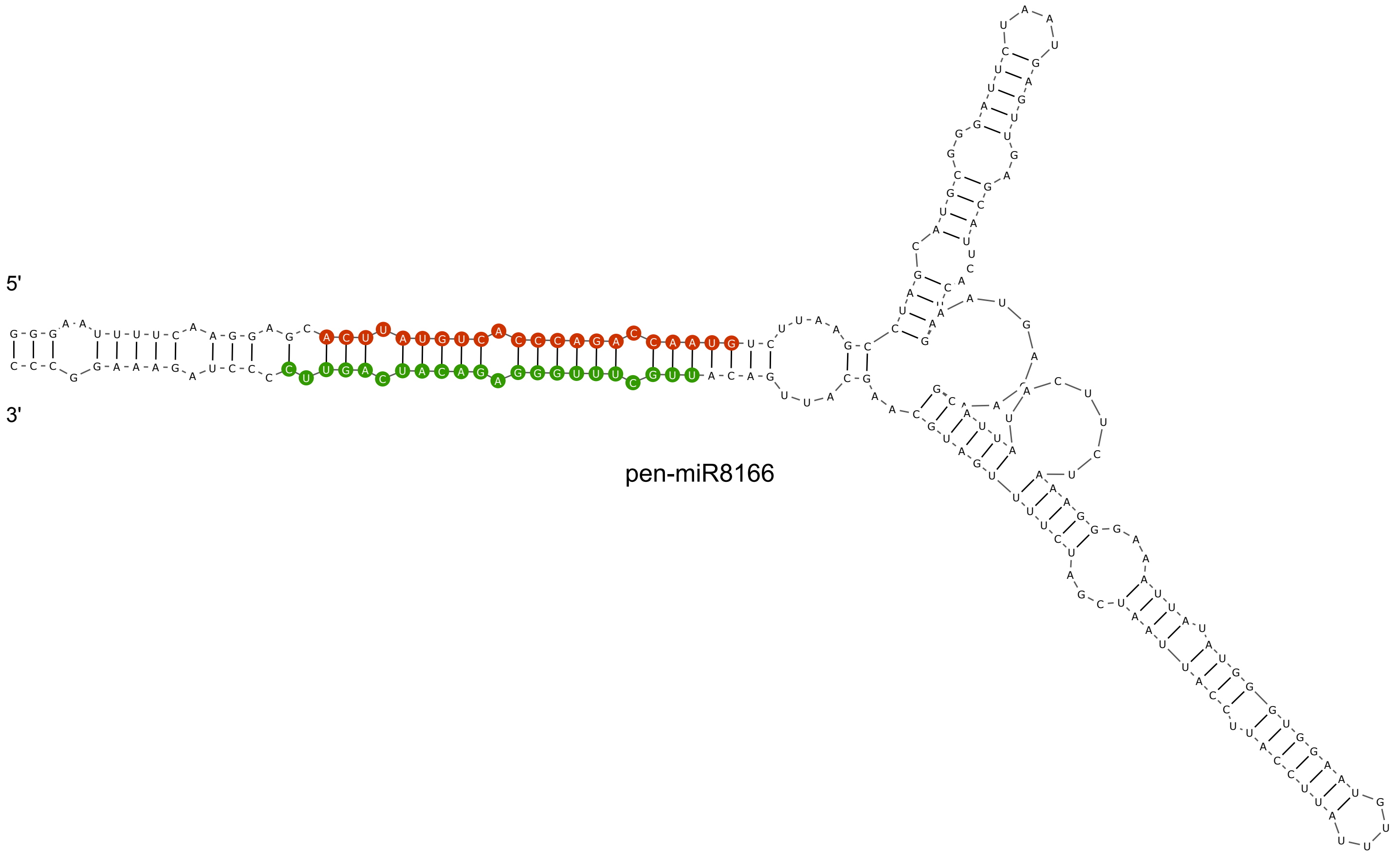

pen-miR8166

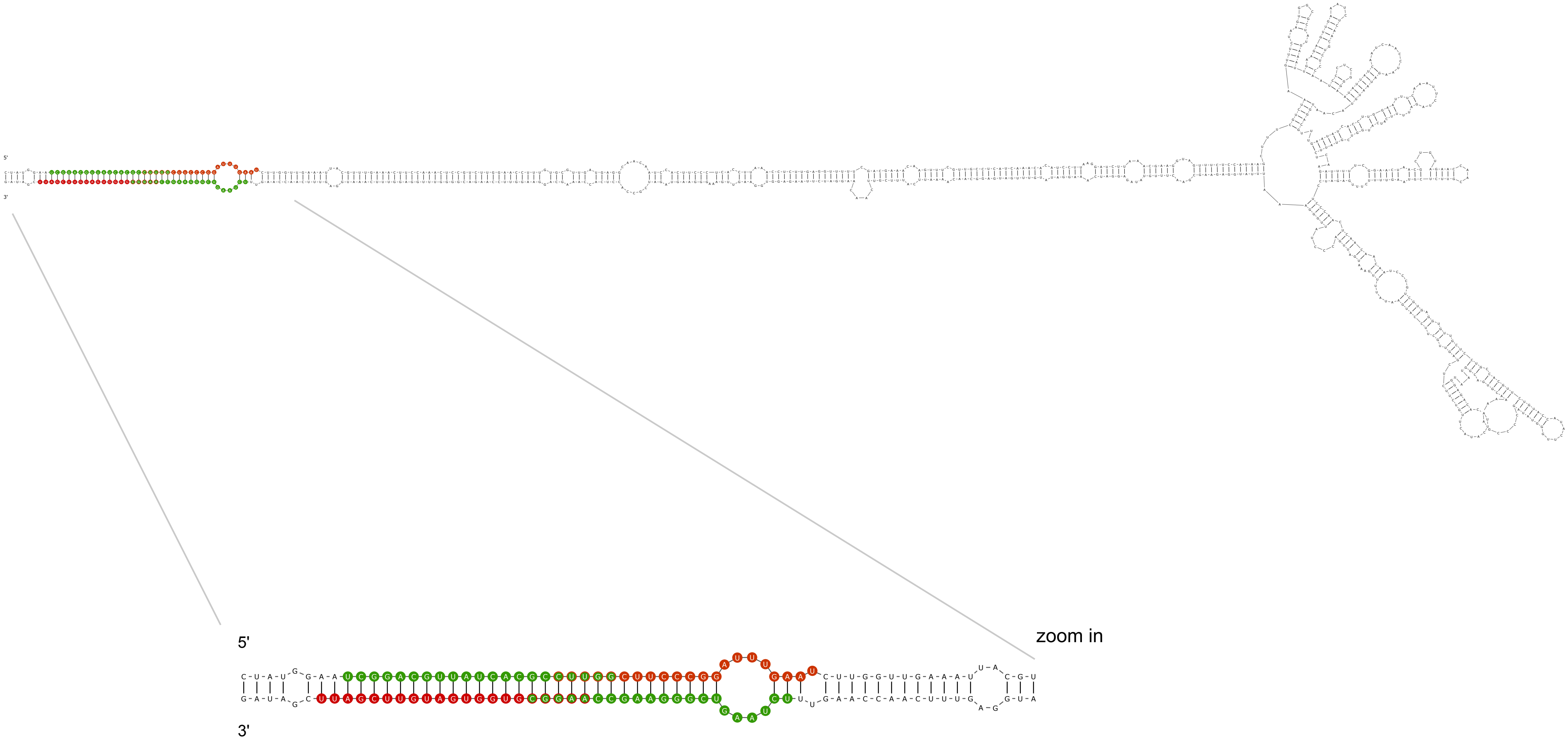

pen-miR8172

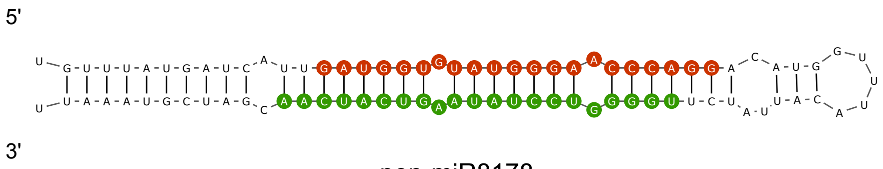

pen-miR8178

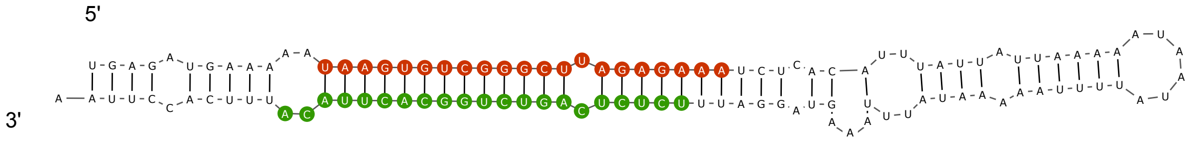

pen-miR8182

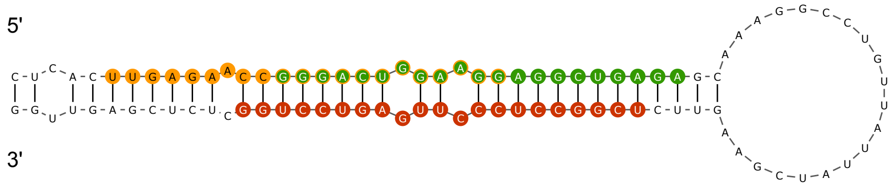

pen-miR8183

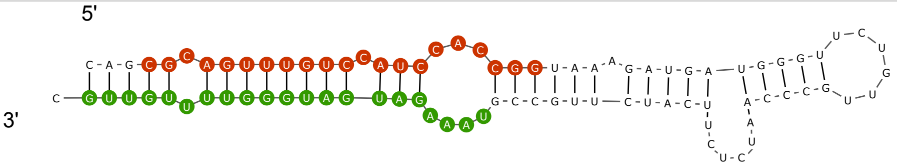

pen-miR8185

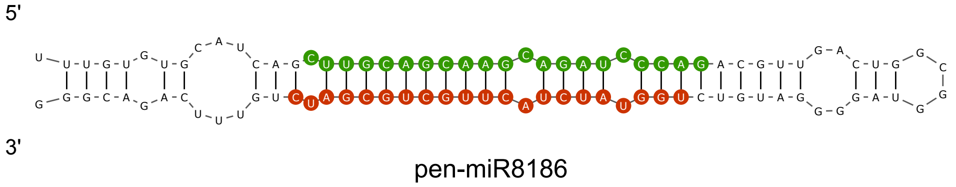

pen-miR8186

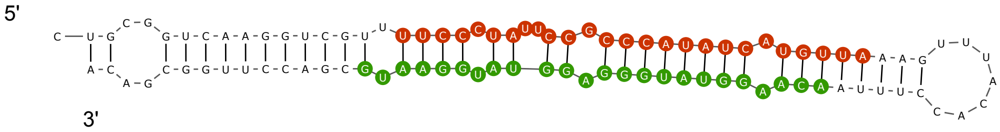

pen-miR8187

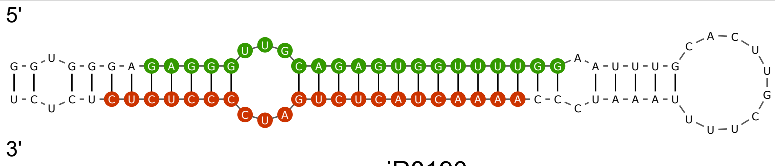

pen-miR8190

**Table S1 Oligonucleotides that were used in the experiments.**		
No.	Name	Sequence (5`→3`)
primers specific for the amplification of the *rps11 – rpl2* plastid gene cluster across Chlorophyta species (Provan *et al.,* 2004)		
1	UCP5	acttchggtgchaghgawataatt*
2		gaaachcgdatgggdtckgg*
* after IUPAC nucleotide code: h (A or C or T), w (A or T)		
primers for the amplification of a portion of the small subunit rRNA specific for arbuscular endomycorrhizal- fungi (Simon *et al.,* 1992)		
3	VANS1	gtctagtataatcgttatacagg
4	NS2	ggctgctggcaccagacttgc
universal primers for the amplification of a portion of the eukaryotic small subunit rRNA (*Simon et al.,* 1992)		
5	NS1	gtagtcatatgcttgtctc
4	NS2	ggctgctggcaccagacttgc
Oligonucleotides used for small RNA library preparation		
6	5' RNA adaptor	guucagaguucuacaguccgacgauc
7	3' RNA adaptor	5Phos/ -ucguaugccgucuucugcuugu/3SpC3/
novel miRNA hybridization probes		
8	Control_probe	acgccctacaaccccaaggag
9	cre-miR1444b	tgcctgcctccaccctac
10	pen-miR8156a	tcctagacgatgtgtgggaga
11	pen-miR8156b	gcctagacgatgtgtgggaga
12	pen-miR8156d	tcctagacgatgtgtgggcga
13	pen-miR8157	tcatcgacgatgtctgggaaa
14	pen-miR8158	tcgagcagaatgcgtccttca
15	pen-miR8159a	cttccagactcttcaaggtaa
16	pen-miR8159b	ttccagactcttcaaggtaa
17	pen-miR8159b	tattgcccgagtctttaggga
18	pen-miR8161	ggctccacctctctgctcgaa
19	pen-miR8162-3p	cgtctgtaagaaatggatcca
20	pen-miR8162-5p	tccggttccaattcttacaaa
21	pen-miR8163	tgagattgttttcttccacgggg
22	pen-miR8164	ggggaagtttgccacaaattg
23	pen-miR8165	aaggaaagtcctctctagcaa
24	pen-miR8165	gaactgatgtctcccaaagcaa
25	pen-miR8167	gattgggtatagtaggcatgg
26	pen-miR8168	ccgattgagctacgtcccc

27	pen-miR8169	agctcaacccttcttatcgca
28	pen-miR8170	atgcatctcctctgtgaagca
29	pen-miR8171	ctcagtcccatgcttctattg
30	pen-miR8172.1	ccaaggcgtgataacgtccga
31	pen-miR8172.2	gccttggcttcccgacttaga
32	pen-miR8173	tacgatcacatctcctactgg
33	pen-miR8175	gattggatatagcaggcatgg
34	pen-miR8176	tttgaggtcctgaaacacaga
35	pen-miR8177	catgccctgcagtccatcaga
36	pen-miR8178	gttccctacataatccggtag
37	pen-miR8178	agttatgcctactgtatccca
38	pen-miR8180	gacgacgagtgcaagtctgta
39	pen-miR8181	ggtccatccgaccgcacgggc
40	pen-miR8182	tgtaagtgccagactgagaga
41	pen-miR8183.1	gcctccttccagtcccggttc
42	pen-miR8183.2	tctcagcctccttccagtccc
43	pen-miR8184	gtcacaatgtctcttcctcca
44	pen-miR8185	caacaaaacccatcatcttta
45	pen-miR8186	ctgggatctgcttgctgcaag
46	pen-miR8187	cattccatacctcccataccttgt
47	pen-miR8188	tggttccgttgcagcgtttct
48	pen-miR8189	acaaccctgtactatcctcct
conserved miRNA hybridization probes		
49	aly-miR157a	gtgctctctatcttctgtcaa
50	ppt-miR160a	tggcatacagggagccaggca
51	ppt-miR166a	ggggaatgaagcctggtccga
52	sbi-miR168	gtcccgatctgcaccaagcga
53	ppt-miR319d	gggagctcccttcagtccaag
54	ppt-miR390a	ggcgctatccctcctgagctt
55	ppt-miR408b	agccagggaagaggcagtgca
56	ppt-miR477a-5p	tggaagcctttgagggagag
57	ppt-miR894	ggtgaacccgacgtgaaacg
RACE and Genome walking experiments		
58	miR8165_3p_clearup_F	tggtttgcatggttaaaaagtg
59	miR8165_3p_clearup_GW2	ccactcatggattttgacatttgtatcacc
60	miR8165_3p_clearup_R	cgagatttcaagagtattaatattaatgca
61	miR8165_5p_clearup_brut_F	atgggggatcattctttgtg
62	miR8165_5p_clearup_brut_R	cgctaggccactgaaggtc
63	miR8165_5p_clearup_GW	ggaatcgactgaatccaaaccgaagtt
64	miR8165_gen_For	acgcttcacttgatcgtgttttgtgta
65	miR8165_gen_Rev	gtcatacacaaaacacgatcaagtgaagc

66	408_5p_clearup_F	atgggagggaacagacactg
67	408_5p_clearup_R	gaggcagtgcatgagaatga
68	408_gen_5p_For	gtaagaattggaaccggatctgctagg
69	408_gen_5p_Rev	cctagcagatccggttccaattcttac
70	408bGSP1(b)	catgatcagctcaccctcaagggaaat
71	408bGSP2(b)	gaggcaatgcatgccagcgtgag
72	408bNGSP1(b)	ggatcctccagtccagacatccttgc
73	408bNGSP2(b)	cgcgtctaaggctgattaagggtggtt
74	miR8187_3p_clearup_F	ggaggtggatgattgacgta
75	miR8187_3p_clearup_R	tgtatattagaagccaaatgtgtca
76	536_3p_clearup_ F	attgtagtggctgacgataggagaag
77	536_3p_clearup_R	taaagaatgaaacctgggatgtaaa
78	536_5p_clearup_GW1	ttcaactctgaatcacctttctcgagttc
79	536_5p_clearup_GW2	tccccatcaataatcactagttggtttca
80	Gen_miR8187_5p1_Marta	ccgtgttctgtattcatgataccacatgc
81	Gen_miR8187_5p2_Marta	catacgccttgactgcatcgagataactt
82	Gen_miR8178_3p_Basia	atacgttacgtgaacttactggtcc
83	Gen_miR8178_5p_Basia	gacgtcatgaagatgttctgtattg
84	Gen_miR408_3p1_Kara	cctgcatgaaaagcacagtacagaaggat
85	Gen_miR408_3p2_Kara	gcatgactaggacaggagcagatgaagtc
86	Gen_miR408_5p1_Kara	tctttcccacgtatatccttctgtactgtgc
87	Gen_miR408_5p2_Kara	gcagctttggtatatccaggtcatgtgtc
88	GW_408_NGSF	gggcaaaccataagggtgaacttatcat
89	GW_408_GSF	atgttgggtacggagttgctaaggagta
90	GW_408_GSR	aagaggcagtgcacaccaatgaataat
91	GW_408_NGSR	tttccggaacaatatgaagggacgtat
92	MIR8183.1_5p_clearup_GW1	gcgagaggtaattgaagaggtaattggaa
93	MIR8183.1_5p_clearup_GW1	gcgagaggtaattgaagaggtaattggaa
94	MIR8183.1_5p_clearup_GW2	atacacgtaggaatggaaagcgagtatcc
95	MIR8183.1_5p_clearup_GW2	atacacgtaggaatggaaagcgagtatcc
96	MIR8183.1_gen_3p_For	cgtggatcgtggtatgcattgttgt
97	MIR8183.1_gen_3p_Rev	ggaggccgagaacttcgataataacag
98	MIR8183.1_gen_5p_For	tcatttttcttgatgaacaaagggcatc
99	MIR8183.1_gen_5p_Rev	caccaacaataccctcaatcaacaaatgac
100	MIR8186_gen_3p_For	gcggtaggggatgtctggtatctactt
101	MIR8186_gen_3p_Rev	caagtagataccagacatcccctaccg
102	MIR8186_gen_5p_For	acgcttcacttgatcgtgttttgtgta
103	MIR8186_gen_5p_Rev	gtcatacacaaaacacgatcaagtgaagc
104	MIR8190_3p_NS_GW1	atcggtgttattgctgattatttcccatt
105	MIR8190_3p_NS_GW1	atcggtgttattgctgattatttcccatt
106	MIR8190_3p_NS_GW2	aacctgtaatgaaggccccggagtaagta
107	MIR8190_3p_NS_GW2	aacctgtaatgaaggccccggagtaagta
108	MIR8185_3p_GW1_new	catttgacattgcctttccagtggtat

109	MIR8185_3p_GW2_new	tttgaaccctctgagctgttctctcac
110	MIR8185_5p_GW1_new	ataccactggaaaggcaatgtcaaatg
111	MIR8185_5p_GW2_new	agtaggaagcatagtccgtcaccaaca
112	SL_miR8165_39420_1	gtggagttctgcacggaaagacgatag
113	SL_miR8165_39420_2	tctacatcacgcggacttgattctgg
114	SL_miR8165_7756_1	actgaggtgggagttcttggaaggcta
115	SL_miR8165_7756_2	gtctgtgcttctgtgctggcatgtagt
116	TR_408_10_4_3p_b	tgcaagaatgtaccatgtgtaagaacaggtg
117	TR_408_5p_b	gacttcatctgctcctgtcctagtcatgc
118	TR_408_8_1_3p_b1	gctcccccaacatatatggaaaccagt
119	TR_408_8_1_3p_b2	ccaacgaagttttatttggtggatgtg
120	TR_408_8_2_3p_b	ctcttccctggctccttcagtttctca
121	TR_408a	catgactaggacaggagcagatgaagtct
122	TR_408b	cctggctccttcagtttctcattcatatag
123	TR_408c	aatgagaaactgaaggagccagggaag
124	TR_408d	gcagtgcatgagaatgacagaagtagac
125	TR_536_5p_b	aaggaatcgcaccaaactgtgacatcc
126	TR_536a	cacagtttggtgcgattcctttgattt
127	TR_536b	ggtgcgattcctttgatttatttttcatc
128	TR_536c	ggcacggcacctataattcatattcaaa
129	TR_536d	aataaatcaaaggaatcgcaccaaactgt
130	TR_MIR8165_5p_b1	aatcgaacttgcctactacgcgaagaaaa
131	TR_MIR8165_5p_b2	catgtactctgcgttgataccactgcttg
132	TR_MIR8165a	gttgattgagggtattgttggtgagatg
133	TR_MIR8165b	ccctttgttcatcaagaaaaatgaacttg
134	TR_MIR8165c	tcatttttcttgatgaacaaagggcatc
135	TR_MIR8165d	caccaacaataccctcaatcaacaaatgac
136	TR_MIR8187a	cgtggatcgtggtatgcattgttgt
137	TR_MIR8187b	acattctgcggtcaaggtcgttttc
138	TR_MIR8187c	taacatgatatgggcggaatagggaaaac
139	TR_MIR8187d	gaatgtgacaacaatgcataccacgat
140	TR_MIR8178_3p_b1	agaggaccaatgtaggcaattttccaaac
141	TR_MIR8178_3p_b2	gaccaatgtaggcaattttccaaacgatag
142	TR_MIR8178a	gggaacccaggacatggtttacattatct
143	TR_MIR8178b	cgttgatgacttataggaccccaagataa
144	TR_MIR8178c	tcatgtttatgatcattgatggtgtatgg
145	TR_MIR8178d	cgttgatgacttataggaccccaaga
146	TR_MIR408_3p_b	gcatgactaggacaggagcagatgaagtc
147	TR_MIR408_5p_b	acgcgtgccctatagtgagtcgtattaca
148	TR_MIR408a	tgaagtctacttctgtcattctcatgcac
149	TR_MIR408b	agaagtagacttcatctgctcctgtcctagt
150	TR_MIR408c	ggcatggcatgactaggacagg
151	TR_MIR408d	gaagaggcagtgcatgagaatgaca

152	TR_NL1	tgagagcaaaggcctgttattatcgaa
153	TR_ NL1_5p_b	attagccaactcgagagccaggactcaa
154	TR_ NL10	aagcaagtgcaaattccaaaaccac
155	TR_NL11	gtaagaattggaaccggatctgctagg
156	TR_ NL12	cctagcagatccggttccaattcttac
157	TR_ NL13	gttctgttgcccaatctcttcatcttg
158	TR_ NL14	caagatgaagagattgggcaacagaac
159	TR_NL2	ggaggccgagaacttcgataataacag
160	TR_NL3	acgcttcacttgatcgtgttttgtgta
161	TR_ NL3_5p_b	gtagataccagacatcccctaccgccagt
162	TR_NL4	gtcatacacaaaacacgatcaagtgaagc
163	TR_NL4_3p_b	tgccttggttcaaaaccctaaactactcg
164	TR_NL5	gcggtaggggatgtctggtatctactt
165	TR_NL6	caagtagataccagacatcccctaccg
166	TR_NL6_3p_b1	cgggatattgagaagttgagggatacgat
167	TR_NL6_3p _b2	ttatcatttatgcccaggacgtgttagcc
168	TR_MIR8185	cgagtgtagtttgccttggttcaaaac
169	TR_NL8	agggttttgaaccaaggcaaactacac
170	TR_NL9	ctctctccactgaagccactgagaa
171	TR_408a	catgactaggacaggagcagatgaagtct
172	TR_408b	cctggctccttcagtttctcattcatatag
173	TR_408c	aatgagaaactgaaggagccagggaag
174	TR_408d	gcagtgcatgagaatgacagaagtagac
175	miR8165_3p_clearup_R	cgagatttcaagagtattaatattaatgca
176	MIR8185_5`_RACE_fin1	gcatctgtgatggaaggtatttttactgct
177	MIR8185_5`_RACE_fin2	cactccttaaatcgaggctcatcaatgtg
Genomic PCR primers		
178	536_gen_3p_For	gttgattgagggtattgttggtgagatg
179	536_gen_3p_Rev	ccctttgttcatcaagaaaaatgaacttg
180	536_gen_5p_For	gttctgttgcccaatctcttcatcttg
181	536_gen_5p_Rev	caagatgaagagattgggcaacagaac
182	miR8178_gen_5p_For	ctctctccactgaagccactgagaa
183	miR8178_gen_5p_Rev	aagcaagtgcaaattccaaaaccac
184	miR8187_gen_3p_For	cgagtgtagtttgccttggttcaaaac
185	miR8187_gen_3p_Rev	agggttttgaaccaaggcaaactacac
186	miR8187_gen_5p_For	gcggtaggggatgtctggtatctactt
187	miR8187_gen_5p_Rev	caagtagataccagacatcccctaccg
188	FIN_408_5p_F	atgggagggaacagacactgaat
189	FIN_408_5p_R	acttcatctgctcctgtcctagtca
190	FIN_miR8187_3p_F	gtggtatgcattgttgtcacattct
191	FIN_miR8187_3p_R	tcccaacttggtccaacaaaag
192	FIN_miR8187_5p_F	atgggatccacttaccgttcct

193	FIN_miR8187_5p_R	actttgcctctcatagagatgtcgt
194	FIN_536_3p_F	tttcagtaagaagatctttggatgtca
195	FIN_536_3p_R	taaagaatgaaacctgggatgtaaa
196	FIN_536_5p_F	gacagtgcgtccatacgagagtg
197	FIN_536_5p_R	tgacatccaaagatcttcttactgaaa
198	FIN_miR8178_5p_F	ggaagttatgatcgctggagtgtct
199	FIN_miR8178_5p_R	gggaaatttaccacaccgtaacaaa
200	FIN_miR408_3p_F	cattccaatattcagaaaggacctg
201	FIN_miR408_3p_R	catggaatcaaaatgctcaatatattact
202	FIN_miR408_5p_F	gtgtgtttgaccaatgaaagcttga
203	FIN_miR408_5p_R	caattcattcatttctttcccacgta
204	FIN_MIR8183.1_3p_F	aggcctgttattatcgaagttctcg
205	FIN_MIR8183.1_3p_R	cgaccaaggcaaattatattaaacaca
206	FIN_MIR8186_3p_F	caagttatttcatggttttgtgtg
207	FIN_MIR8186_3p_R	ctgatgaaatccttgagtaattaattc
208	MIR8183.1_3p_clearup_ F	gagcaaaggcctgttattatcgaagttct
209	MIR8183.1_3p_clearup_R	cgaccaaggcaaattatattaaacacaggt
210	ath156a_F_csp	gagaaacgcaaagaaactgaca
211	ath156a_R_csp	gatcagcaccggaatctgac
212	ath156a_R_sp	tctgacagaaagagcagtgagc
213	ppt156a_F_csp	gtggaggatgtgggagtgac
214	ppt156a_R_csp	agaggtgcgacaggaagaga
215	ppt156a_R_sp	tgcgacaggaagagagtgagc
216	ath160a_F_csp	atgctgagcccatcgagtat
217	ath160a_R_csp	tatgcatggctcctcatacg
218	ath160a_R_sp	tatgcatggctcctcatacgc
219	ppt160a_F_csp	aactgcctggctccctgt
220	ppt160a_R_csp	gctatgcagctcgacaatca
221	ppt160a_R_sp	tctgtctggctccctggatgc
222	ath166a_F_csp	aggggctttctcttttgagg
223	ath166_R_csp	aatgaagcctggtccgaag
224	ath166_F_sp	ggactgttgtctggctcgagg
225	166_R_sp	ggggaatgaagcctggtccga
226	ppt166a_F_csp	gaagccatgtgccttttcat
227	ppt166a_R_csp	gcctggtccgaagctttaat
228	ppt166a_F_sp	ggaatgccccctggcccgaagc
229	sbi168_F_csp	ctcgcttggtgcagatcg
230	sbi168_R_csp	ggattcacttggtgcaagg
231	sbi168_R_sp	attcacttggtgcaaggcggg
232	ath168a_F_csp	ttggtttgtgagcagggatt
233	ath168a_R_csp	ggatccgattcagttgatgc
234	ath168a_F_sp	tcgcttggtgcaggtcgggaa
235	ath168a_R_sp	attcagttgatgcaaggcggg

236	Ppt319c_F_csp	ggagcttccttcggttcaat
237	Ppt319c_R_csp	gagctcccttcagtccaaga
238	Ppt319c_F_sp	gagcttccttcggttcaatag
239	Ppt319c_R_sp	gggagctcccttcagtccaag
240	Ath319a_F_csp	tgagtccattcacaggtcgt
241	Ath319a_R_csp	gctcccttcagtccaatcaa
242	Ath319a_F_sp	agagcttccttgagtccattc
243	Ath319a_R_sp	agggagctcccttcagtccaa
244	Ath408_F_csp	ggaacaagcagagcatggat
245	Ath408_R_csp	agcaataaaattccattgaaagc
246	Ath408_F_sp	cagggaacaagcagagcatgg
247	408_R_sp	gccagggaagaggcagtgcat
248	ppt156_F_csp	gtgaggcaatgcatgacaac
249	ppt156_R_csp	cagtgcatgaggacaacacc
250	ppt156_F_sp	ccagcgtgaggcaatgcatg
251	MIR156a_For	tgacagaagagagtgagcac
252	Ath_MIR156a _Rev	tctgacagaaagagcagtgagc
253	ppt_MIR156a _Rev	tgcgacaggaagagagtgagc
254	ath_MIR408 _For	cagggaacaagcagagcatgg
255	ppt_MIR408b_For	ccagcgtgaggcaatgcatg
256	MIR408b_Rev	agccagggaagaggcagtgca
257	miR8187_gen_3p_For	tgtacaactttaagatgacccatgata
258	miR8187_gen_3p_Rev	gtatattagaagccaaatgtgtcact
259	miR8178_gen_5p_For	ttttgaccgatgttttcgcatgatga
260	miR8178_gen_5p_Rev	tcgttgatgacttataggaccccaagat
261	408_gen_5p_For	ctaatacgactcactatagggcaagca
262	408_gen_5p_Rev	gttggaatatctcaaaggaaactggt
263	536_gen_5p_For	ctaatacgactcactatagggcaagca
264	536_gen_5p_Rev	gttggaatatctcaaaggaaactggt
265	536_gen_3p_For	ctaatacgactcactatagggcaagca
266	536_gen_3p_Rev	gttggaatatctcaaaggaaactggt
267	MIR8183.1_gen_5p_For	taaaaagtattcagtattcaagctcgaga
268	MIR8183.1_gen_5p_Rev	ggtaattgaagaggtaattggaaggttta
269	MIR8183.1_gen_3p_For	caaaggcctgttattatcgaagttctc
270	MIR8183.1_gen_3p_Rev	aggcaaattatattaaacacaggtcatca
271	MIR8186_gen_5p_For	ggatattttggttgacgtacaataatatga
272	MIR8186_gen_5p_Rev	aagtttttcaacagcggagaaggttat
273	MIR8186_gen_3p_For	gaaggatgcttgatatactcttgactgat
274	MIR8186_gen_3p_Rev	actcaagctatgcatccaacg
275	NS1	gtagtcatatgcttgtctc
276	VANS1	gtctagtataatcgttatacagg
277	NS2	ggctgctggcaccagacttgc
278	miR8187_CaGe_For	atccacttaccgttccttgg

279	miR8178_CaGe_Rev	aataggggtggaacaaattttctgg
280	miR408_CaGe_For	ggcccttctcttccgagtatc
281	408_CaGe_For	agggaacagacactgaattgga
282	408_CaGe_Rev	tggaatcaagatgctcaatatattact
283	UCP3_For	cgwattttvccdgagatatggc
284	UCP3_Rev	atgtatgckttttttagatcgt
285	UCP5_For	acttchggtgchaghgawataatt
286	UCP5_Rev	gaaachcgdatgggdtckgg
287	PSforward	gggattagataccccwgtagtcct
288	PSreverse	ccctaatctatggggwcatcagga
289	Uf	gagagtttgatcctggtcag
230	Ur	acggytaccttgttacgactt
231	PSkp1_For	gctatactacatcatccagtt
232	PSkpi1_Rev	aaacacaataactaccgcacg

Table S3 25 pri-miRNAs that were identified in the *P. endiviifolia* transcriptome. a – Precursors that were predicted for conservative miRNA in *P. endiviifolia*, b - Precursors that were predicted for novel miRNA in *P. endiviifolia*. cDNA IDs in bold represent the pre-miRNAs with secondary structures that are presented in Fig. S6. Other pre-miRNAs from the table that have the same pre-miRNA sequence and structure as their counterparts are marked in bold; however, they originate from different pri-miRNAs. In the case of two cDNA IDs for microRNA408b, two different pri-miRNAs having the same pre-miRNA sequence have been identified.

(a)

pre-miRNA	cDNA ID	pre-miRNA length (bp)	MFE	miR-5p			miR-3p		
				name	coords	mean counts (TPM)	name	coords	mean counts (TPM)
pen-miR156i	**pen_U1176**	104	-23.9	-	11..28	-	pen-miR156i	76..94	1.924
pen-miR156j				-	12..28	-	pen-miR156j	76..95	4.428
pen-miR390	**pen_U28358**	131	-30.8	pen-miR390	21..41	2199.484	-	93..110	-
pen-miR390a		131	-30.8	pen-miR390a	21..39	235.632	-	96..110	-
pen-miR408	**pen_C1398-2**	102	-57.1	pen-miR408	13..32	8285.828	pen-miR408	72..92	388.612
pen-miR408b		102	-57.1	pen-miR408b○	13..32	94669.436	pen-miR408b	73..93	42350.7
pen-miR408d		102	-57.1	pen-miR408d	13..31	472.366	pen-miR408d	73..92	512.646
pen-miR408	pen_C1398-4	102	-57.1	pen-miR408	13..32	8285.828	pen-miR408	72..92	388.612
pen-miR408b		102	-57.1	pen-miR408b○	13..32	94669.436	pen-miR408b	73..93	42350.7
pen-miR408d		102	-57.1	pen-miR408d	13..31	472.366	pen-miR408d	73..92	512.646
pen-miR408b	**pen_C1398-1 pen-MIR408b**	100	-49.9	pen-miR408b○	4..11	94669.436	pen-miR408b	72..92	697.546
pen-miR408b	pen_C1398-3 pen-MIR408b	100	-49.9	pen-miR408b○	4..11	94669.436	pen-miR408b	72..92	697.546
pen-miR536	**pen-MIR536**	157	-60.6	pen-miR536	4..25	7.742	phased pen-miR536	131..154	53.19
pen-miR7732	**pen_U39007**	55	-12.1	-	7..23	-	pen-miR7732	33..50	76.638

○ hybridized sRNA

(b)

pre-miRNA	cDNA ID	pre-miRNA length (bp)	MFE	miR-5p			miR-3p		
				name	coords	mean counts (TPM)	name	coords	mean counts (TPM)
pen-miR8159	**pen_U3835**	65	-9.4	pen-miR8159a○	8..28	298119.06	-	37..56	-
				pen-miR8159b○	8..27	116532.2	-	38..57	-
pen-miR8162	**pen-MIR8162**	89	-45.9	pen-miR8162-5p○	9..29	75985.524	pen-miR8162-3p○	64..84	65120.304
pen-miR8165	**pen-MIR8165**	129	-52.8	pen-miR8165○	10..30	59900.202	pen-miR8165	102..122	143.178
pen-miR8166	**pen_U10062**	211	-67.1	pen-miR8166	18..39	18.06	pen-miR8166○	176..197	245117.488
pen-miR8172	**pen_C578-1**	834	-365.0	pen-miR8172.1○	9..29	57485.066	pen-miR8172.1	808..828	1291.39
				pen-miR8172.2	25..45	3.53	pen-miR8172.2○	792..812	14799.006
	**pen_C578-2**	834	-365.0	pen-miR8172.1○	9..29	57485.066	pen-miR8172.1	808..828	1291.39
				pen-miR8172.2	25..45	3.53	pen-miR8172.2○	792..812	14799.006
pen-miR8178	**pen-MIR8178**	86	-39.1	pen-miR8178○	15..35	1409.482	pen-miR8178	54..74	26231.568
pen-miR8182	**pen_C2867-1**	116	-41.1	pen-miR8182	13..33	1724.464	pen-miR8182○	85..105	89872.874
	pen_C2867-2	116	-41.1	pen-miR8182	13..33	1724.464	pen-miR8182○	85..105	89872.874
	pen_C2867-3	116	-41.1	pen-miR8182	13..33	1724.464	pen-miR8182○	85..105	89872.874
	pen_C2867-4	116	-41.1	pen-miR8182	13..33	1724.464	pen-miR8182○	85..105	89872.874
pen-miR8183	**pen-MIR8183**	94	-52.8	pen-miR8183.2○	15..35	111970.026	pen-miR8183.2	62..82	1173.286
	pen_C2351-2	94	-52.8	pen-miR8183.2○	15..35	111970.026	pen-miR8183.2	62..82	1173.286
pen-miR8185	**pen-MIR8185**	88	-26.1	pen-miR8185	4..24	582.302	pen-miR8185○	67..87	21593.696
pen-miR8186	**pen-MIR8186**	94	-45.4	pen-miR8186○	15..35	29270.628	pen-miR8186	61..81	271.562
pen-miR8187	**pen-MIR8187**	96	-47.6	pen-miR8187	18..43	41.704	pen-miR8187○	59..82	7878.44
pen-miR8190	**pen-MIR8190**	80	-40.4	pen-miR8190○	8..29	9799.494	pen-miR8190	54..75	143.24

○ hybridized sRNA

Table S4 High expression of pen-miR8162-5p:pen-miR8162-3p in all of the sRNA NGS datasets. The read counts in each sample were normalized to the total identified miRNAs counts in the sample and are presented in TP1M (tag per 1 million) form.

miRNA name	sequence	length	Female from *in vitro*	Male from the environment	Male from *in vitro*	Female from the environment	Female from *in vitro*	mean counts
pen-miR8162-5p	tttgtaagaattggaaccgga	21	76876.23	51560.86	144441.15	61826.68	45222.7	75985.524
pen-miR8162-3p	tggatccatttcttacagacg	21	11226.93	59086.13	135350.99	65668.31	54269.16	65120.304

Table S5

Target mRNAs of the known miRNAs that were identified in *P. endiviifolia* and confirmed by the degradome data. The gray color indicates conserved targets across land plants. Below the table, T-plots for identified targets are presented.

miRNA family	miRNA name	Target	PFAM domain / family	Molecular function	Cleavage site	Cleavage region
miR156	pen-miR156a	pen_U37761	Protein of unknown function (DUF1218)	-	913	CDS
	pen-miR156ad	pen_C10956-2	Membrane transport protein	-	287	5'UTR
		pen_C10956-3		-	287	CDS
miR159	pen-miR159b	pen_U38944	-	-	803	CDS
miR160	pen-miR160a,b,c,d,e,f,g	pen_U38297	B3 DNA binding domain<br>Auxin response factor	DNA binding	2129	CDS
	pen-miR160c	pen_C7409-1	Dirigent-like protein	-	200	CDS
miR165/166	pen-miR165a<br>pen-miR166a,b,c,d,e,g,i,j,m	pen_U34898	Homeobox domain<br>START domain<br>MEKHLA domain	sequence-specific DNA binding transcription factor activity<br>lipid binding	706	CDS
miR169	pen-miR169t	pen_U8300	Redoxin	oxidoreductase activity	760	CDS
miR319	pen-miR319,c	pen_C9156-1	RWP-RK domain	-	2138	3'UTR
	pen-miR319d.1	pen_U37540	Zinc-finger (CX5CX6HX5H) motif	-	1482	3'UTR
miR390	pen-miR390a	pen_U32458	-	-	118	CDS
miR391	pen-miR391	pen_U34504	S4 domain	RNA binding	864	CDS
miR396	pen-miR396b	pen_C3067-1	-	-	939	CDS
	pen-miR396d	pen_U7769	Leucine rich repeat N-terminal domain	protein binding	3345	CDS
miR397	pen-miR397b	pen_U7431	Domain of unknown function (DUF4094)	Galactosyltransferase	3761	3'UTR
miR399	pen-miR399b	pen_U16456	Pectinesterase	pectinesterase activity	2268	3'UTR
miR408	pen-miR408	pen_U31498	Plastocyanin-like domain	copper ion binding<br>electron carrier activity	430	5'UTR
	pen-miR408a,b,d	pen_C6396-1	Plastocyanin-like domain	copper ion binding<br>electron carrier activity	203	CDS
		pen_C6396-2			203	CDS
	pen-miR408,408a,b,d,e	pen_C7900-2	56kDa selenium binding protein (SBP56)	selenium binding	252	5'UTR
	pen-miR408,408a,d,e	pen_C7408-1	Heavy-metal-associated domain<br>P-type ATPase ATP binding domain<br>haloacid dehalogenase-like hydrolase	nucleotide binding<br>metal ion binding	2814	CDS
		pen_C7408-2			2786	CDS
	pen-miR408b,d	pen_U15272	Common central domain of tyrosinase<br>Polyphenol oxidase middle domain<br>Protein of unknown function (DUF B2219)	oxidoreductase activity<br>catechol oxidase activity	1375	3'UTR
		pen_U21649	-	-	593	CDS

		pen_U31467	-	-	1341	CDS
		pen_U35485	Protein of unknown function (DUF B2219)	catechol oxidase activity	367	3'UTR
miR414	pen-miR414	pen_C1936-3	-	-	788	CDS
		pen_C1936-4	-	-	628	CDS
		pen_U7297	Protein of unknown function (DUF3464)	-	638	CDS
		pen_U7909	-	-	955	CDS
		pen_U19831	DEK C terminal domain		2988	CDS
		pen_U34431	Leucine rich repeat N-terminal domain<br>Protein tyrosine kinase	protein kinase activity<br>protein binding	3451	CDS
miR472	pen-miR472	pen_C190-3	NB-ARC domain	ADP binding	1336	CDS
miR482	pen-miR482a	pen_C2791-1	Aminomethyltransferase folate-binding domain<br>Glycine cleavage T-protein C-terminal barrel domain	aminomethyltransferase activity	1001	CDS
		pen_C2791-2	Glycine cleavage T-protein C-terminal barrel domain	-	232	CDS
		pen_C2791-3	-	-	232	CDS
	pen-miR482a.1,c	pen_C190-3	NB-ARC domain	ADP binding	1336	CDS
miR529	pen-miR529e	pen_C2490-1	F-box domain	protein binding	1272	5'UTR
		pen_C2490-2			1017	5'UTR
miR536	pen-miR536,536c	pen_C8300-2	-	-	902	3'UTR
	pen-miR536c	pen_U11189	-	-	1707	CDS
miR902	pen-miR902a	pen_U8046	-	-	425	CDS
miR904	pen-miR904a	pen_C7183-1	Ribosomal protein S27	structural constituent of ribosome	230	CDS
miR1063	pen-miR1063a	pen_C2103-1	60S ribosome biogenesis protein Rrp14<br>Surfeit locus protein 6	-	896	CDS
		pen_C2103-2			896	CDS
miR1216	pen-miR1216	pen_U15556	Methyltransferase FkbM domain	-	1790	CDS
miR1222	pen-miR1222a	pen_C3471-1	Dymeclin	-	926	CDS
miR1863	pen-miR1863b.2	pen_U34447	Golgi complex component 7 (COG7)	-	3477	3'UTR
miR1874	pen-miR1874	pen_C9443-1	PPR repeat	-	584	CDS
		pen_U15736	PPR repeat	-	461	CDS
miR2108	pen-miR2108a	pen_U37608	-		846	3'UTR
miR2111	pen-miR2111	pen_C3870-2	-	-	113	CDS
		pen_U30499	Sulfite exporter TauE/SafE	-	2115	3'UTR
miR2118	pen-miR2118	pen_C190-3	NB-ARC domain	ADP binding	1336	CDS
	pen-miR2118a,e,p	pen_C168-1	Protein kinase domain	protein kinase activity<br>ATP binding	2511	CDS
		pen_C168-2			1241	CDS

		pen_C168-3			1240	CDS
	pen-miR2118e,p	pen_C2791-1	Aminomethyltransferase folate-binding domain<br>Glycine cleavage T-protein C-terminal barrel domain	aminomethyltransferase activity	1001	CDS
		pen_C2791-2	Glycine cleavage T-protein C-terminal barrel domain	-	232	CDS
		pen_C2791-3	-	-	232	CDS
miR2673	pen-miR2673a	pen_C1181-1	Protein kinase domain	protein kinase activity<br>ATP binding	1793	CDS
		pen_C1181-2			1827	CDS
miR3509	pen-miR3509	pen_C3384-2	SNARE associated Golgi protein	-	504	5'UTR
miR3948	pen-miR3948	pen_C6471-1	-	-	2009	CDS
miR4414	pen-miR4414	pen_U23973	Domain of unknown function (DUF3508)	-	188	CDS
miR5021	pen-miR5021	pen_U35171	-	-	784	3'UTR
miR5269	pen-miR5269a	pen_U16701	C2 domain	protein binding	2346	3'UTR
miR5298	pen-miR5298b	pen_U37595	Cupin	nutrient reservoir activity	1066	3'UTR
miR5562	pen-miR5562	pen_U16235	TOM7 family	-	336	CDS
miR5673	pen-miR5673	pen_U35027	Uncharacterized ACR, COG1678	-	53	5'UTR
miR5754	pen-miR5754	pen_U37701	APO RNA-binding	RNA binding	1063	CDS
miR5815	pen-miR5815	pen_U38123	bHLH-MYC and R2R3-MYB transcription factors N-terminal	-	2398	CDS
miR6116	pen-miR6116	pen_C3133-3	-	-	1077	CDS
		pen_U15710	Fructose-bisphosphate aldolase class-I	fructose-bisphosphate aldolase activity	612	CDS
miR6463	pen-miR6463	pen_U35118	Phospholipase D-nuclease N-terminal	-	1488	CDS
miR6486	pen-miR6468	pen_C3112-1	-	-	819	3'UTR
miR7755	pen-miR7755	pen_U2937	Sir2 family	NAD+ binding	1264	3'UTR

miR156

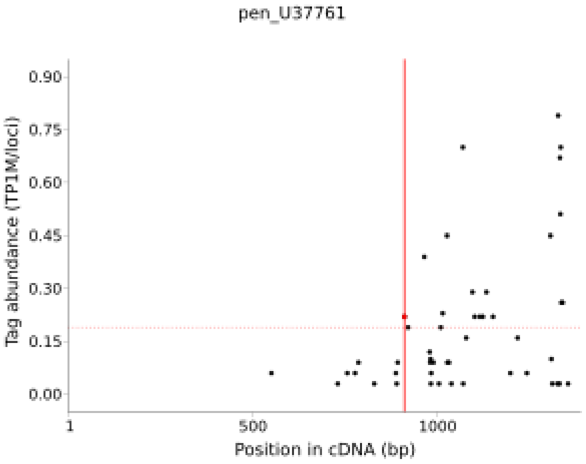


```
                 pen-miR156a
3'            CACGAGUGAGAGAAGACAGU              5'
              -||||-||||||||||-||-
5' UGGGUUUUUAGCCUUGGAUGCU-ACUCUCUUCUUUCUGAUUUCCCUCAUG 3'
                 pen_U37761
```

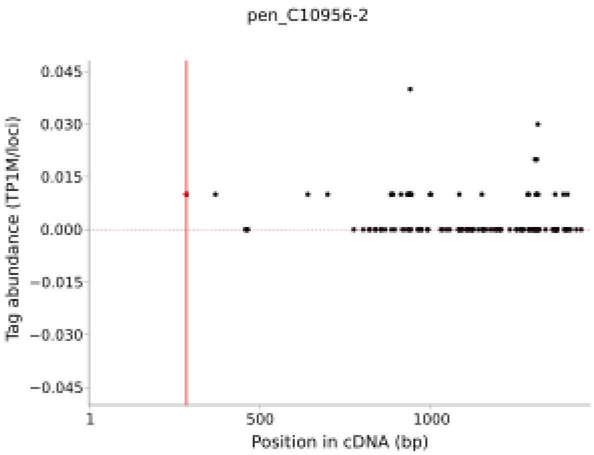


```
                 pen-miR156ad
3'             CACGAGUGACAGAAGACAGU            5'
               -||||-:||||-||||||||
5' GUCACCUCCUGGUGGUUCUGCU-GCUGU-UUCUGUCAAUGCUACUGCACC 3'
                 pen_C10956-2
```

miR159

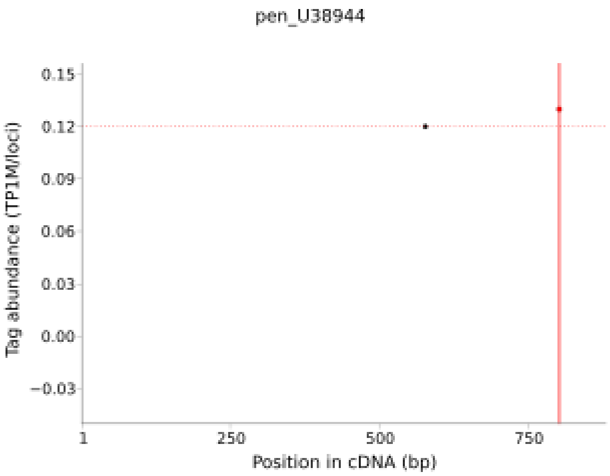


```
                  pen-miR159b
3'              CCCUCGAGAGAAGUAAGGUUC              5'
                -||||||||||||-||||||-
5' CAGAUCGAACUCAACAAAGGAGCUCUCUUCUUUCCAAAGCUACCCAAGCCA 3'
                  pen_U38944
```

miR160

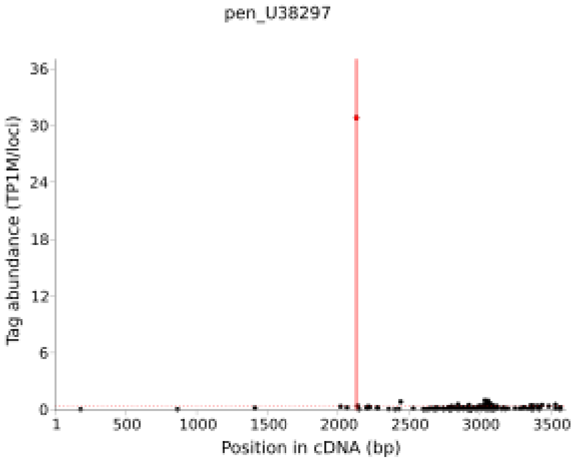


```
                  pen-miR160a
3'             ACCGUAUGUCCCUCGGUCCGU            5'
               -||||||||||||||||||||
5' AUAGAGGAUAUUCCUUCGGGCAUACAGGGAGCCAGGCAUGAUCGAGUUGUU 3'
                  pen-miR160b
3'             ACCGUAUGUCCCUCGGUCCGC            5'
               -|||||||||||||||||||-
5' AUAGAGGAUAUUCCUUCGGGCAUACAGGGAGCCAGGCAUGAUCGAGUUGUU 3'
                  pen-miR160c
3'             ACCGUAGGUCCCUCGGUCCGC            5'
               -|||||-|||||||||||||-
5' AUAGAGGAUAUUCCUUCGGGCAUACAGGGAGCCAGGCAUGAUCGAGUUGUU 3'
                  pen-miR160d
3'             ACCGUACGUCCCUCGGUCCGU            5'
               -|||||-||||||||||||||
5' AUAGAGGAUAUUCCUUCGGGCAUACAGGGAGCCAGGCAUGAUCGAGUUGUU 3'
                  pen-miR160e
3'             GCCGUAUGUCCCUCGGUCCGU            5'
               -||||||||||||||||||||
5' AUAGAGGAUAUUCCUUCGGGCAUACAGGGAGCCAGGCAUGAUCGAGUUGUU 3'
                  pen-miR160f
3'             ACCGUAAGUCCCUCGGUCCGU            5'
               -|||||-||||||||||||||
5' AUAGAGGAUAUUCCUUCGGGCAUACAGGGAGCCAGGCAUGAUCGAGUUGUU 3'
                  pen-miR160g
3'             ACCGUAUGUUCCUCGGUCCGU            5'
               -||||||||:|||||||||||
5' AUAGAGGAUAUUCCUUCGGGCAUACAGGGAGCCAGGCAUGAUCGAGUUGUU 3'
                  pen_U38297
```

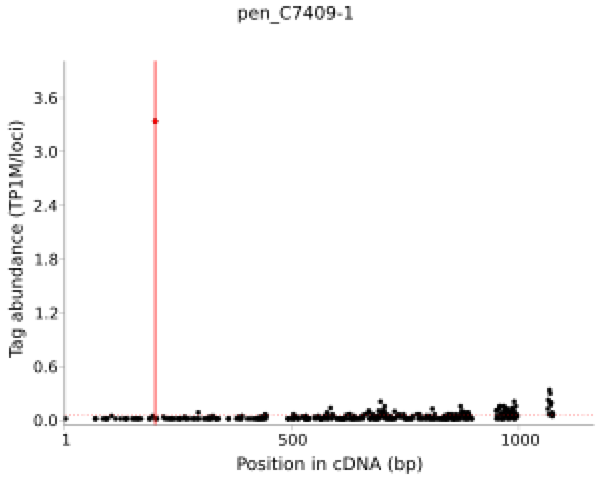


```
                pen-miR160c
3'          ACCGUAGGUCCCUCGGUCCGC          5'
            -|||:-||||||||||||||-
5' GGAGCCAUGAGCCUCCGAGGCGCCCAGGGAGCCAGGCACCAUCGCGCGAAG 3'
                pen_C7409-1
```

miR165 / 166

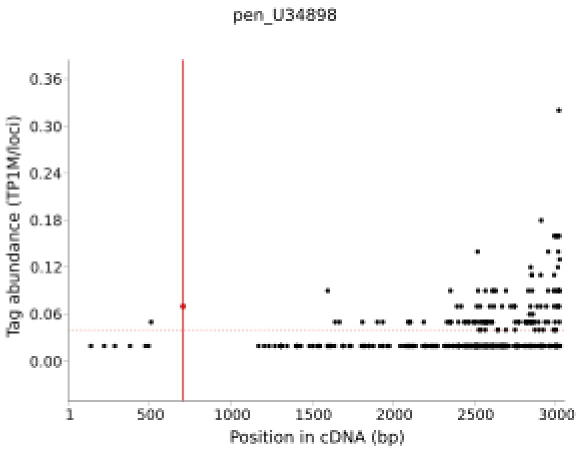


```
                pen-miR165a
3'          CCCCUACUUCGGACCAGGCUU          5'
            -||||||||||||||||||:|
5' GACUGGAUCCAAAUGCCCGGGAUGAAGCCUGGUCCGGACUCCAUAGGCAUU 3'

                pen-miR166a
3'          CCCCUUACUUCGGACCAGGCU          5'
            --||:|||||||||||||||:
5' UGACUGGAUCCAAAUGCCCGGGAUGAAGCCUGGUCCGGACUCCAUAGGCAU 3'
                pen-miR166b
3'          UUCCUUACUUCGGACCAGGCU          5'
            --||:|||||||||||||||:
5' UGACUGGAUCCAAAUGCCCGGGAUGAAGCCUGGUCCGGACUCCAUAGGCAU 3'
                pen-miR166c
3'          UCCUUACUUCGGACCAGGCU          5'
            -||:|||||||||||||||:
5' GACUGGAUCCAAAUGCCCGGGAUGAAGCCUGGUCCGGACUCCAUAGGCAU 3'
                pen-miR166d
3'          GCCCUUACUUCGGACCAGGCU          5'
            |-||:|||||||||||||||:
5' UGACUGGAUCCAAAUGCCCGGGAUGAAGCCUGGUCCGGACUCCAUAGGCAU 3'
                pen-miR166e
3'          CCCCUUACUUCGGACCAAGCU          5'
            --||:||||||||||||-||:
5' UGACUGGAUCCAAAUGCCCGGGAUGAAGCCUGGUCCGGACUCCAUAGGCAU 3'
                pen-miR166g
3'          CUCCUUACUUCGGACCAGGCU          5'
            --||:|||||||||||||||:
5' UGACUGGAUCCAAAUGCCCGGGAUGAAGCCUGGUCCGGACUCCAUAGGCAU 3'
                pen-miR166i
3'          CUCCUUACUUCGGACUAGGCU          5'
            --||:||||||||||:||||:
5' UGACUGGAUCCAAAUGCCCGGGAUGAAGCCUGGUCCGGACUCCAUAGGCAU 3'
                pen-miR166j
3'          CCCUUACUUCGGACCAGGCCU          5'
            -||:|||||||||||||||||
5' GACUGGAUCCAAAUGCCCGGGAUGAAGCCUGGUCCGGACUCCAUAGGCAUU 3'
                pen-miR166m
3'          UCCCUUACUUCGGACCAGGCU          5'
            --||:|||||||||||||||:
5' UGACUGGAUCCAAAUGCCCGGGAUGAAGCCUGGUCCGGACUCCAUAGGCAU 3'
                pen_U34898
```

miR169

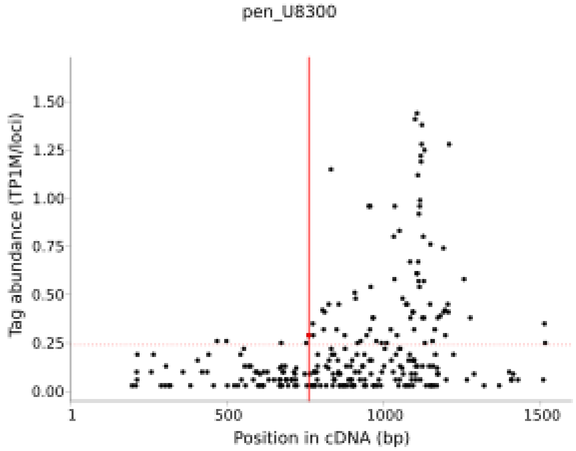


```
                pen-miR169t
3'          UGUUUAGGUAGGAACCGAU          5'
            |||||--|||-|||||||-
5' CUUUGAUGGAAAUUUCCACAAA--CAU-CUUGGCUUAGAAUUUGACUUG 3'
                pen_U8300
```

miR319

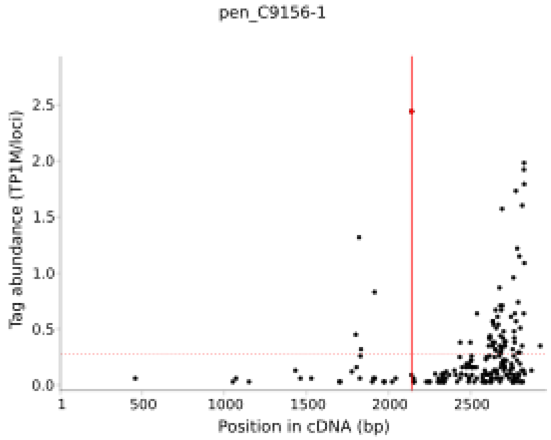


```
                  pen-miR319
3'                    CCCUCGAGGGAAGUCAGGUU                5'
                      -|||-||||||||-||||||
5' CCCUAGUUUCAUGACAAUGGAACUCCCUUCCGUCCAAAAUUUCCUCAGAA 3'
                  pen-miR319c
3'                    CCCUCGAGGGAAGUCAGGUUC                5'
                      -|||-||||||||-||||||-
5' CCCUAGUUUCAUGACAAUGGAACUCCCUUCCGUCCAAAAUUUCCUCAGAAG 3'
                  pen_C9156-1
```

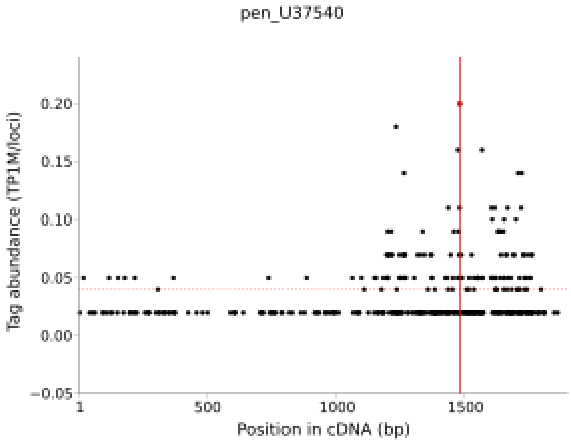


```
                       pen-miR319d.1
3'                    AUAACUUGGCUUCCUUCGA                5'
                      |:|-|||-||-||||||||
5' GCCAGUGGGCCCCCUUUUGUAGAA-CGCAGGAAGCUGCUAUGUUAUGAA 3'
                          pen_U37540
```

miR390

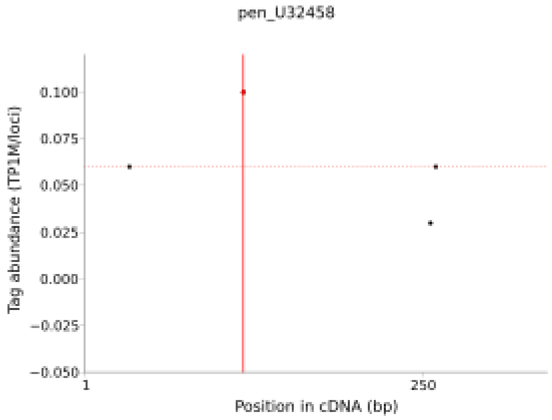


```
                       pen-miR390a
3'                    GCGAUAGGGAGGACUCGAA                5'
                      :|||:-|||||||||-||-
5' ACCAAUCGCUUUGUGGCUGCUGACCCUCCUGACCUGCUCAACUUCGAGG 3'
                         pen_U32458
```

miR391

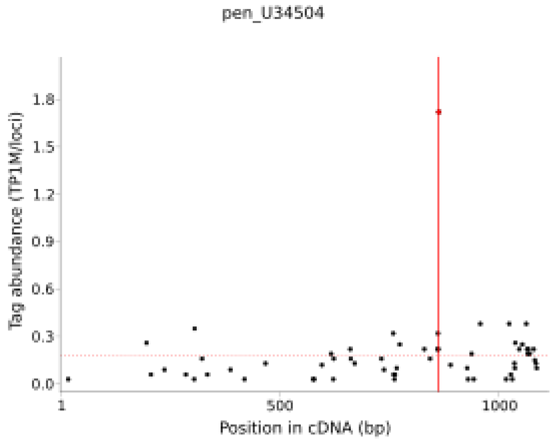


```
                        pen-miR391
3'                    ACCGCGAUAGAGAGGACGCUU                5'
                      ||-:|:||||||-|||||||:
5' GCAGCCCUGAAGUCGGGUGAUGUUAUCUC-CCUGCGAGGGAAAGGAAGAAU 3'
                         pen_U34504
```

miR396

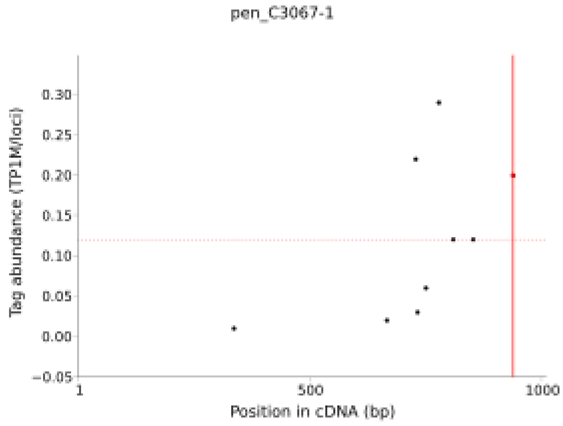

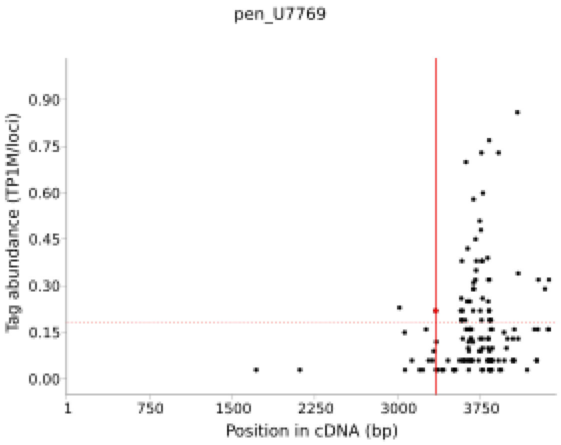


```
                  pen-miR396b
3'           UUCAAGUUCUUUCGACACCUU           5'
             |:|-|-|||||||||-||||||
5' UACAUGGAGAAUUUACCAGGAUGAAGAAAGCAGUGGAAGCUGCAGAAAAGA 3'
                  pen_C3067-1
```

```
                  pen-miR396d
3'             UAAGAAGGUGUAGAAAGA            5'
               |-||||:||:||||||||
5' GUGGACCUAUCCCCCGAAGUCUUUCAUAUCUUUCUCAGCUUGAGUCAC 3'
                   pen_U7769
```

miR397

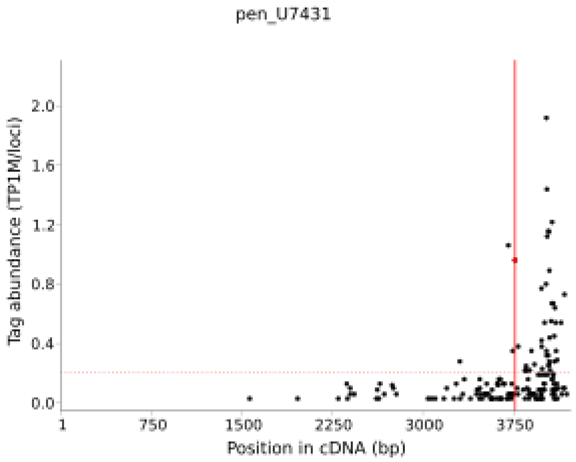


```
                  pen-miR397b
3'             GUUGUUGCUACGUAAGUUA             5'
               -|:|||-|||||||-||||
5' AUGUAUUCUCAUAUUGGAAGCAAAGAUGCAUCCAAUUUUCCACAUUGGA 3'
                   pen_U7431
```

miR399

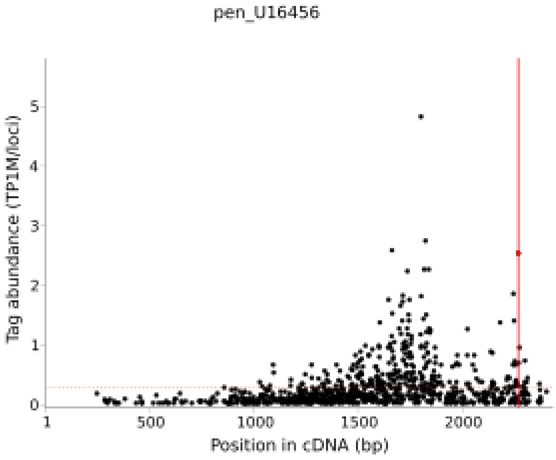


```
                  pen-miR399b
3'              ACGGGCUCCUCUCCACGU              5'
                |-||||-|||||||||-|
5' AGCAUUUUACACGAAGCUUCCCGCGGAGAGGUGGAAGUUCGAUCUCCA 3'
                   pen_U16456
```

miR408

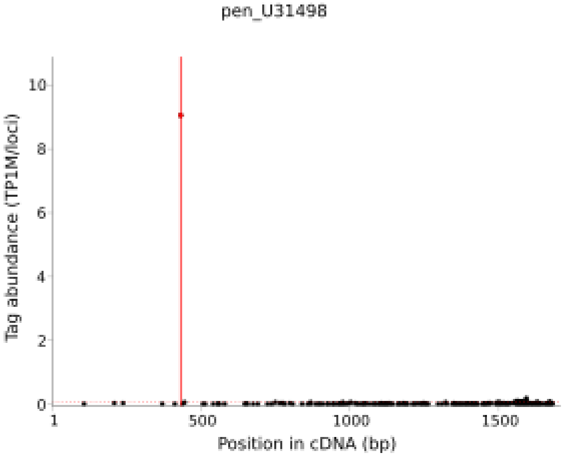

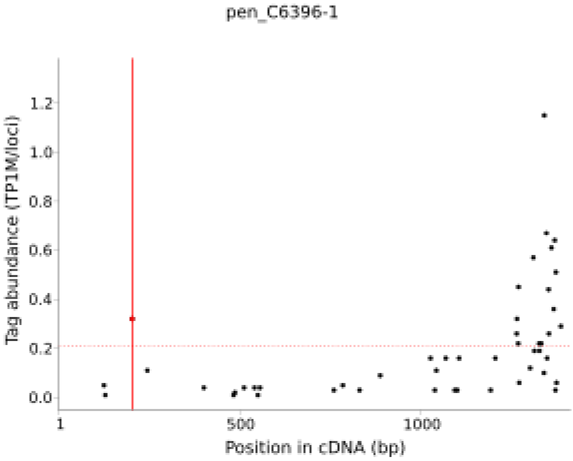


```
                    pen-miR408
3'                 CGGUCCCUUCUCCGUCACGUA               5'
                   |||:||-||-|||||||||||
5' CGGGAACUGAAUCUUUAGCCGGGAAAAAGGCAGUGCAUCGAACGAGAAUGG 3'
                    pen_U31498
```

```
                    pen-miR408a
3'                 UUGGUCCCUUCUCCGUCACGU               5'
                   |:|-|||||-||-||||||||
5' UGGCGAGUCAGAUGGCGAGCGAGGGACGACGCAGUGCAUUGAGAUCGCACU 3'
                    pen-miR408b
3'                 UCGGUCCCUUCUCCGUCACGU               5'
                   |||-|||||-||-||||||||
5' UGGCGAGUCAGAUGGCGAGCGAGGGACGACGCAGUGCAUUGAGAUCGCACU 3'
                    pen-miR408d
3'                 CGGUCCCUUCUCCGUCACGU               5'
                   ||-|||||-||-||||||||
5' GGCGAGUCAGAUGGCGAGCGAGGGACGACGCAGUGCAUUGAGAUCGCACU 3'
                    pen_C6396-1
```

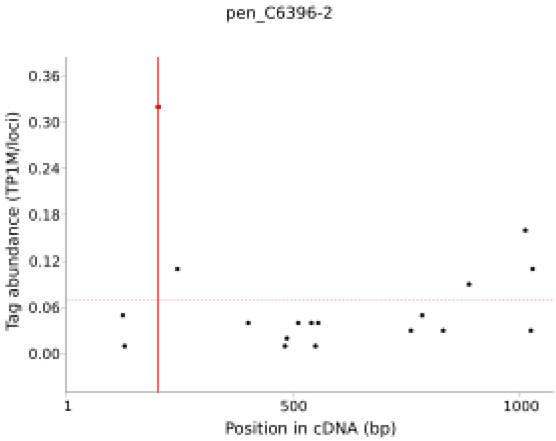

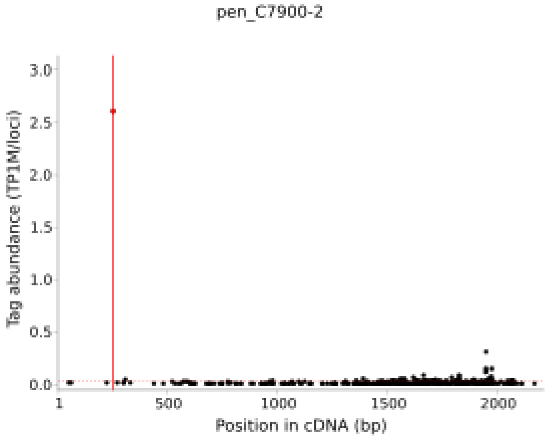


```
                    pen-miR408a
3'                 UUGGUCCCUUCUCCGUCACGU               5'
                   |:|-|||||-||-||||||||
5' UGGCGAGUCAGAUGGCGAGCGAGGGACGACGCAGUGCAUUGAGAUCGCACU 3'
                    pen-miR408b
3'                 UCGGUCCCUUCUCCGUCACGU               5'
                   |||-|||||-||-||||||||
5' UGGCGAGUCAGAUGGCGAGCGAGGGACGACGCAGUGCAUUGAGAUCGCACU 3'
                    pen-miR408d
3'                 CGGUCCCUUCUCCGUCACGU               5'
                   ||-|||||-||-||||||||
5' GGCGAGUCAGAUGGCGAGCGAGGGACGACGCAGUGCAUUGAGAUCGCACU 3'
                    pen_C6396-2
```

```
                    pen-miR408
3'                 CGGUCCCUUCUCCGUCACGUA               5'
                   --||||||||||||:|||||-
5' UGCGCGUGUGUCAGACAAACAGGGAAGAGGCGGUGCAGUGGCGUCACCGUA 3'
                    pen-miR408a
3'                 UUGGUCCCUUCUCCGUCACGU               5'
                   ||-||||||||||||:|||||
5' GUGCGCGUGUGUCAGACAAACAGGGAAGAGGCGGUGCAGUGGCGUCACCGU 3'
                    pen-miR408b
3'                 UCGGUCCCUUCUCCGUCACGU               5'
                   |--||||||||||||:|||||
5' GUGCGCGUGUGUCAGACAAACAGGGAAGAGGCGGUGCAGUGGCGUCACCGU 3'
                    pen-miR408d
3'                 CGGUCCCUUCUCCGUCACGU               5'
                   --||||||||||||:|||||
5' UGCGCGUGUGUCAGACAAACAGGGAAGAGGCGGUGCAGUGGCGUCACCGU 3'
                    pen-miR408e
3'                 CGGUCCCUUCUCAGUCACGU               5'
                   --||||||||||-|:|||||
5' UGCGCGUGUGUCAGACAAACAGGGAAGAGGCGGUGCAGUGGCGUCACCGU 3'
                    pen_C7900-2
```

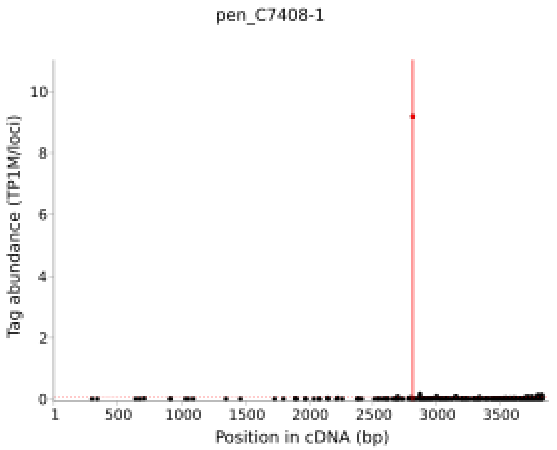


```
                       pen-miR408
3'                  CGGUCCCUUCUCCGUCACGUA              5'
                    -:||||||:||||||||||||
5' UGGCAGAUGAAGUUGGCAUCAGGGAGGAGGCAGUGCAUGCUGAGUUGCGUC 3'
                       pen-miR408a
3'                  UUGGUCCCUUCUCCGUCACGU              5'
                    -|:||||||:|||||||||||
5' GUGGCAGAUGAAGUUGGCAUCAGGGAGGAGGCAGUGCAUGCUGAGUUGCGU 3'
                       pen-miR408d
3'                  CGGUCCCUUCUCCGUCACGU              5'
                    -:||||||:|||||||||||
5' UGGCAGAUGAAGUUGGCAUCAGGGAGGAGGCAGUGCAUGCUGAGUUGCGU 3'
                       pen-miR408e
3'                  CGGUCCCUUCUCAGUCACGU              5'
                    -:||||||:|||-|||||||
5' UGGCAGAUGAAGUUGGCAUCAGGGAGGAGGCAGUGCAUGCUGAGUUGCGU 3'
                        pen_C7408-1
```

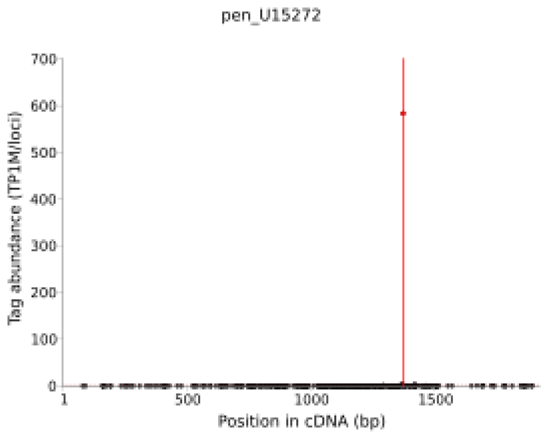

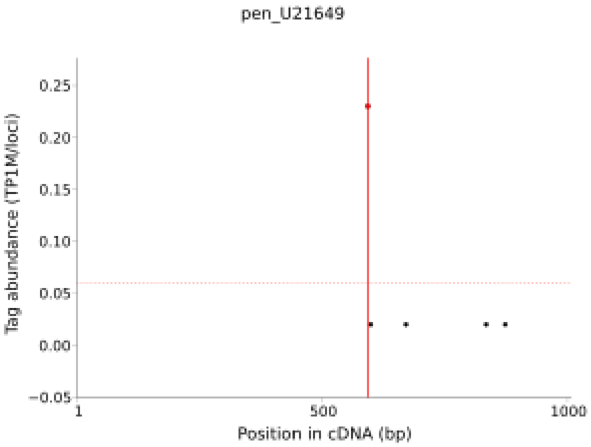


```
                       pen-miR408b
3'                  UCGGUCCCUUCUCCGUCACGU              5'
                    -||:-|||||||-||||||||
5' UCCGCGUGAGAAGCUCGCGCUCGGGAAGACGCAGUGCAGUCGCGGGAUGUA 3'
                       pen-miR408d
3'                  CGGUCCCUUCUCCGUCACGU              5'
                    ||:-|||||||-||||||||
5' CCGCGUGAGAAGCUCGCGCUCGGGAAGACGCAGUGCAGUCGCGGGAUGUA 3'
                       pen_U15272
```

```
                       pen-miR408b
3'                  UCGGUCCCUUCUCCGUCACGU              5'
                    |||-||||||||||||-||-|
5' ACGGAGCGAGAGUUGAUAGCAAGGGAAGAGGCAAUGGAGAGGUUGACUCGC 3'
                       pen-miR408d
3'                  CGGUCCCUUCUCCGUCACGU              5'
                    ||-||||||||||||-||-|
5' CGGAGCGAGAGUUGAUAGCAAGGGAAGAGGCAAUGGAGAGGUUGACUCGC 3'
```

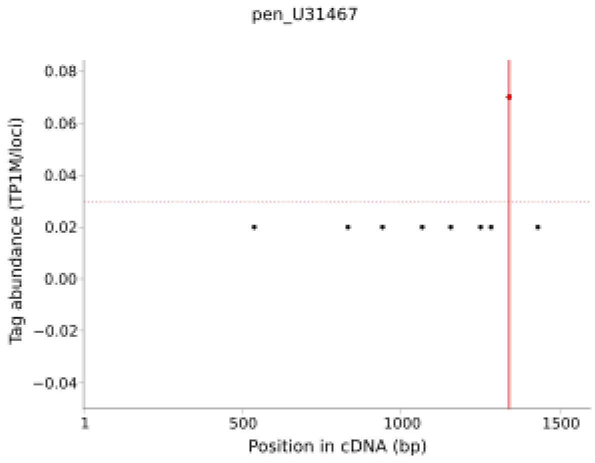

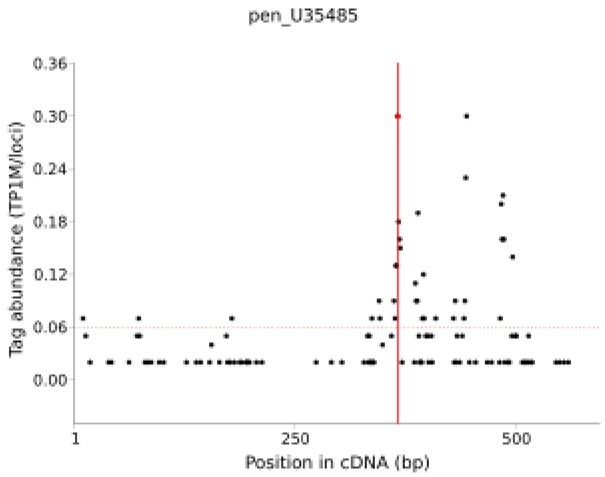


```
                       pen-miR408b
3'                  UCGGUCCCUUCUCCGUCACGU              5'
                    ||:-||||||||-|||-||||
5' CAAUCUGGAGCACUACCAGUGAGGGAAGAAGCACUGCACCAAGGGAAAGGA 3'
                       pen-miR408d
3'                  CGGUCCCUUCUCCGUCACGU              5'
                    |:-||||||||-|||-||||
5' AAUCUGGAGCACUACCAGUGAGGGAAGAAGCACUGCACCAAGGGAAAGGA 3'
                        pen_U31467
```

```
                       pen-miR408b
3'                  UCGGUCCCUUCUCCGUCACGU              5'
                    -||:-|||||||-|||||||:
5' UUAGUGAUCGAGCACUCCGCUUGGGAAGAUGCAGUGCGAUGUGCUUUAAAA 3'
                       pen-miR408d
3'                  CGGUCCCUUCUCCGUCACGU              5'
                    ||:-|||||||-|||||||:
5' UAGUGAUCGAGCACUCCGCUUGGGAAGAUGCAGUGCGAUGUGCUUUAAAA 3'
                        pen_U35485
```

miR414

pen_C1936-3

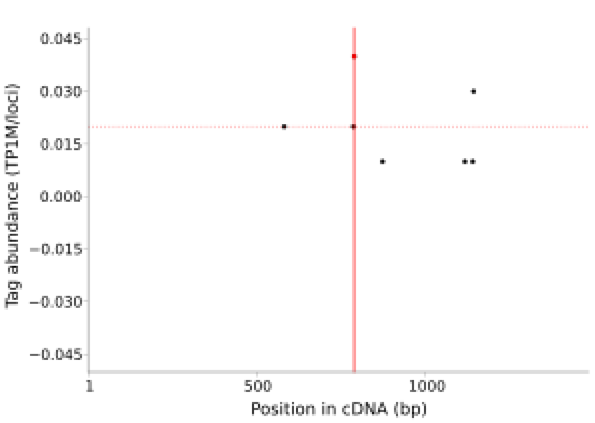

pen_U7297

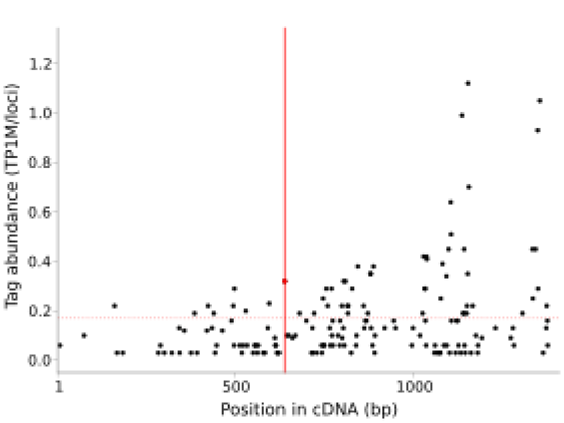


```
                 pen-miR414
3'           UGCUGCUACUACUACUACU            5'
             ::||:||||||||||||||
5' CAUGGAAAGUCUCGACAGUGAUGAUGAUGAUGAUGAUAUACAGGAAAGG 3'
                 pen_C1936-3
```

```
                 pen-miR414
3'           UGCUGCUACUACUACUACU            5'
             |:|:|||-||-|||||||-
5' AGGAGUGAAAUCCCAGGAUGGCGAAGACGAUGAUGUGGUUCCAGAGGUU 3'
                 pen_U7297
```

pen_U7909

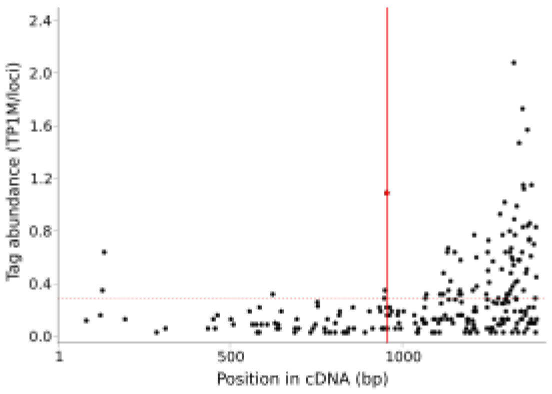

pen_U19831

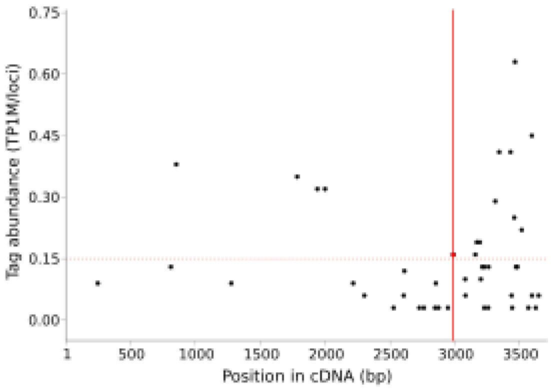


```
                 pen-miR414
3'           UGCUGCUACUACUACUACU            5'
             :-||:||-|||||-|||||
5' UGGUGGACCGGGUUUCGGAGAUGAGGAUGAAGAUGAAGAUGAGGAUGAA 3'
                 pen_U7909
```

```
                 pen-miR414
3'           UGCUGCUACUACUACUACU            5'
             --||:|||||-||||||||
5' UAAGCUCGCUGAAGAUGUGGAUGAUGAGGAUGAUGACCAAGAAGGUACA 3'
                 pen_U19831
```

pen_U34431

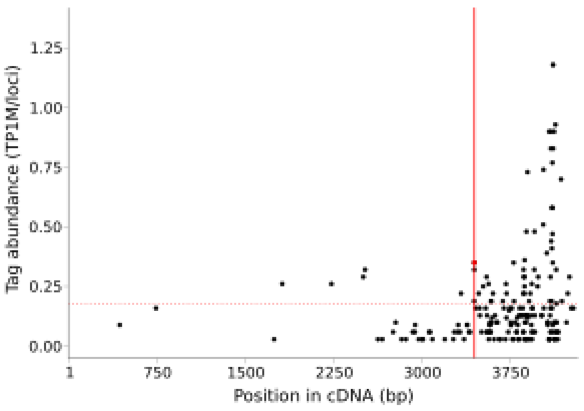


```
                 pen-miR414
3'           UGCUGCUACUACUACUACU            5'
             |-||-||-||-||||||||
5' GGGGGCUGCACAUCAUCAAGAGGAAGA-GAUGAUGAACACCUUGCAGCU 3'
                 pen_U34431
```

miR472 / 482 / 2118

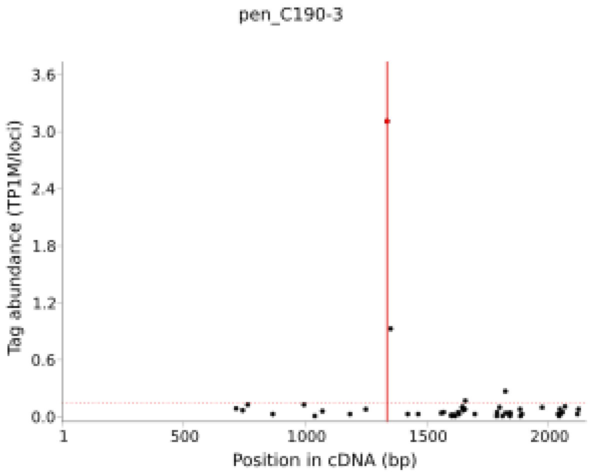


```
                    pen-miR472
3'                  AUACCCGCCUUAUCCUUU              5'
                    -|||||-||||||||:||
5' GCAGUCCUUCUACAUGGGAUGGGAGGAAUAGGGAAGACCACCCUGGCC 3'
                    pen_C190-3

                    pen-miR482a.1
3'                  GUACCAUACCCUCCUUAUCCCUU              5'
                    |||||-||||||||||||||||
5' UGGAAGCAGUCCUUCUACAUGGGAUGGGAGGAAUAGGGAAGACCACCCUGGCC 3'
                    pen-miR482c
3'                  CCUUACCCGCCUUAUCCCUU              5'
                    ||:|||||-|||||||||||
5' AAGCAGUCCUUCUACAUGGGAUGGGAGGAAUAGGGAAGACCACCCUGGCC 3'
                    pen_C190-3

                    pen-miR2118
3'                  GUACCAUACCCACCUUAUCCCUU              5'
                    |||||-|||||-|||||||||||
5' UGGAAGCAGUCCUUCUACAUGGGAUGGGAGGAAUAGGGAAGACCACCCUGGCC 3'
                    pen_C190-3
```

miR482 / 2118

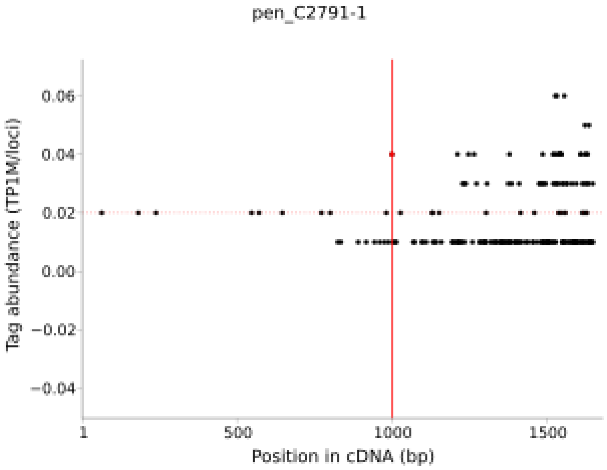


```
                    pen-miR482a
3'                  CCUUACCCACCGUAACCUUU              5'
                    ||-||||-|||||||||-|-
5' UGCUCCUGGAAACAAUAGGCAUGGCUGGCAUUGGUACAAGGAUCCACGGU 3'
                    pen_C2791-1

                    pen-miR2118e
3'                  AUCCGUACCCACCGUAACCCUU              5'
                    |||||||||-|||||||||-|-
5' AGUGCUCCUGGAAACAAUAGGCAUGGCUGGCAUUGGUACAAGGAUCCACGGU 3'
                    pen_C2791-1

                    pen-miR2118p
3'                  AUCCGUACCCACCGUAGCCCUU              5'
                    |||||||||-||||||:||-|-
5' AGUGCUCCUGGAAACAAUAGGCAUGGCUGGCAUUGGUACAAGGAUCCACGGU 3'
                    pen_C7291-1
```

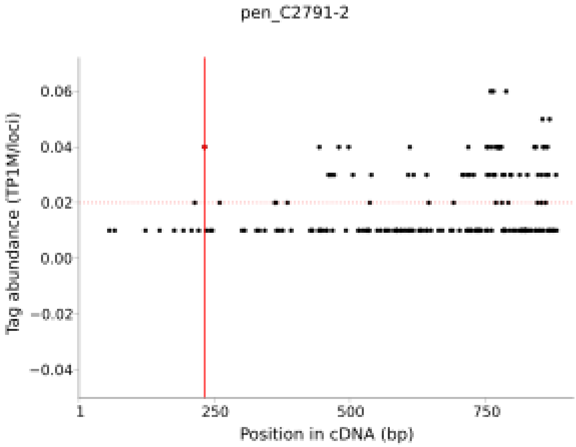


```
                    pen-miR482a
3'                  CCUUACCCACCGUAACCUUU              5'
                    ||-||||-|||||||||-|-
5' UGCUCCUGGAAACAAUAGGCAUGGCUGGCAUUGGUACAAGGAUCCACGGU 3'
                    pen_C2791-2

                    pen-miR2118e
3'                  AUCCGUACCCACCGUAACCCUU              5'
                    |||||||||-|||||||||-|-
5' AGUGCUCCUGGAAACAAUAGGCAUGGCUGGCAUUGGUACAAGGAUCCACGGU 3'
                    pen_C2791-2

                    pen-miR2118p
3'                  AUCCGUACCCACCGUAGCCCUU              5'
                    |||||||||-||||||:||-|-
5' AGUGCUCCUGGAAACAAUAGGCAUGGCUGGCAUUGGUACAAGGAUCCACGGU 3'
                    pen_C2791-2
```

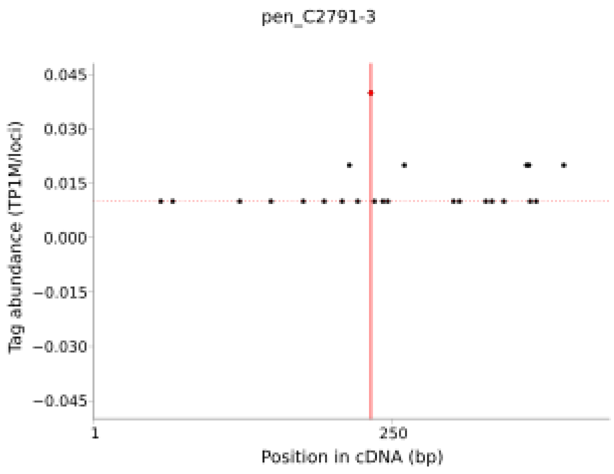


```
                 pen-miR482a
3'           CCUUACCCACCGUAACCUUU            5'
             ||-||||-|||||||||-|-
5' UGCUCCUGGAAACAAUAGGCAUGGCUGGCAUUGGUACAAGGAUCCACGGU 3'
                 pen_C2791-3

                  pen-miR2118e
3'           AUCCGUACCCACCGUAACCCUU            5'
             |||||||||-|||||||||-|-
5' AGUGCUCCUGGAAACAAUAGGCAUGGCUGGCAUUGGUACAAGGAUCCACGGU 3'
                  pen_C2791-3

                  pen-miR2118p
3'           AUCCGUACCCACCGUAGCCCUU            5'
             |||||||||-||||||:||-|-
5' AGUGCUCCUGGAAACAAUAGGCAUGGCUGGCAUUGGUACAAGGAUCCACGGU 3'
                  pen_C2791-3
```

miR529

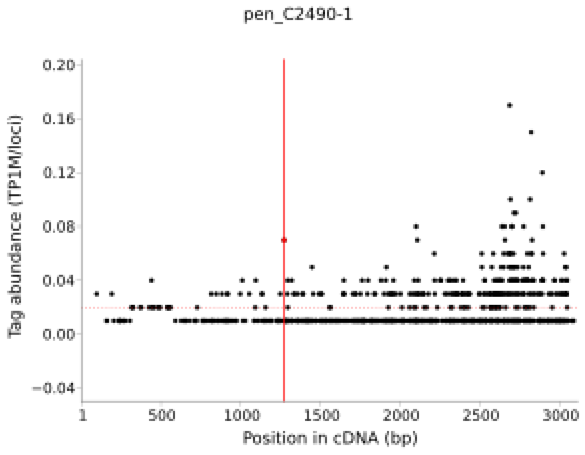


```
                  pen-miR529e
3'           CCCGACAUGAGAGAGAGAAGA            5'
             |||||--|||||||||||||-
5' CUUGGUGCUGCAUAUUCGGGCUUCACUCUCUCUCUUCGAUUUGUACCGGUU 3'
                  pen_C2490-1
```

miR536

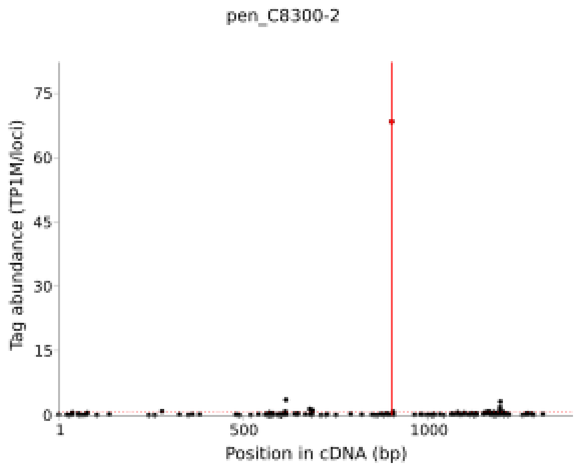

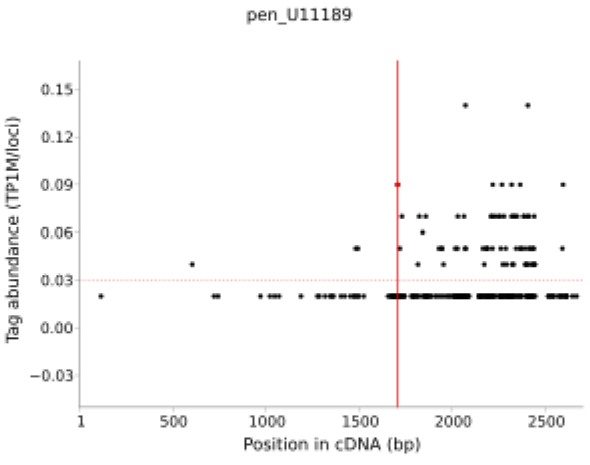


```
                  pen-miR536
3'           CUACGUGUGUCGAACUGUGCU            5'
             -|-||||||||||||:||||:
5' GUGAUAUCUUUGUCUCCAAAGCACACAGCUUGGCACGGAGGUGAGCCGUCU 3'
                  pen-miR536c
3'           CUUCGUGUUUCGAACCGUGCU            5'
             -|||||||-|||||||||||:
5' GUGAUAUCUUUGUCUCCAAAGCACACAGCUUGGCACGGAGGUGAGCCGUCU 3'
                  pen_C8300-2
```

```
                  pen-miR536c
3'           CUUCGUGUUUCGAACCGUGCU            5'
             -|||:-|:||||||||:|||:
5' CCAGAGGACAGAUUGGAAAAGUCCGAAGCUUGGUACGGAGUUUCCUCACCA 3'
                  pen_U11189
```

miR902

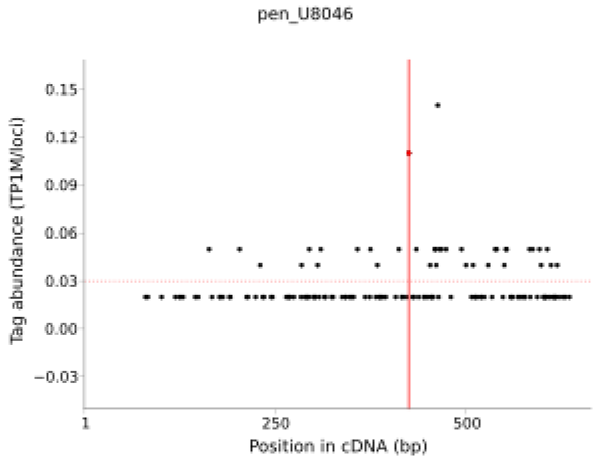


```
                 pen-miR902a
3'                 CGAUACUACGUCUGGAAGC              5'
                   ||-||-|-||-||||||||
5' GGUAGCAAGGAAAUUACGCAAUCA-GC-GACCUUCGGUAGCUCCAAGUC 3'
                 pen_U8046
```

miR904

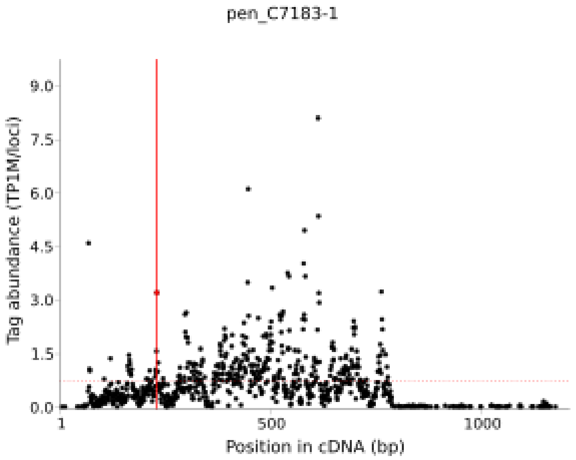


```
                     pen-miR904a
3'                  ACGGGGAUUUGUAACUGUUCU               5'
                    |-|||:|:||||-|||-||||
5' AUUGAUCUCUUACAUCCUCCCCUUGAACA-UGAGAAGAGGAAGCACAAGCU 3'
                     pen_C7183-1
```

miR1063

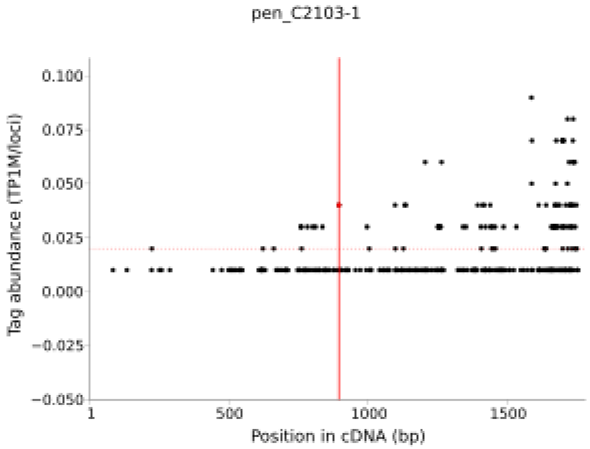


```
                    pen-miR1063a
3'                  UUCUACGUCAUAAGAUUC             5'
                    |||||||||-||-||:||
5' CAACAGAACUUCAGGAGAAGAUGCAGGAUCCUGAGAAGGGCCAGGAUG 3'
                    pen_C2103-1
```

miR1216

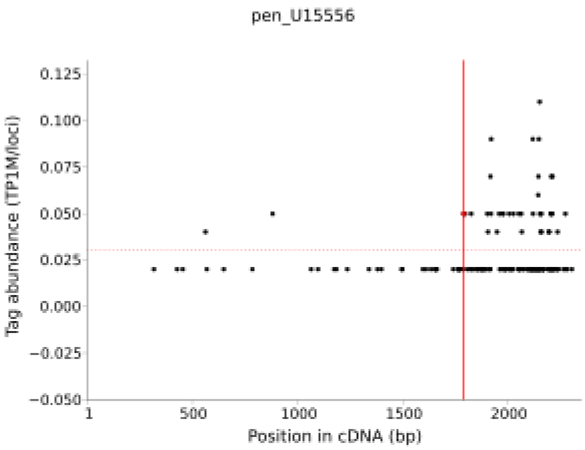


```
                        pen-miR1216
3'                  GUGUUCGUGUAGUGGUAG            5'
                    :||||||||:||||-||-
5' UAUUUGUGGAGGUUCAUUACAAGCACGUCACGAUGUUCAACUACAACU 3'
                        pen_U15556
```

miR1222

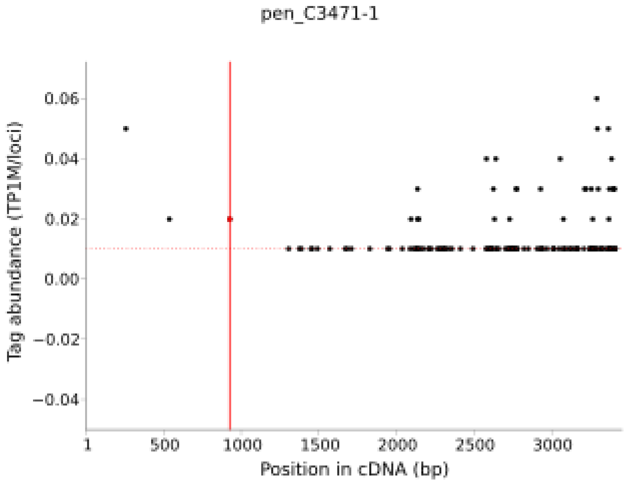


```
                        pen-miR1222a
3'                  AUUUGGUUGCUUGAGGAAG                5'
                    -|||:|||||-|:|||||:
5' GAGAGUUCAACGACCUCCAAAUCAACG-AUUCCUUUGCUGGCCAUCAAU 3'
                        pen_C3471-1
```

miR1863

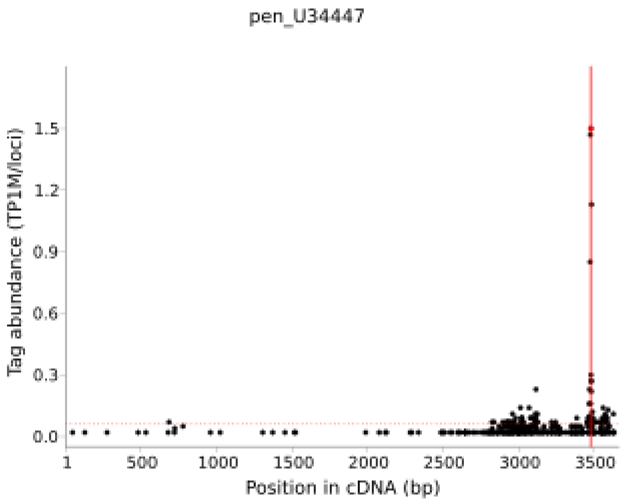


```
                      pen-miR1863b.2
3'                  CAUUACGGAAGUCGGUUC              5'
                    |-|:|-:||||||||||:|
5' UCGAAGUACUCUUUGAAGGAGU-UCUUCAGCCAGGCCUAGGAAGUGCU 3'
                        pen_U34447
```

miR1874

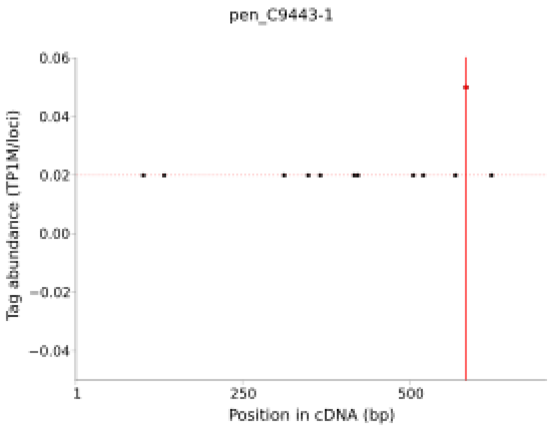

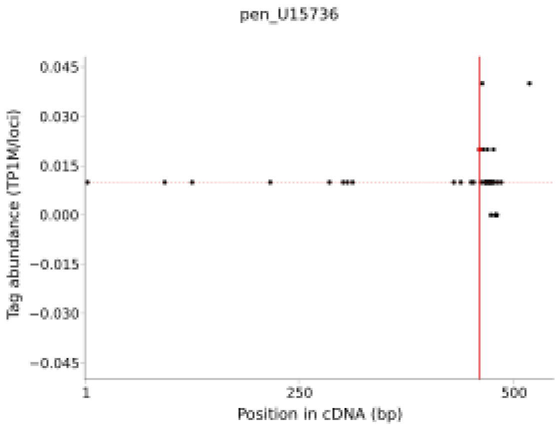


```
                        pen-miR1874
3'                   AGACCACAUCAUCGGGAU                 5'
                     |-|||-|||-|||||:|:
5' UUUGAGUUCGACCGCUAUGUGG-GUAAUAGCCUUGUUCACAUGUAUAG 3'
                        pen_C9443-1
```

```
                        pen-miR1874
3'                   AGACCACAUCAUCGGGAU                 5'
                     ||--|-|||||||||:|:
5' CUGGGAGUCCGAUCUGUUCGUGAGUAGUAGCCUUGUUGACAUGUAUGC 3'
                        pen_U15736
```

miR2108

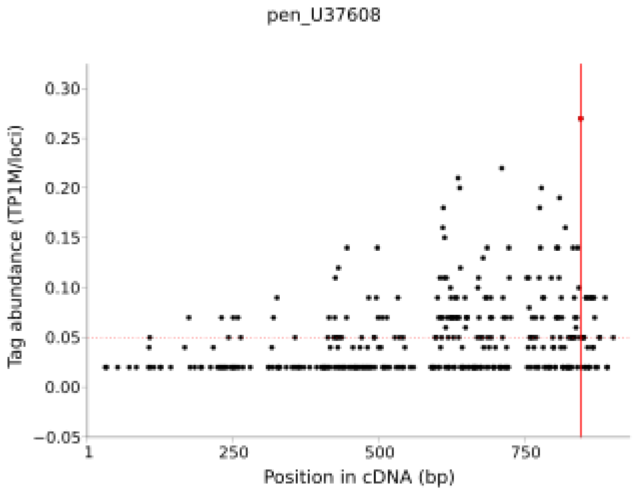


```
                        pen-miR2108a
3'                   GUUUGUGGUUGUGUUAUU                 5'
                     |-||:||||:||-||||-
5' AUGGACUUCUUAACACUCCAAUACCAGCAGAAUAUACAAUUAUGUUGA 3'
                        pen_U37608
```

miR2111

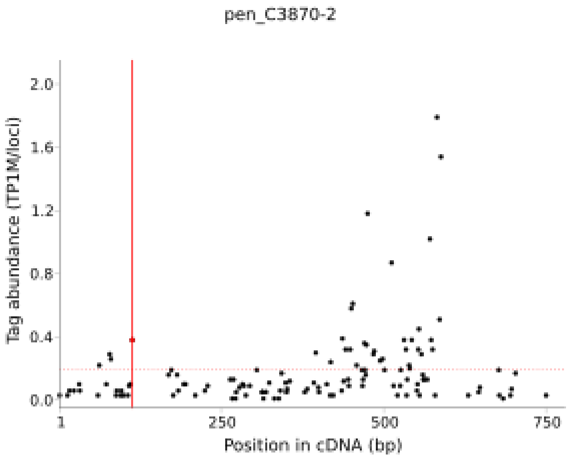

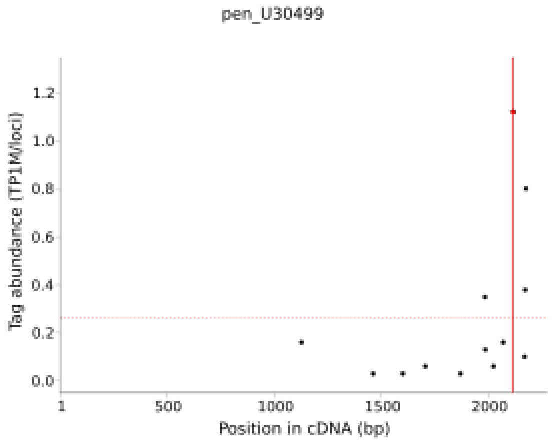


```
                        pen-miR2111
3'                   UCAUUAGACGUAGGUCUC                 5'
                     |-|-||:||-||:|||||
5' GUCAGCGUGGACGGAUUAUUCAUUUGAAUUCAGAGUUGGGACACAACC 3'
                        pen_C3870-2
```

```
                        pen-miR2111
3'                   UCAUUAGACGUAGGUCUC                 5'
                     :||-||:||-||||||-|
5' CCUUGAUUUGGAAGCUGGGUCAUUUGGAUCCAGUGCAAGUCAGGGCCU 3'
                        pen_U30499
```

miR2118

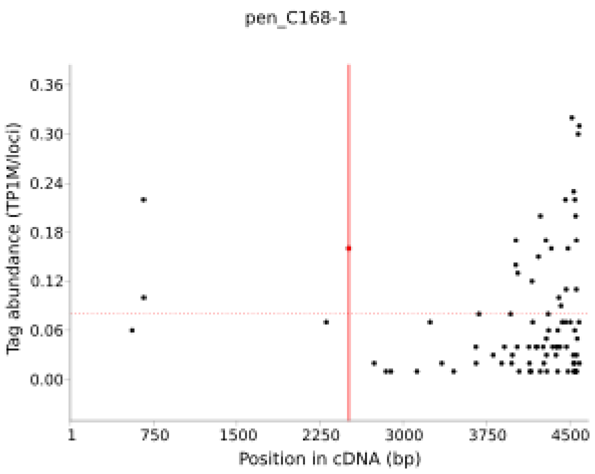

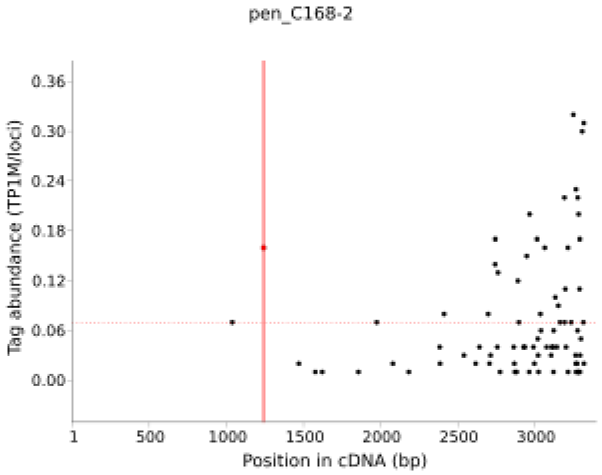


```
                    pen-miR2118a
3'                  AUCCGUACCCACCGUAGCUCUU                5'
                    -|||||||||||||||-|:||-
5' GAUGACAGGGAUGGUGUGAGGCAUGGGUGGCAUGGGGAUGAUGCUCGCCACA 3'
                    pen-miR2118e
3'                  AUCCGUACCCACCGUAACCCUU                5'
                    -|||||||||||||||-||||-
5' GAUGACAGGGAUGGUGUGAGGCAUGGGUGGCAUGGGGAUGAUGCUCGCCACA 3'
                    pen-miR2118p
3'                  AUCCGUACCCACCGUAGCCCUU                5'
                    -|||||||||||||||-||||-
5' GAUGACAGGGAUGGUGUGAGGCAUGGGUGGCAUGGGGAUGAUGCUCGCCACA 3'
                    pen_C168-1
```

```
                    pen-miR2118a
3'                  AUCCGUACCCACCGUAGCUCUU                5'
                    -|||||||||||||||-|:||-
5' GAUGACAGGGAUGGUGUGAGGCAUGGGUGGCAUGGGGAUGAUGCUCGCCACA 3'
                    pen-miR2118e
3'                  AUCCGUACCCACCGUAACCCUU                5'
                    -|||||||||||||||-||||-
5' GAUGACAGGGAUGGUGUGAGGCAUGGGUGGCAUGGGGAUGAUGCUCGCCACA 3'
                    pen-miR2118p
3'                  AUCCGUACCCACCGUAGCCCUU                5'
                    -|||||||||||||||-||||-
5' GAUGACAGGGAUGGUGUGAGGCAUGGGUGGCAUGGGGAUGAUGCUCGCCACA 3'
                    pen_C168-2
```

miR2673

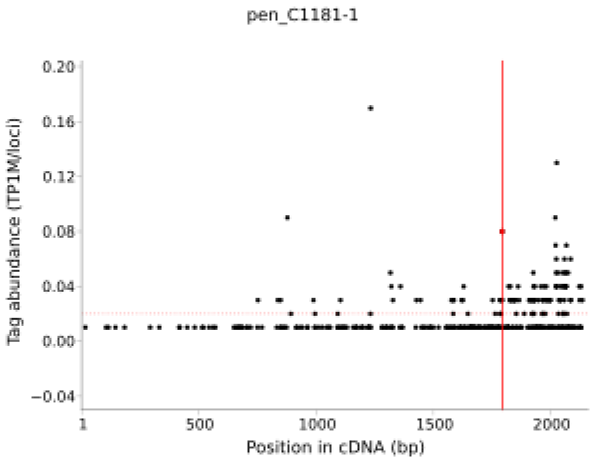


```
                    pen-miR2673a
3'                  CUUCUCCUUCUCCUUCUCAG                5'
                    |::|||||:|||||:||||:
5' AAGCUGAGCAAGUUAGUGGGGAGGAGGAGGAGGAGUUCUCAGGUUCAGGA 3'
                    pen_C1181-1
```

miR3509

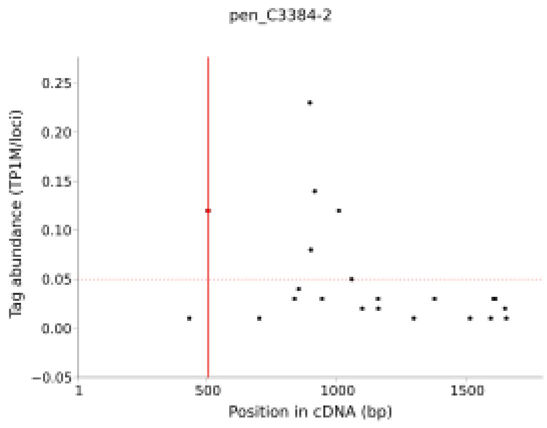


```
                    pen-miR3509
3'                  ACCACAGACUCUCAGCAA                5'
                    ||||-|-|||||||:|||
5' GUGUAGGGCGAUUUGUUUGGUUUGUGAGAGUUGUUUGUAUAACGGGGA 3'
                    pen_C3384-2
```

miR3948

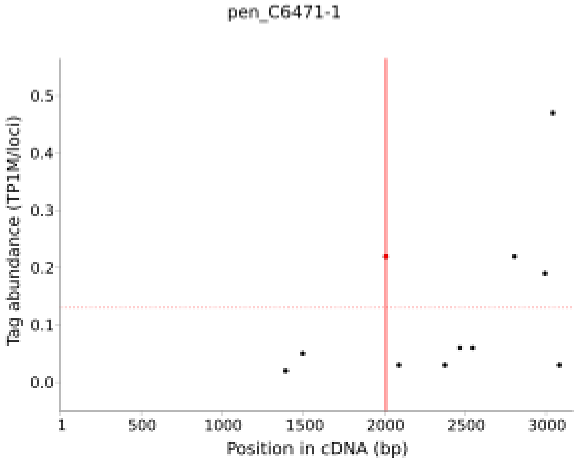


```
              pen-miR3948
3'         GUGGGAGGAGGGUCAGGG              5'
           ||||||-|||||||||||
5' UUGCUGCUCUUGCUGCCCACCCU-CUCCCAGUCCCUGCCAGAGUGCUG 3'
              pen_C6471-1
```

miR4414

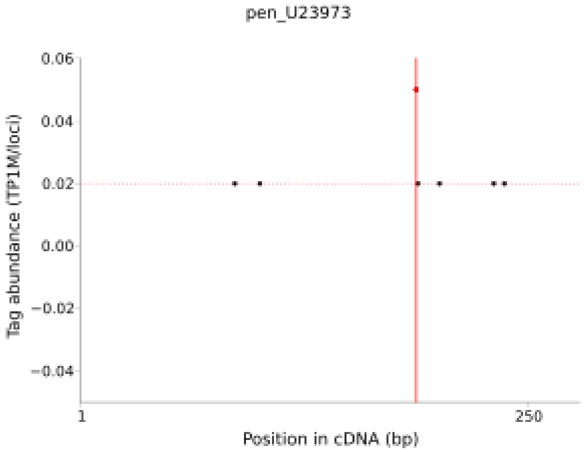


```
               pen-miR4414
3'         CUCGGCUGCUCAGUCGUCG              5'
           |:|::||-||||:|||||-
5' UGAAGAUUCUCUUACCAGGGUUGAAGAGUUAGCAGACAAGAAGACUGAC 3'
               pen_U23973
```

miR5021

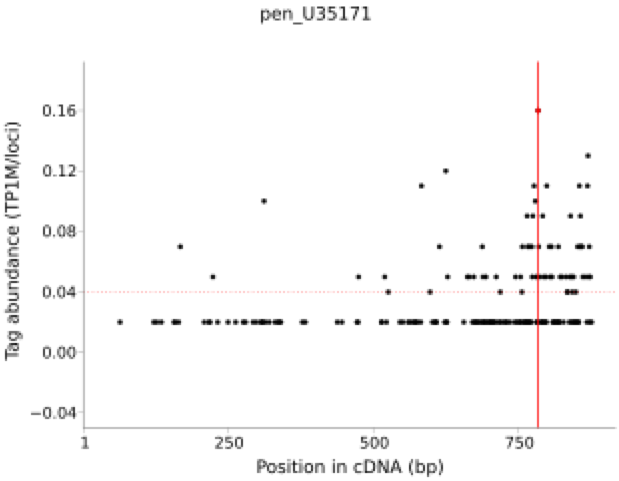


```
                 pen-miR5021
3'           AAACAAGAAGAAGAACAGU              5'
             ||--||-|||||||||||:
5' AAAUCCAACCCAUGAAUUUCAUUGUUCUUCUUGUCGUUGUAUCAUACUC 3'
                 pen_U35171
```

miR5269

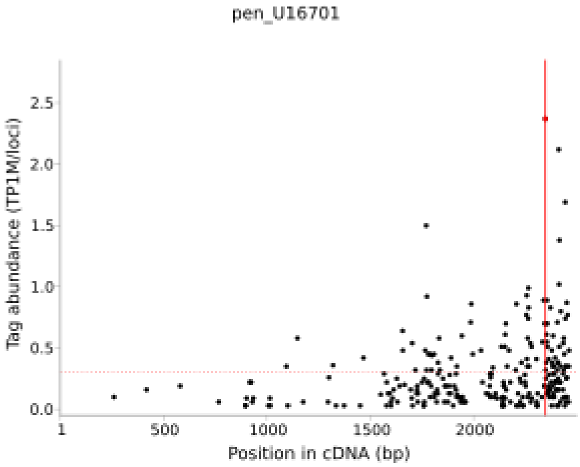


```
                        pen-miR5269a
3'                   AGUUAGAUAUAAGGUGGU                5'
                     |-|||--||||||||:||
5' UUUUAUCCUUUCCCUGCUGAAUGAAUAUUCCAUCACUGAUCUAUACGA 3'
                         pen_U16701
```

miR5298

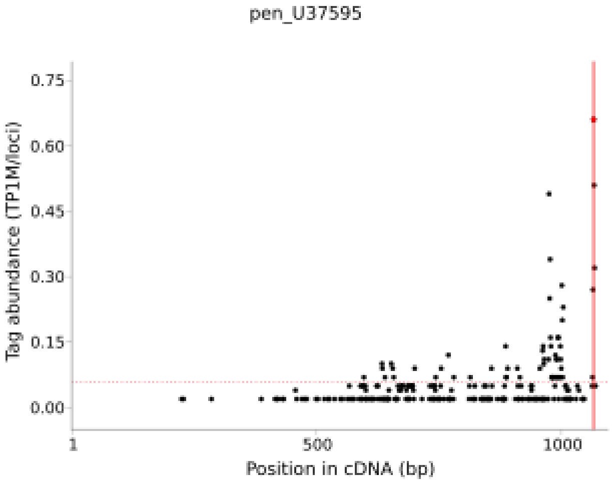


```
                         pen-miR5298b
3'                   UAGUAGAAGUAUGAGUAGAGG               5'
                     ||-||-||:|||:||||:|:|
5' AGAAUAAACGAUCAUGGAUAAUGUUUAUAUUCAUUUUCCAUCGAUCAUGUU 3'
                          pen_U37595
```

miR5562

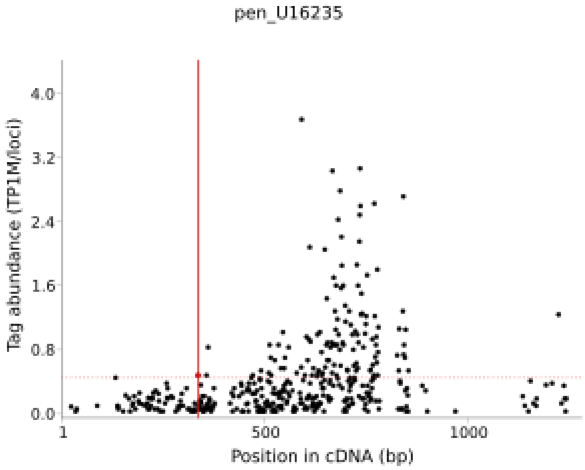


```
                            pen-miR5562
3'                   CAACGUCGGCAGAGUUGUAGUAA                5'
                     |||-||-||-|||||-|||||||
5' GGUGGCCGCGCACUGGGGUUUCAUCCCUCUCAUCAUCAUUAUUGGCAUGCGUU 3'
                            pen_U16235
```

miR5673

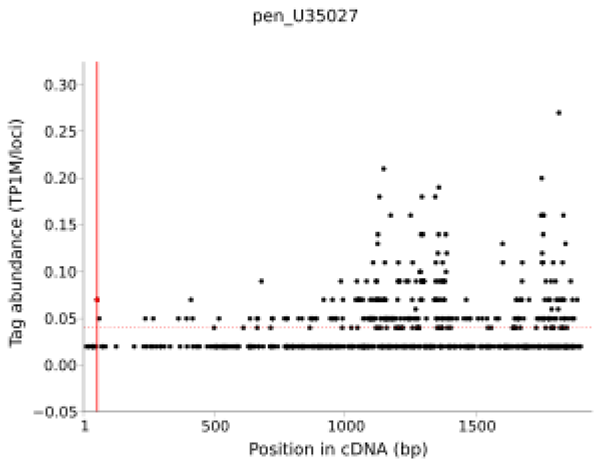


```
                 pen-miR5673
3'            ACGGAAAGCGCUCUAAGGU              5'
              |--|||||||-||||||-|
5' ACUGGGAAGUCCUUCGUUUGCUUUCGC-AGAUUCGAGCGAUUCCCGGCA 3'
                 pen_U35027
```

miR5754

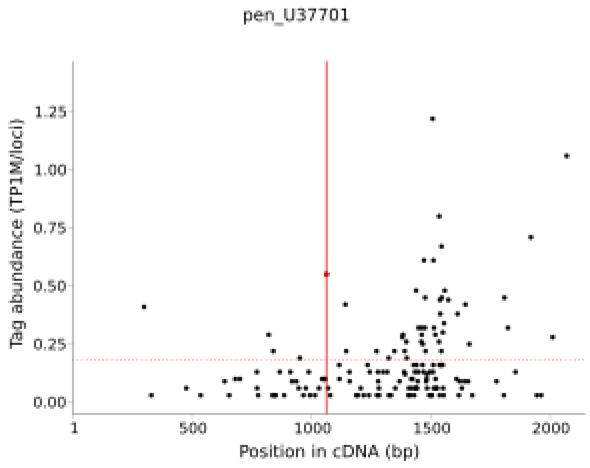


```
                 pen-miR5754
3'            ACCUUCUAGUCGCGUUAU              5'
              ||||||||||-|:|:|:
5' AAUUGAAAAUGAAACCAUGGAAGAUCAGAGUAGUGGGACAAAGCCCAU 3'
                 pen_U37701
```

miR5815

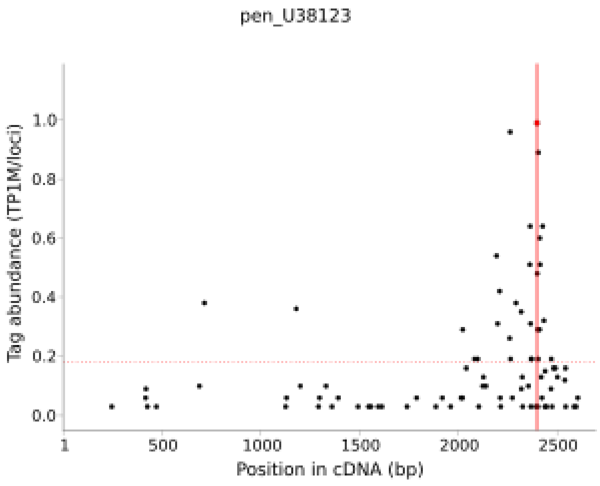


```
                 pen-miR5815
3'            UCAGUAGAUCCACAGGUAU              5'
              |-|-||:|||||-|||||:
5' UUGAUCAAGAUGCACUAACUGAUUUAGGUCUCCAUGGAGAUCUAGAAAA 3'
                 pen_U38123
```

miR6116

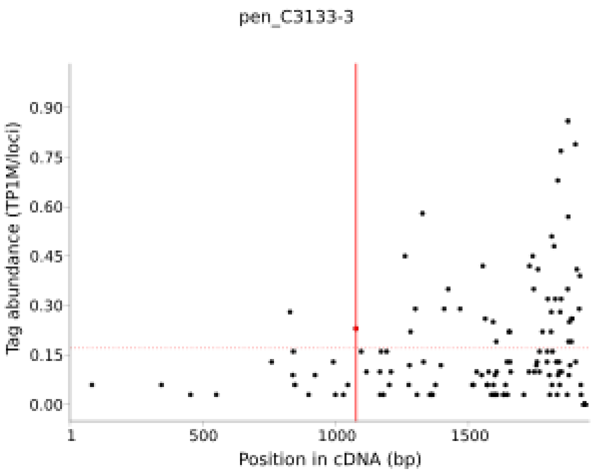

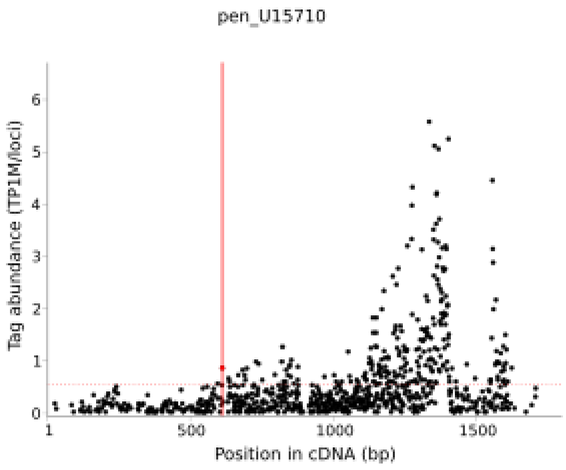


```
                   pen-miR6116
3'               GAGCACGAACACUAGGUU              5'
                 |-:|-|:|||||:||||:
5' UAUCUUUGGACACACACCAUGGGUUUGUGGUCCAGUACAGCAUGGAUC 3'
                   pen_C3133-3
```

```
                   pen-miR6116
3'                GAGCACGAACACUAGGUU               5'
                  :-||-|||||-||||||:
5' UCGGGCCCAACGAGCCAUCCGAGCUUGCGAUCCAGCAGAACGCCCAAG 3'
                   pen_U15710
```

miR6463

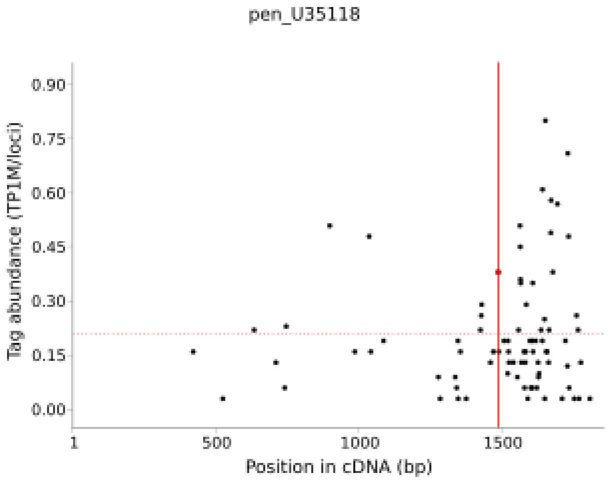


```
                   pen-miR6463
3'               ACGGUUGAAACUAGUAGG               5'
                 |-|||-:||-|:||||||
5' CAAGUUCUCAUGGUUGUUACCACUUUCGGUCAUCCCUUACUUCGGCCC 3'
                   pen_U35118
```

miR6486

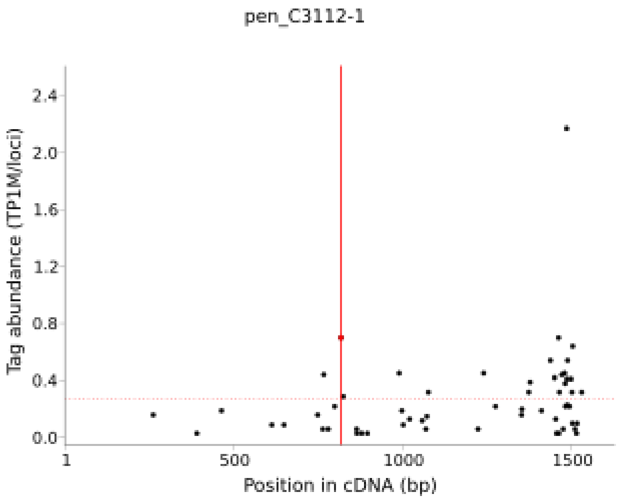


```
                   pen-miR6468
3'               ACCCAGGGGCCUUAGUGA               5'
                 ||-|-|:||||||||:||-
5' GCGAGAACCUCUUCGUCUGUGCCUCCGGAAUUACACCGAUGAAGAGCU 3'
                   pen_C3112-1
```

miR7755

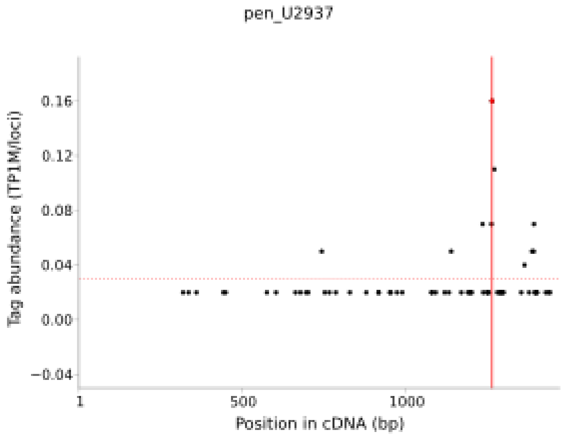


```
                    pen-miR7755
3'                AGUGAAGAUGUGGCAUUU              5'
                  |-||-|:||:|:|||||:
5' ACUCCAAAACUCCAUACUGACAUUUAUAUCGUAAGCAGUCACCUUCAG 3'
                     pen_U2937
```

Table S6 Targets for the novel miRNAs that were identified in *P. endiviifolia* and confirmed by degradome data. Below the table, T-plots for each identified target are presented.

miRNA family	miRNA species	Target	Domains	Molecular function	Cleavage site	Cleavage region
pen-miR8156	a,b,c	pen_U16671	-	-	1432	CDS
	a	pen_U37136	Armadillo/beta-catenin-like_repeat<br>U-box_domain	protein_binding<br>ubiquitin-protein_ligase_activity	1325	CDS
	b	pen_C1236-1	NB-ARC_domain	ADP_binding	1229	CDS
pen-miR8158		pen_U7542	Auxin_response_factor (family)<br>AUX/IAA_family<br>B3_DNA_binding_domain	DNA_binding	1479	CDS
pen-miR8159	a,b	pen_U15389	Polyketide_cyclase_/_dehydrase_and_lipid_transport (family)	-	1346	3'UTR
pen-miR8164		pen_C3350-1	-	-	3079	CDS
		pen_C4288-3	Cytochrome_b5-like_Heme/Steroid_binding_domain<br>Oxidoreductase_FAD-binding_domain<br>Oxidoreductase_molybdopterin_binding_domain<br>Oxidoreductase_NAD-binding_domain<br>Mo-co_oxidoreductase_dimerisation_domain	heme_binding<br>electron_carrier_activity<br>oxidoreductase_activity<br>molybdenum_ion_binding	2282	CDS
		pen_U7166	CBS_domain<br>SIS_domain (family)	adenyl_nucleotide_binding<br>carbohydrate_binding	1290	CDS
pen-miR8165		pen_U41210	BRCA1_C_Terminus_(BRCT)_domain (family)	-	1978	CDS
pen-miR8166		pen_U2587	AP2_domain	sequence-specific_DNA_binding_transcription_factor_activity	242	CDS
pen-miR8170		pen_U38378	HAUS_augmin-like_complex_subunit_4 (family)	-	2062	3'UTR
pen-miR8171		pen_U30616	PDZ_domain	protein_binding	996	CDS
pen-miR8173		pen_U31475	NUDIX_domain	hydrolase_activity	1074	CDS
pen-miR8176		pen_C11045-1	TPR_repeat	-	448	CDS

miR8156

pen_U16671

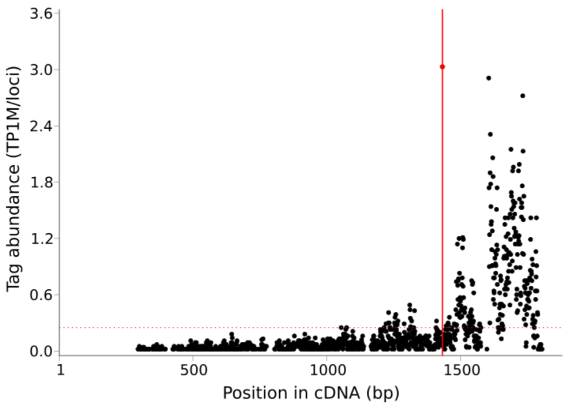


```
UCUCCCACACAUCGUCUAGGA pen-miR8156a
UCUCCCACACAUCGUCUAGGC pen-miR8156b
UCUGCCACACAUCGUCUAGGA pen-miR8156c
```

```
                          pen-miR8156a
3'                  AGGAUCUGCUACACACCCUCU              5'
                    ||:||-||||||||||-||:
5' UGUAGAUUUUGCAGGAUUCUUAAACGAUGUGUGGAAGGAAAAAACGACAGC 3'

                          pen-miR8156b
3'                  CGGAUCUGCUACACACCCUCU              5'
                    -|:||-||||||||||-||:
5' UGUAGAUUUUGCAGGAUUCUUAAACGAUGUGUGGAAGGAAAAAACGACAGC 3'

                         pen-miR8156c
3'                  AGGAUCUGCUACACACCGUCU              5'
                    ||:||-||||||||||-||:
5' UGUAGAUUUUGCAGGAUUCUUAAACGAUGUGUGGAAGGAAAAAACGACAGC 3'
                           pen_U16671
```

pen_U37136

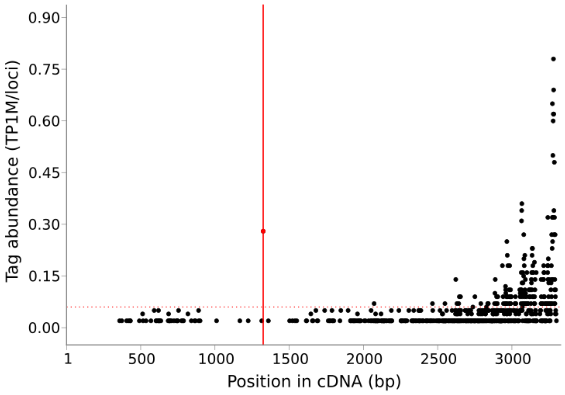

pen_C1236-1

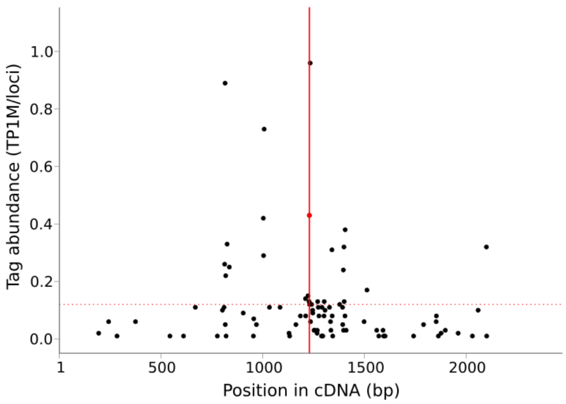


```
                          pen-miR8156a
3'                  AGGAUCUGCUACACACCCUCU              5'
                    ||||-|||||-|||-|||||:
5' GCAGAGAAUUAAGCAUCUCCUCGACGACGUGAGGGAGGGUAGUUGCUUGUG 3'
                           pen_U37136
```

```
                          pen-miR8156b
3'                  GAUCUGCUACACACCCUCU                5'
                    ||-||:|||||-||||||:
5' AGAACAUCUUAAUUAUACUCGAUGAUGUAUGGGAGGAAGAGUUUCUCCA 3'
                          pen_C1236-1
```

miR8158

pen_U7542

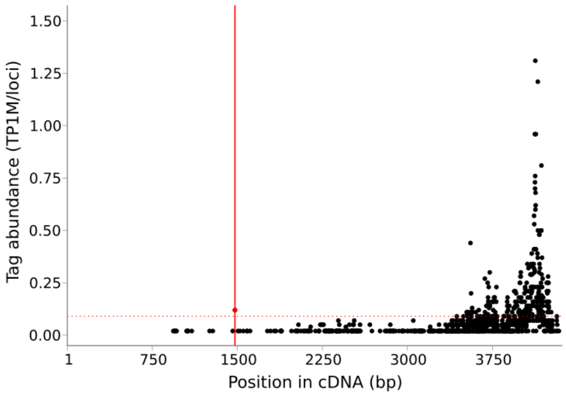


```
                           pen-miR8158
3'                  AGCUCGUCUUACGCAGGAAGU              5'
                    ||||||||:-|||||||||||
5' UUUGCCACAAGGCCACAUCGAGCAGGUUGCGUCCUUCAAUAAUCAGGACGU 3'
                            pen_U7542
```

miR8159

pen_U15389

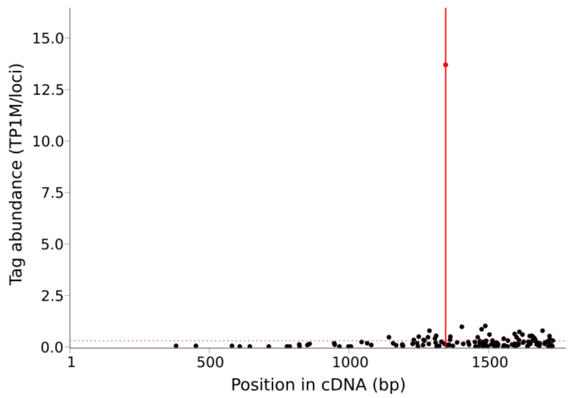


```
UUACCUUGAAGAGUCUGGAAG pen-miR8159a
UUACCUUGAAGAGUCUGGAA  pen-miR8159b

                   pen-miR8159a
3'                    GAAGGUCUGAGAAGUUCCAUU              5'
                      :|||::|||||||||||||-
5' CCCUGCCAGGCCUUCAAUUUCUGGACUCUUCAAGGUACUUCGUAUUAGCUC 3'

                  pen-miR8159b
3'                   AAGGUCUGAGAAGUUCCAUU              5'
                     |||::|||||||||||||-
5' CCUGCCAGGCCUUCAAUUUCUGGACUCUUCAAGGUACUUCGUAUUAGCUC 3'
                   pen_U15389
```

miR8164

pen_C3350-1

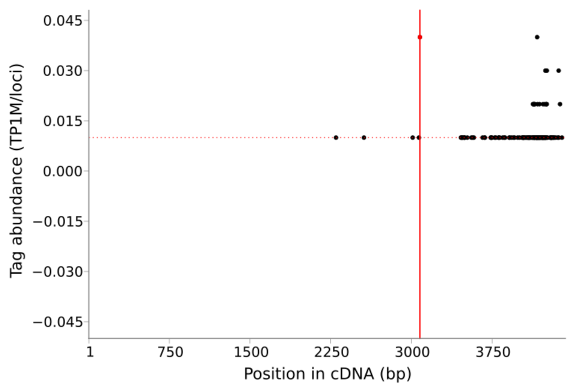

pen_C4288-3

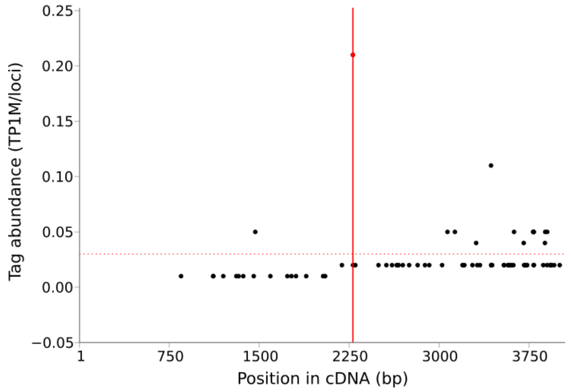


```
                   pen-miR8164
3'              CCCCUUCAAACGGUGUUUAAC                 5'
                ||-||:||||||-|||||||-
5' UUGGGGAGCCUGGCAAAGGAGAGGUUUGCGACAAAUUUGCUCGAGGGAAAA 3'
                   pen_C3350-1
```

```
                   pen-miR8164
3'              CCCCUUCAAACGGUGUUUAAC                 5'
                -|-|||||||||||-|||||-
5' CACUCCCACUCUGGGCCUGAGAAGUUUGCCAGAAAUUCCCUUAUCUGAAGU 3'
                   pen_C4288-3
```

pen_U7166

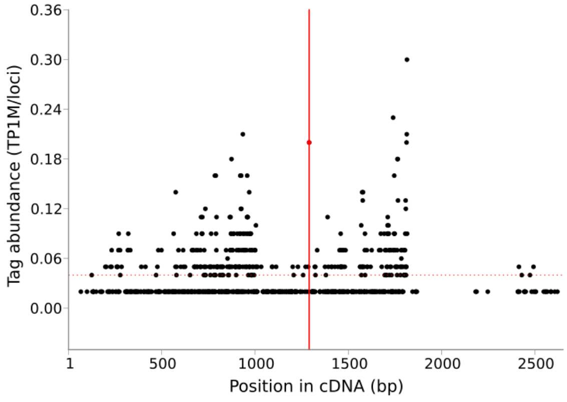


```
                   pen-miR8164
3'              CCCCUUCAAACGGUGUUUAAC                 5'
                |||||:-|||||-|||:|||-
5' UCUUCAAGAUGUCAGUCGGGGAGCUUUGCAACAGAUUACCGAGAACGAUCU 3'
                   pen_U7166
```

miR8165

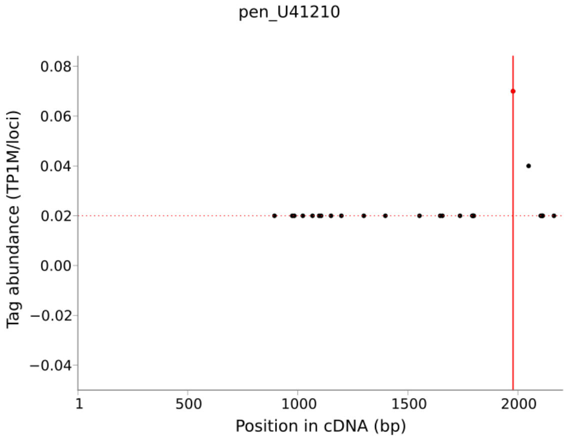


```
                    pen-miR8165
3'               CUUUCAGGAGAGAUCGUU               5'
                 -|||||-|||||||||||
5' CAGAAGUGCAGCCGCCAAAAAGUACUCUCUAGCAACCAUAUGGGGAAA 3'
                    pen_U41210
```

miR8166

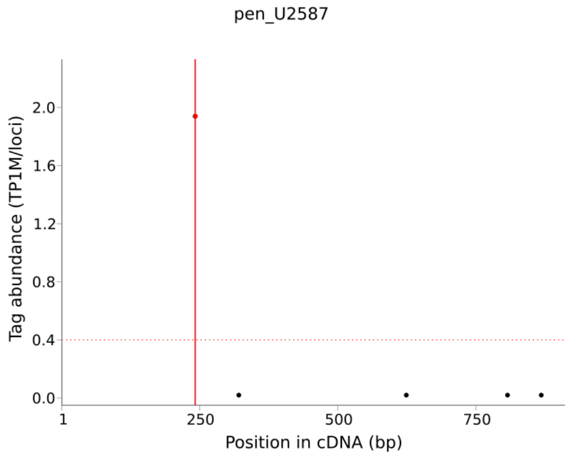


```
                    pen-miR8166
3'                UGACUACAGAGGGUUUCGUU               5'
                  |-||||-|||||||||||||
5' CCACCUCCACCAGGAACAAUGAUUUCUCCCAAAGCAAUUCAAGAUGCUGC 3'
                    pen_U2587
```

miR8170

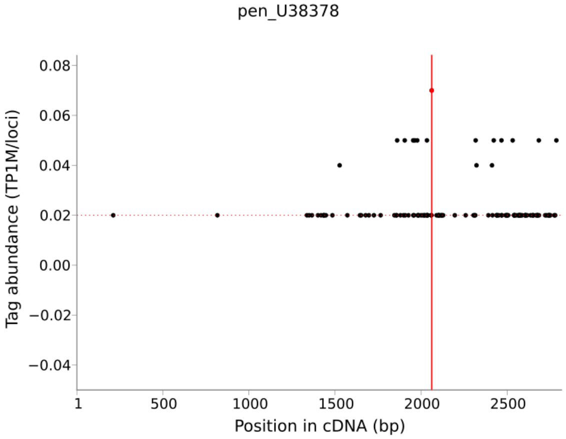


```
                    pen-miR8170
3'                UACGUAGAGGAGACACUUCGU               5'
                  |||||-||::|||||||:||-
5' CAUACUGUUACUGAGCAAUGCAGCUUUUCUGUGAGGCUCACUCCCGCUUCA 3'
                    pen_U38378
```

miR8171

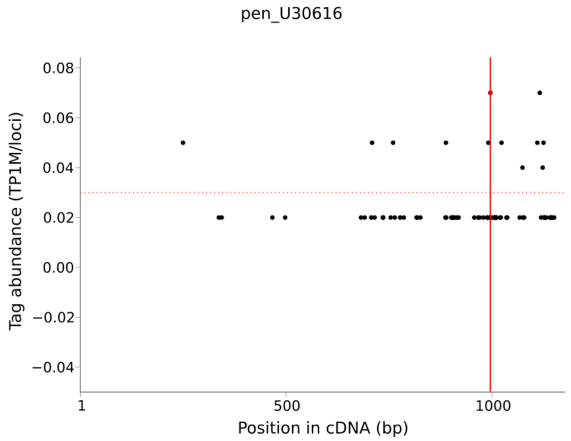


```
                   pen-miR8171
3'            GAGUCAGGGUACGAAGAUAAC            5'
              --||-|||||||||||-||||
5' UUGCAGCGGCACAGAAUACCA-UCCCAUGCUUCCAUUGAUCUUCUGCAAGA 3'
                   pen_U30616
```

miR8173

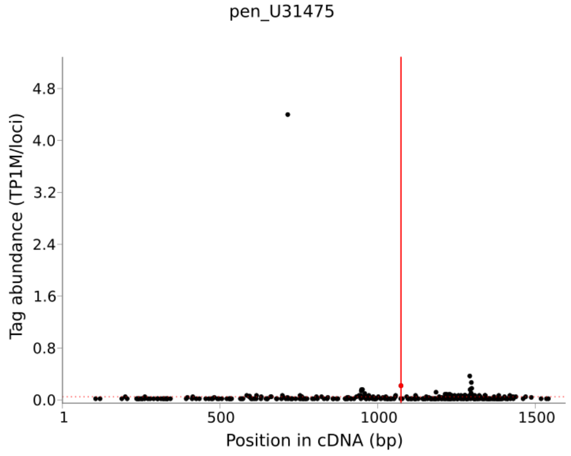


```
                   pen-miR8173
3'              UGCUAGUGUAGAGGAUGACC            5'
                |||||||||||--||||||-
5' AGUGCAGGACGUAUUUGACGAUCACAUCAACUACUGUGCCCCCUUCUGCA 3'
                   pen_U31475
```

miR8176

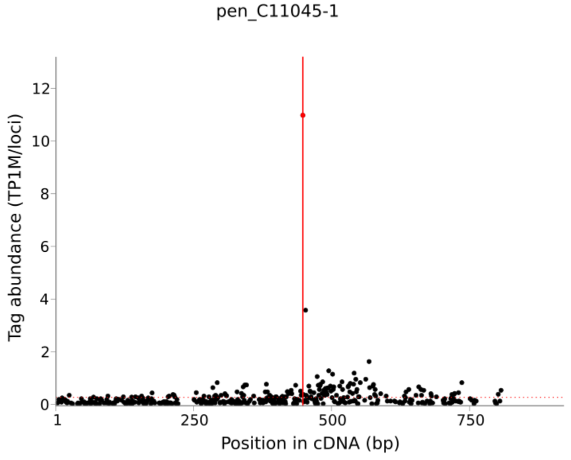


```
                   pen-miR8176
3'              AAACUCCAGGACUUUGUGUCU            5'
                |--||||||||||||||:|||
5' AUCUUGGAGGGGAAGACUACGAGGUCCUGAAACAUAGAGGUUAUGUGAAAU 3'
                   pen_C11045-1
```

References:

Addo-Quaye C, Snyder JA, Park YB, Li YF, Sunkar R, Axtell MJ. 2009. Sliced microRNA targets and precise loop-first processing of *MIR319* hairpins revealed by analysis of the *Physcomitrella patens* degradome. *RNA* **15(12)**: 2112–2121.

Altschul SF, Madden TL, Schäffer AA, Zhang J, Zhang Z, Miller W, Lipman DJ. 1997. Gapped BLAST and PSI-BLAST: a new generation of protein database search programs. *Nucleic Acids Research* **25**: 3389-3402.

German MA, Luo S, Schroth G, Meyers BC, Green PJ. 2009. Construction of Parallel Analysis of RNA Ends (PARE) libraries for the study of cleaved miRNA targets and the RNA degradome. *Nature Protocols* **4(3):** 356–362.

Kruszka K, Pacak A, Swida-Barteczka A, Stefaniak AK, Kaja E, Sierocka I, Karlowski W, Jarmolowski A, Szweykowska-Kulinska Z. 2013 Developmentally regulated expression and complex processing of barley pri-microRNAs. *BMC Genomics* **14**:34.

Pant B, Musialak-Lange M, Nuc P, May P, Buhtz A, Kehr J, Walther D, Scheible WR. 2009. Identification of nutrient-responsive Arabidopsis and rapeseed microRNAs by comprehensive real-time polymerase chain reaction profiling and small RNA sequencing. *Plant Physiology* **150**: 1541-1555.

Provan J, Murphy S, Maggs CA. 2004. Universal plastid primers for Chlorophyta and Rhodophyta. *European Journal of Phycology* **39**: 43-50.

Ruhfel BR, Gitzendanner MA, Soltis PS, Soltis DE, Burleigh JG. 2014. From algae to angiosperms-inferring the phylogeny of green plants (Viridiplantae) from 360 plastid genomes. *BMC Evolutionary Biology* **14: 23.**

Sierocka I, Rojek A, Bielewicz D, Karlowski W, Jarmolowski A, Szweykowska-Kulinska Z. 2011. Novel genes specifically expressed during the development of the male thalli and antheridia in the dioecious liverwort *Pellia endiviifolia*. *Gene* **485**: 53-62.

Simon L, Lalonde M, Bruns TD. 1992. Specific amplification of 18S fungal ribosomal genes from vesicular-arbuscular endomycorrhizal fungi colonizing roots. *Applied and Environmental Microbiology* **58**: 291-295.
